# Reassessment of the Triassic archosauriform *Scleromochlus taylori*: neither runner nor biped, but hopper

**DOI:** 10.7717/peerj.8418

**Published:** 2020-02-19

**Authors:** S. Christopher Bennett

**Affiliations:** Department of Biological Sciences, Fort Hays State University, Hays, KS, USA

**Keywords:** Archosauriformes, Anatomy, Locomotion, Scotland, Lossiemouth

## Abstract

The six known specimens of *Scleromochlus taylori* and casts made from their negative impressions were examined to reassess the osteological evidence that has been used to interpret *Scleromochlus’*s locomotion and phylogenetic relationships. It was found that the trunk was dorsoventrally compressed. The upper temporal fenestra was on the lateral surface of skull and two-thirds the size of the lower, the jaw joint posteriorly placed with short retroarticular process, and teeth short and subconical, but no evidence of external nares or antorbital fossae was found. The posterior trunk was covered with ~20 rows of closely spaced transversely elongate dorsal osteoderms. The coracoid was robust and elongate. The acetabulum was imperforate and the femoral head hemispherical and only weakly inturned such that the hip joint was unsuited to swinging in a parasagittal plane. The presence of four distal tarsals is confirmed. The marked disparity of tibial and fibular shaft diameters and of proximal tarsal dimensions indicates that the larger proximal tarsal is the astragalus and the significantly smaller tarsal is the calcaneum. The astragalus and calcaneum bear little resemblance to those of *Lagosuchus*, and the prominent calcaneal tuber confirms that the ankle was crurotarsal. There is no evidence that preserved body and limb postures are unnatural, and most specimens are preserved in what is interpreted as a typical sprawling resting pose. A principal component analysis of skeletal measurements of *Scleromochlus* and other vertebrates of known locomotor type found *Scleromochlus* to plot with frogs, and that finding combined with skeletal morphology suggests *Scleromochlus* was a sprawling quadrupedal hopper. Phylogenetic analyses found that *Scleromochlus* was not an ornithodiran, but was either within the Doswelliidae or outside the clade consisting of the most recent common ancestor of the Erythrosuchidae and Archosauria and all its descendants.

## Introduction

Among the tetrapod fossils from the Carnian Lossiemouth Sandstone Formation near Elgin, Scotland, those of *Scleromochlus taylori*, a tiny archosauriform with extremely long hindlimbs known from seven largely articulated skeletons on small slabs, have probably had the greatest impact on interpretations of diapsid evolution. The first specimens were briefly described and named by Woodward (1907), who interpreted *Scleromochlus* as a small bipedal running or leaping dinosaur. Huene (1914) described the specimens more thoroughly and interpreted *Scleromochlus* as an arboreal climbing and leaping pseudosuchian close to the origin of pterosaurs. Swinton (1960), Brodkorb (1971) and Martin (1983) discussed *Scleromochlus* in relation to the origin of birds, whereas Padian (1984) suggested that Huene had it only half right and interpreted *Scleromochlus* as a digitigrade bipedal cursor close to the origin of pterosaurs and dinosaurs, a view that has gained general acceptance (Gauthier, 1986; Sereno, 1991; Benton, 1999; Fraser, 2006; Brusatte et al., 2010). Despite that, Bennett (1996, 1997) argued that Huene had only the other half right and Padian had it all wrong and that *Scleromochlus* was an arboreal leaper not close to pterosaurs.

The specimens have not been easy to study. They are quite small and the bone tissue was poorly preserved and usually lost such that the specimens are natural molds. It has been necessary to study positive impressions made from the specimens’ negative impressions. Woodward (1907) made “wax-squeezes” of the specimens, whereas Huene (1914) made clay and glue casts. Subsequent authors studied positive impressions made of polyvinyl chloride (PVC) plastic and polyurethane and room temperature vulcanizing silicone rubbers (referred to as molds by Padian (1984), peels by Sereno (1991) and casts by Benton (1999)). The sandstone has not helped; variously described as fine-grained (Huene, 1913), medium-grained (Benton & Walker, 2011), and coarse-grained (Gauthier, 1984), its grains can be seen adhering (sometimes in clumps!) to the PVC and silicone rubber, indicating that each set of positive impressions made resulted in changes to the impressions. Illustrating the specimens has also been difficult. Huene (1914) and Benton (1999) published photographs of the sandstone slabs, but they merely demonstrate that the slabs existed and do not provide detailed information about the osteology of *Scleromochlus*. Woodward (1907) illustrated some specimens in a detailed lithograph, but other authors published drawings: Huene (1914) presented many small, hatched line drawings of skeletal elements; Padian (1984) and Sereno (1991) each illustrated a few elements with stipple drawings; and Benton (1999) illustrated all the specimens with sketch-like line drawings with occasional coarse stippling that provide moderate detail. The drawback to the drawings is that they show what the authors thought the specimens preserved and not necessarily what was actually preserved.

I examined the specimens briefly in 1988 and spent more time in the early 1990s studying a set of silicone rubber peels provided to L.D. Martin, and found both rather difficult to interpret. As a result, I had little confidence in the various published interpretations of the osteology, locomotion, and relationships of *Scleromochlus*, and it seemed to me that interpretations of its osteology were often fitted to preconceived notions of relationships and locomotion rather than interpretations of relationships and locomotion being deduced from the osteology. Things might have been left so, but in 2013 I came to suspect that Bennett (1997), too, had it at least half wrong. By happy coincidence, I had shortly before perfected my technique for studying small slab specimens, so I took another look at the evidence and after several years of study gained some confidence in interpreting the specimens. This article is not a thorough redescription of the osteology of *Scleromochlus* but rather is a reassessment of the osteological evidence that has been used to interpret *Scleromochlus’*s mode of life, locomotion, and phylogenetic relationships. A principal component analysis of skeletal measurements of *Scleromochlus* and other vertebrates of known locomotor type was done to examine the locomotion of *Scleromochlus*, and it was found to plot with frogs. Based on osteological evidence, including previously overlooked evidence from the specimens, and the principal component analysis, *Scleromochlus* is interpreted as a sprawling quadrupedal hopper analogous to frogs. Phylogenetic analyses found that *Scleromochlus* was not an ornithodiran, but rather either within the Doswelliidae or outside the clade consisting of the most recent common ancestor of the Erythrosuchidae and Archosauria and all its descendants.

### Literature review

**Figure 1:**
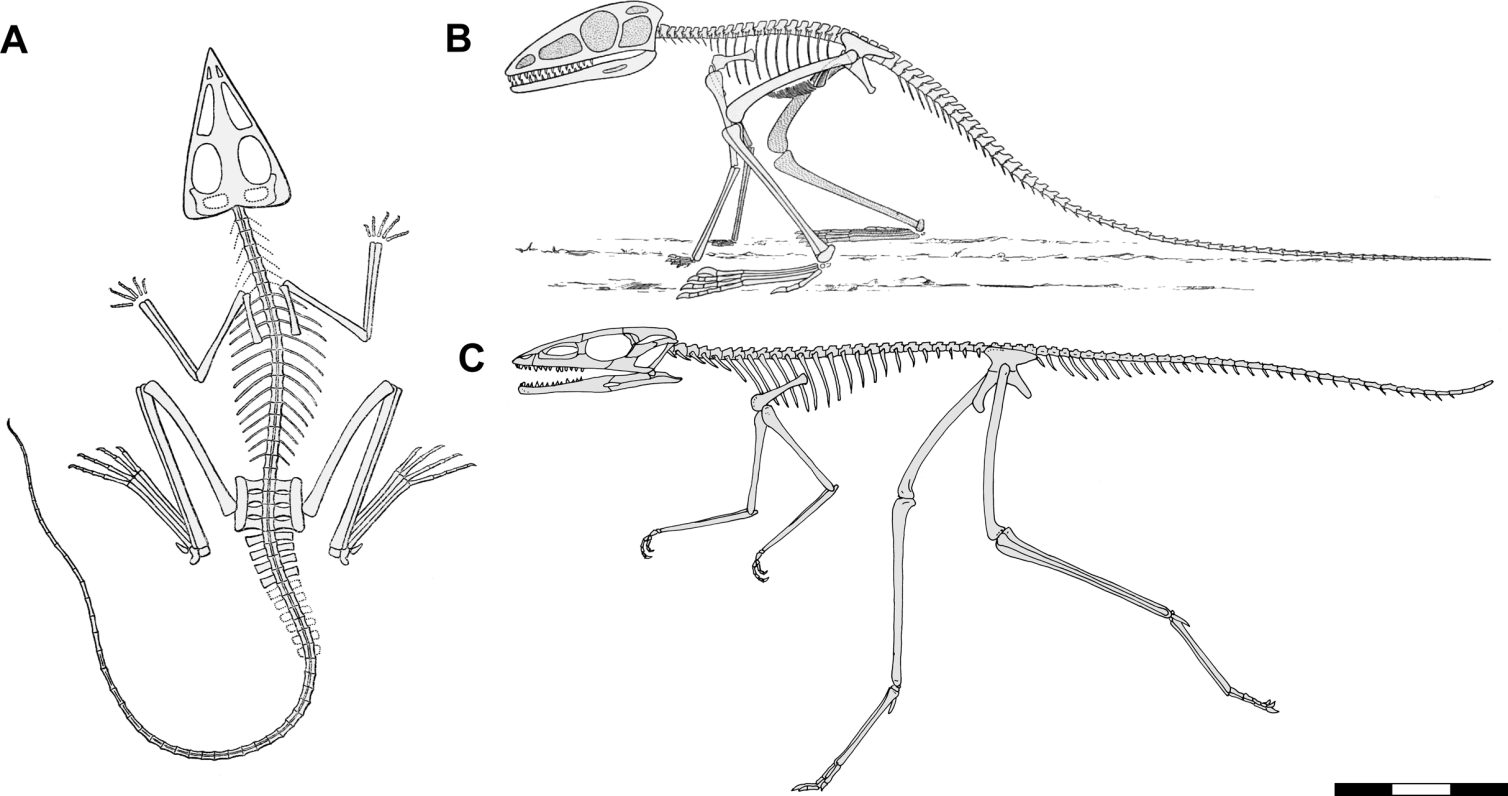
Skeletal reconstructions of *Scleromochlus taylori*. (A) Dorsal view from Woodward (1907). (B) Left lateral view from Huene (1914: fig. 33). (C) Left lateral view redrawn after Benton (1999: fig. 14). Scale bar for *C* = 3 cm, whereas A and B are scaled to similar sizes.

Woodward (1907) named *Scleromochlus taylori* based on descriptions of three specimens; two in William Taylor’s private collection, the holotype specimen (now NHMUK R3556) that preserved an articulated partial skeleton on part and counterpart slabs and a second (now NHMUK R3557) that preserved somewhat more poorly an articulated skeleton on part and counterpart slabs, and NHMUK R3146 that preserved two articulated skeletons lying side by side on part and counterpart slabs. A lithograph illustrated the dorsal slab of NHMUK R3556, the anterior half of the dorsal slab of NHMUK R3146, enlarged details of the tail, scattered chevrons, and a wax impression of the calcaneum of NHMUK R3557, and an enlarged detail of a wax impression of the right pes of NHMUK R3556. A skeletal reconstruction illustrated *Scleromochlus* in dorsal view with its long hindlimbs folded and directed anterolaterally at ~40° (Fig. 1A). Woodward described *Scleromochlus* as having a large skull with small nares, large antorbital fenestra and orbit, short neck and trunk, a plastron of closely spaced fine gastralia, four sacral vertebrae, slender tail, slender scapula, long hollow limb bones with the hindlimb twice as long as the fore, small manus, calcaneum with tuber, metatarsals I–IV slender, equilength and “fixed together,” and short metatarsal V. Note that Woodward’s use of plastron was presumably not a comparison to the ventral shell of chelonians but rather referred to the metal plate worn under a hauberk, comparable to Lambe’s (1917) use of cuirass to describe the gastralia of *Gorgosaurus*. Woodward stated that most characters agreed with dinosaurs such as the Triassic *Anchisaurus* and *Hallopus* (Marsh, 1896; *Hallopus* is now considered a derived cursorial crocodylomorph, Norell & Storrs, 1989) and interpreted *Scleromochlus* as a small dinosaur despite the undinosaur-like poses of the specimens and his skeletal reconstruction. Based on the marked limb length disparity and very long hindlimbs, Woodward (1907: pp. 140, 144) concluded that *Scleromochlus* was “adapted for a bipedal running or leaping gait” and commented that its “high degree of specialization (was) truly astonishing” in a Triassic dinosaur. In brief discussion comments appended to the article, C.W. Andrews concurred and suggested comparison to jerboas living in sandy environments, whereas A.P. Young asked whether *Scleromochlus* might have had a patagium for gliding flight.

Huene (1908: pp. 390–392) briefly discussed *Scleromochlus* and illustrated the palate, sacrum and pelvic girdle based on examinations of Woodward’s specimens. He compared it primarily to *Ornithosuchus* and *Erpetosuchus*, and considered it a pseudosuchian within the family Ornithosuchia. Subsequently, Huene (1914) published a thorough description of *Scleromochlus* based on further study of Woodward’s three specimens and another specimen in Taylor’s private collection (now NHMUK R3914) that preserved an articulated anterior part of a skeleton on part and counterpart slabs. Huene used positive impressions made of clay and glue (i.e., *Ton- und Leimabdrücken*) and strong low-angle artificial illumination to identify fine details that Woodward had overlooked (e.g., presence of teeth). A skeletal reconstruction illustrated *Scleromochlus* in left lateral view in an erect plantigrade quadrupedal pose, seemingly ready to leap (Fig. 1B). He again interpreted *Scleromochlus* as a pseudosuchian, but placed it in its own family, the Scleromochlidae.

Huene (1914) concurred with Woodward on many points (e.g., four sacral vertebrae, slender scapula, calcaneum with tuber) but described a long coracoid, large claws on the manus and pes, and metatarsals closely appressed but not fused. He argued that *Scleromochlus* was not adapted to crawling, striding, swimming, or hopping in the usual sense (the latter perhaps a reference to Andrews’ comparison to jerboas) and made no mention of bipedality. Instead, he interpreted the shoulder joint as highly mobile, which with the large claws suited *Scleromochlus* to “claw-climbing,” interpreted the long foot as plantigrade and suited to walking on branches, and interpreted the long hindlimbs as suited to leaping. Thus, Huene interpreted *Scleromochlus* as an arboreal climber and leaper, at home in the trees, leaping from branch to branch or tree to tree. In addition, he argued that the highly mobile shoulder joint and long forelimb could have been used to spread a patagium, allowing *Scleromochlus* to parachute to the ground. Based on the long coracoid, hindlimb proportions, tarsus (he compared the astragalus to the “astragalus” of the Pittsburgh *Campylognathus* (=*Campylognathoides*); but see Wellnhofer, 1974), foot with four closely appressed metatarsals and short metatarsal V, and the presumed parachuting membrane, Huene (1914) considered *Scleromochlus* to be close to the origin of pterosaurs.

Little work was done on *Scleromochlus* for nearly seventy years and all of it seems to have been based on Woodward’s (1907) and Huene’s (1908, 1914) descriptions rather than examinations of the specimens. Wilfarth (1949) dismissed Huene’s arboreal interpretation of *Scleromochlus* as naive and interpreted *Scleromochlus* as a link in an evolutionary chain leading up to large dinosaurs that craned their necks up to the air above as they sat or walked underwater. Thus, *Scleromochlus* was interpreted as walking underwater in Huene’s (1914) quadrupedal pose and propelling itself up to the surface by powerful extension of its long hindlimbs when in need of a breath of air. Young (1964) and Kuhn (1967) accepted *Scleromochlus* as the closest relative of pterosaurs. Swinton (1960) noted in a discussion of *Archaeopteryx* and bird origins that *Scleromochlus* was further along a hypothesized transition from pseudosuchians to dinosaurs than was *Ornithosuchus* and suggested that *Scleromochlus* might be dinosaurian. Brodkorb (1971) also briefly discussed *Scleromochlus* in the context of bird origins and viewed *Ornithosuchus* and *Scleromochlus* as dinosaurs not close to birds but representing morphological stages analogous to those that bird ancestors went through. Sharov (1971) compared *Scleromochlus* to *Podopteryx* (=*Sharovipteryx*), a small reptile with short forelimbs, very long hindlimbs, and traces of a gliding membrane spread by the hindlimbs, and concurred with Huene’s (1914) suggestion that *Scleromochlus* might have had such a gliding membrane.

Martin (1983: p. 111), who had been provided with a set of silicone rubber peels of the specimens, discussed *Scleromochlus* in relation to Walker’s (1972) proposal that birds and crocodiles shared a close common ancestor and stated that *Scleromochlus* and *Cosesaurus* shared long hindlimbs, pointed snouts, and “a remarkably high percentage of the features suggested to relate birds to coelurosaurs” including “a scapula parallel to the vertebral column in an avian manner.” Martin followed Woodward (1907) and Huene (1914) in interpreting the two large tarsals of *Scleromochlus* as an astragalus and a calcaneum with a distinct tuber and presented a skeletal reconstruction of *Scleromochlus* in a digitigrade bipedal pose with an upward slanting vertebral column quite similar to his later reconstruction of *Archaeopteryx* (Martin, 1991). He rejected a dinosaurian origin of birds, but suggested birds evolved from a small arboreal reptile with a tendency toward bipedality and leaping, and left open the possibility that *Scleromochlus* was related to birds.

Padian (1984) studied the *Scleromochlus* specimens and the PVC casts made by Walker, and made himself a set of polyurethane rubber casts. He commented that the interpretation of the ankle had been an obstacle to understanding *Scleromochlus’*s relationship to pterosaurs and dinosaurs, and reinterpreted the astragalus and calcaneum of Woodward (1907), Huene (1914) and Martin (1983) as medial and lateral distal tarsals, respectively. He illustrated the lateral distal tarsal of NHMUK R3556 and medial distal tarsal of NHMUK R3557 with stipple drawings and compared them to the distal tarsals of *Dimorphodon*. Padian suggested that Huene (1914) had it only half right, interpreted *Scleromochlus* as an erect bipedal cursor close to the origin of pterosaurs, and used that interpretation to support his interpretation of pterosaurs as erect digitigrade bipeds that evolved flight from the ground up. He stated that *Scleromochlus* and pterosaurs shared several characters (i.e., large head with very large fenestrae, strap-like scapula, short, deep trapezoidal pelvis, greatly reduced fibula, astragalus and calcaneum fused to the tibia, mesotarsal ankle, and four elongate, closely appressed metatarsals), and argued that *Scleromochlus* did not exhibit any arboreal characters and that its hindlimb morphology and proportions were evidence of digitigrade bipedal cursoriality.

Gauthier (1984: p. 117) included *Scleromochlus* in the cladistic analysis of diapsids in his doctoral dissertation and considered *Scleromochlus* to be the sister taxon of the Pterosauria. His list of characters shared by *Scleromochlus* and pterosaurs was similar to Padian’s (1984) but added characteristic metatarsal/tibia/femur ratio and enlarged nares and omitted strap-like scapula and short, deep trapezoidal pelvis. Subsequently in a publication based on the dissertation, he treated *Scleromochlus* as within the Pterosauria (i.e., “the sister-taxon to all other pterosaurs”, Gauthier, 1986: p. 16).

Benton & Walker (1985) suggested that *Scleromochlus* exhibited adaptations for saltation (e.g., short trunk, long tail, strengthened pelvis and long hindlimb) and for living on desert sand (e.g., naris with lateral flange, squamosal and quadratojugal with posterior flange protecting tympanic region). They compared it to jerboas and kangaroo rats, and dismissed Huene’s (1914) arboreal and Wilfarth’s (1949) aquatic interpretations but made no mention of Martin’s (1983), Padian’s (1984), or Gauthier’s (1984) suggestions of relationships to birds or pterosaurs.

**Figure 2:**
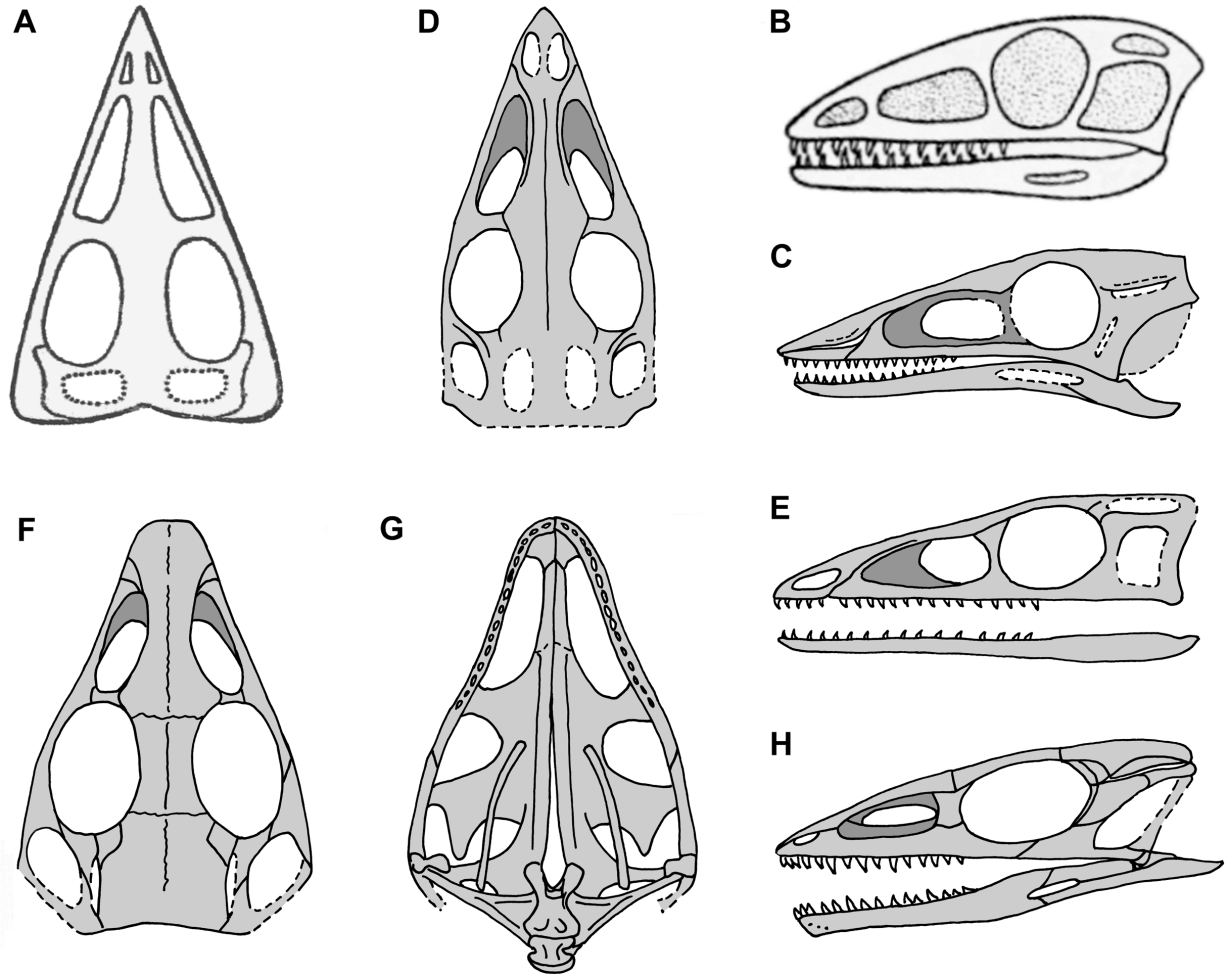
Skull reconstructions of *Scleromochlus taylori* in dorsal (A, D and F), palatal (G) and left lateral (B, C, E and H) views. (A) From Woodward (1907). (B) From Huene (1914: fig. 33). (C) Redrawn from Benton & Walker (1985: fig. 3g). (D and E) Redrawn from Sereno (1991: figs. 16a and 16b). (F, G and H) Redrawn from Benton (1999: fig. 8a and 8b). In (C)–(F) and (H) antorbital fossae are indicated with a darker gray. Scaled to uniform skull length.

Sereno (1991), who had made himself a set of latex rubber peels of the *Scleromochlus* specimens, discussed *Scleromochlus* in the context of a cladistic analysis of basal archosaurs. He presented stipple drawings of the proximal femur, distal tibia, tarsus, and metatarsus of NHMUK R3557 and the humerus of NHMUK R4823/4824, a fifth specimen donated by William Taylor to the NHMUK in 1921 that preserves an incomplete skeleton lacking a skull on part and counterpart slabs. The femur was illustrated with a prominent medially directed head, the tibia with fused astragalus, and Sereno followed Padian (1984) in interpreting the astragalus and calcaneum of Woodward (1907), Huene (1914) and Martin (1983) as distal tarsals, in this case the 3rd and 4th, respectively. He also presented a skull reconstruction (Figs. 2D and 2E) and a skeletal reconstruction in a digitigrade bipedal running pose. Sereno rejected most of the characters that Padian viewed as synapomorphies of *Scleromochlus* and pterosaurs, but stated that *Scleromochlus* and pterosaurs shared four synapomorphies (i.e., skull length >50% of presacral column length; scapula length <75% of humeral length; fourth trochanter absent; and metatarsal I length ≥85% of metatarsal III length). His cladistic analysis found *Scleromochlus* to be the probable sister taxon of the Pterosauria within an Ornithodira consisting of the common ancestor of the Pterosauria and Dinosauromorpha and all its descendants.

In an abstract of a meeting presentation, Padian, Gauthier & Fraser, (1995) argued that *Scleromochlus* exhibited no arboreal characters and supported the interpretation of *Scleromochlus* as a digitigrade bipedal cursor close to the origin of pterosaurs. However, they do not seem to have published a subsequent article.

Bennett (1996) argued that the hindlimb characters used by Padian (1984), Gauthier (1984, 1986) and Sereno (1991) to link *Scleromochlus*, pterosaurs, and dinosaurs were homoplastic in pterosaurs, and showed that reanalysis after a posteriori recoding or deletion of the homoplastic characters resulted in pterosaurs falling outside the crown-group Archosauria, whereas *Scleromochlus* remained the sister taxon of the Dinosauria + *Lagosuchus*. However, the recoding of characters was deemed inappropriate by other workers and the study was largely ignored (Parrish, 1997; Benton, 1999; Brochu, 2001). Subsequently, Bennett (2013) partitioned the 1996 data set, subjected the partitions to homogeneity testing, and demonstrated that a partition consisting of a suite of characters identified by Padian (1980, 1983) as correlated with cursorial locomotion was incongruent with other partitions and all other characters at the α = 0.01 probability level. The cause of the incongruence was determined to be homoplasy in pterosaurs. The incongruent characters were reevaluated and reformulated and when the revised data set was analyzed pterosaurs fell outside the crown-group Archosauria, whereas *Scleromochlus* remained close to dinosaurs.

Bennett (1997) concurred with Padian (1984) that Huene (1914) had it only half right, but disagreed as to which half it was and accepted that *Scleromochlus* as an arboreal leaper but interpreted it as not close to the origin of pterosaurs. I argued that pterosaurs exhibited a suite of characters associated with vertical clinging and leaping, but considered the similarities to *Scleromochlus* to be convergent because my 1996 cladistic analysis had shown that *Scleromochlus* was not closely related to pterosaurs. Note that the character codings for *Scleromochlus* used in my 1996 and 2013 cladistic analyses and the discussion of *Scleromochlus’*s morphology in my 1997 article were based on uncritical acceptance of Padian’s (1984) interpretation of *Scleromochlus’*s hindlimb morphology, Gauthier’s (1984, 1986) and Sereno’s (1991) codings of *Scleromochlus* from their cladistic analyses, and Huene’s (1914) interpretation of *Scleromochlu*s as an arboreal leaper rather than my own examinations of the specimens or casts.

Benton (1999) redescribed the osteology of *Scleromochlus* based on the six known specimens (the five mentioned above plus NHMUK R5589, a somewhat disarticulated partial skeleton on a dorsal slab) and PVC casts made by A.D. Walker. He illustrated all specimens, and presented a detailed skull reconstruction (Figs. 2F–2H). He described four small distal tarsals at the proximal end of the left metatarsus of NHMUK R3556 and interpreted the astragalus and calcaneum of Woodward (1907), Huene (1914) and Martin (1983) as calcaneum and astragalus, respectively, based on comparisons to the tarsus of *Lagosuchus* (Sereno, 1991; fig. 9). Benton rejected arboreal interpretations and interpreted *Scleromochlus* as a digitigrade bipedal cursor or jerboa-like saltator. He presented a skeletal reconstruction in a digitigrade bipedal running pose (Fig. 1C; drawn with pedal digit III longest though the text stated IV was longest) much like Sereno’s (1991), but stated that the proportions in Sereno’s reconstruction were incorrect (when the reconstructions are scaled to equivalent femur length, Benton’s presacral vertebral column and tail are ~9% shorter and ~11% longer, respectively). Note that the proportions in Benton’s reconstruction also differ markedly from those in Woodward’s (1907; Fig. 1A) and Huene’s (1914; Fig. 1B) reconstructions. Benton considered *Scleromochlus* and pterosaurs to share one synapomorphy (i.e., skull length >50% of presacral column length), and his cladistic analysis found *Scleromochlus* to be the sister taxon of the Ornithodira. Benton (2006) briefly mentioned *Scleromochlus*, again stating that it was bipedal and either a digitigrade cursor or jerboa-like saltator.

Recently, Headden (2011) presented a skeletal reconstruction of *Scleromochlus* in an erect digitigrade bipedal running pose with laterally compressed trunk, prominent medially directed femoral head, and metatarsal I much shorter than II–IV. Witton (2014; see also Witton, 2013) suggested in a blog post that *Scleromochlus* might have had a furry pelage and fuzzy feet for purchase on loose sand, and recast Benton’s (1999) skeletal reconstruction as a digitigrade bipedal jerboa-like saltator in flight. Ezcurra et al. (2017) presented a cladistic analysis that placed *Scleromochlus* in a tritomy with *Lagerpeton* and the Dinosauriformes based on a data matrix of 117 taxa and 676 characters enlarged from that of Ezcurra (2016) and Nesbitt et al. (2017; extended data figs. 7 and 8) reported on analyses of Nesbitt’s (2011) and Ezcurra’s (2016) data matrices with *Scleromochlus* added. In the strict consensus trees of the latter analyses *Scleromochlus* was also close to *Lagerpeton* and dinosauriforms, but the codings of *Scleromochlus* were not included in the Supplemental Information.

## Materials and Methods

All six specimens of *Scleromochlus taylori* (NHMUK R3146, R3556, R3557, R3914, R4823/4824 and R5589) were examined microscopically under strong low angle illumination and photographed as needed, as were the multiple brown PVC casts made by A.D. Walker in the 1960s (no longer in the RSM (=NMS) as stated by Benton (1999), but in the NHMUK), a set of off-white polyurethane rubber casts made in the late 1970s by K. Padian (YPM VP 58559–58566), and a set of white silicone rubber peels provided to L.D. Martin in the early 1980s (at one time numbered KUVP 63277–63285, but apparently unnumbered as of this writing). A set of rubber peels made by P.C. Sereno in the 1980s is in the UCRC at the University of Chicago, but I was prevented from accessing them; however, if latex rubber as stated by Sereno (1991), then the now ~30 year old peels have probably suffered considerable deterioration and may be unusable. It is not clear whether the clay and glue impressions made by F.V. Huene were permanent, but they do not seem to be at the NHMUK and were not found in the collections of the Institut für Geowissenschaften, Universität Tübingen, Tübingen, Germany (P. Havlik, 2014, personal communication) and presumably are lost. Note that here PVC and polyurethane positive impressions are referred to as casts because of their thickness and relative solidity and silicone rubber positive impressions are referred to as peels because of their thinness and flexibility.

Polyvinyl chloride casts and silicone rubber peels differ in the quality of their impressions presumably because of differing surface tensions of the materials: PVC casts are smoother than silicone peels in that they do not record the tiniest cracks between adjacent sand grains, whereas silicone peels have a rougher appearance because they do. Polyurethane casts are smoother than PVC ones, but they are soft and fragile such that high points can be distorted and smoothed by pressure. In my experience, Walker’s PVC casts are generally more informative than silicone rubber peels because they are the oldest extant impressions and so record the least degraded condition of the specimens, because of their smoothness, because their solidity preserves the marked relief of some specimens better than flimsy peels, and because Walker in some cases made casts before and after chipping away matrix and bone to expose further negative impressions (e.g., skull of NHMUK R3557 on dorsal slab, mandible of NHMUK R3556 on ventral slab). Despite that, later impressions may exhibit features differently or exhibit features that were not present on earlier ones. This seems to have resulted from the removal of the mulmy, clayey substance noted by Huene (1913) in some negative impressions or from the rupture of thin partitions between the natural mold and a previously unreachable cavity, permitting the casting material to take an impression of the newly reachable space.

*Scleromochlus* skeletons were not preserved crushed along a bedding plane as in lithographic limestones, and so only those elements or parts of elements that are intersected by the fracture planes are visible on specimens and casts. In addition, in many cases an indeterminate amount of matrix along the fracture plane seems to have been lost when or after the slabs were split such that elements visible as negative impressions on one slab may leave no trace on the other.

The positive impressions of casts and peels are described rather than the negative impressions of the natural molds of the slabs. Important features are illustrated with parallel viewing stereo pairs of photographs and interpretive drawings of the casts and peels, the former so that readers can evaluate what is preserved and the latter to illustrate how I interpret it. Note that the stereo pairs may exaggerate the relief of casts. The terms right and left in specimen descriptions refer to the *Scleromochlus* individual’s right and left. When looking at illustrations of casts of specimens (e.g., Benton, 1999: figs. 2–7), right and left appear normal in illustrations of dorsal slabs, whereas they appear reversed in illustrations of ventral slabs. Measurements reported, usually to the nearest 0.1 mm, were taken from casts and peels except as noted, and comparisons of such measurements with those of negative impressions on specimen slabs found no significant difference. Lengths of vertebral segments (=centrum + intervertebral cartilage) are based on the lengths of articulated series of vertebrae divided by the number of vertebrae in the series. The cervico-dorsal vertebral transition has been variously defined as between the last vertebra bearing ribs that do not articulate with the sternum and the first vertebra bearing ribs that do or as anterior to the pectoral girdle (Romer, 1956; Buchholtz & Stepien, 2009; Müller et al., 2010; and see comments in Bennett, 2014). Because it is not possible to determine which ribs articulated with the sternum, the cervico-dorsal transition is here interpreted as coinciding with the anterior margin of the glenoid region of the pectoral girdle. Lengths of selected skeletal elements of *Scleromochlus* specimens are shown in Table 1. Approximate preserved angles and ranges of motion of selected limb joints of *Scleromochlus* specimens are shown in Table 2. If the carcasses were intact when buried as Benton (1999) and this article suggest (see below), then compaction of the sand might have led to minor disarticulation and minor changes in the preserved angles of joints, but would be unlikely to have caused large changes in the position of the limb segments. The preserved angles of shoulder and hip joints were measured as the angle between the body midline and the long axis of the propodial. Those of elbow and knee joints were measured as the angle between the long axis of the propodial and the long axes of the epipodials. That of ankle joints was measured as the angle between the long axes of the tibia and fibula and the long axis of the metatarsus.

**Table 1:** Length measurements (in mm) of selected skeletal elements of *Scleromochlus taylori* specimens. NHMUK R3146A and B, R3556, R3557, R3914, R4823/4824 and R5589 taken from casts. Measurements in parenthesis are from Huene (1914). See text for discussion. Mt, metatarsal.

	R3146A	R3146B	R3556	R3557	R3914	R4823/4824	R5589
Humerus	(19.5)	15.4	(15.8)	21.9	18.9	16.8	–
Radius	(19.5)	–	–	–	17.7	–	–
Ulna	17.8	–	(16.5)	–	17.9	–	–
Femur	≥27.4	~26.8	(30)	(32)	–	≥25.2	–
Tibia	–	27.5	≥33.1	34.5	–	–	–
Fibula	–	–	33.1	–	–	29.6	33.0
Mt I–IV	–	–	16.1–17.1	18.6	–	–	17.1

**Table 2:** Approximate preserved angles and ranges of motion (in degrees) of selected limb joints of *Scleromochlus taylori* specimens. NHMUK R3146A and B, R3556, R3557, R3914, R4823/4824 and R5589. The measured angles of the left and right limb joints are separated by a slash and the range of motion (ROM) is the greatest angle minus the least angle. Shoulder and hip angles were measured relative to the body midline, whereas elbow, knee and ankle angles were measured between proximal and distal limb segments.

	R3146A	R3146B	R3556	R3557	R3914	R4823/4824	R5589	ROM
Shoulder	88/82	123/147	–/170	–/59	–/23	–/110	–/–	23–170
Elbow	87/78	–/43	–/3	–/–	–/45	–/29	–/16	3–87
Hip	26/90	68/14	43/12	–/13	41/–	–/3	35/41	3–90
Knee	0/68	30/0	14/0	33/13	19/–	–/0	0/16	0–68
Ankle	–/–	8/–	29/7	–/107	6/–	–/5	74/38	5–107

The above methodology, that is, the description of the positive impressions of casts and peels of the specimens, is the same as that used by all previous authors though I have studied more sets of impressions than they did and so have been able to follow physical changes to the specimens due to the production of multiple sets of positive impressions. Some reviewers deemed the manuscript unpublishable on the grounds that observation and interpretation of the positive impressions is no longer adequate, that I should redescribe on the basis of micro X-ray computed tomographic imaging, and that I should wait for new specimens to be found! I’ll let others micro-CT and wait for additional specimens, and here I counter previous interpretations using the standard methodology, though in many cases I include stereo-pairs to illustrate features and support my interpretations with explanations of my reasoning and more argumentation than previous authors.

In the discussion of locomotion, slow and fast terrestrial locomotion by alternating movements of the limbs is referred to as walking and running, respectively, whereas propulsion by simultaneous powerful extension of the hindlimbs that results in the animal becoming temporarily airborne is referred to as leaping. To distinguish between modes of leaping, the branch to branch and tree to tree leaping exhibited by extant gallagid and tarsiid primates (Napier & Walker, 1967) and proposed for *Scleromochlus* by Huene (1914) and for pterosaur ancestors by Bennett (1997) is termed arboreal leaping, the terrestrial continuous leaping of macropodid marsupials and dipodid and dipodomyine rodents is termed bounding, and the discontinuous leaping typical of quadrupedal anurans, hexapedal insects including caeliferans, ensiferans, cicadellids, and one blattellid (Picker, Colville & Burrows, 2012), and octopedal salticid spiders is termed hopping. Note that toads are capable of quadrupedal bounding (Reilly et al., 2015). Continuous leaping without marked changes of direction is termed straightaway, whereas leaping with rapid changes in direction is termed erratic.

In order to examine the locomotor behavior of *Scleromochlus*, a data set consisting of measurements of *Scleromochlus* reconstructions and skeletal measurements of selected theropod dinosaurs, which are interpreted as erect bipedal cursors (Christiansen, 1998), and representative extant vertebrates of known locomotor type was assembled (Supplemental Information). The extant vertebrates included frogs (quadrupedal hoppers), toads (quadrupedal bounders), lizards (quadrupedal runners), the lizard *Basiliscus* (habitually quadrupedal facultative bipedal runner), jerboas and kangaroo rats (bipedal bounders), and jumping mice (habitually quadrupedal but bound bipedally when startled). Note that jerboas, kangaroo rats, and jumping mice are included because *Scleromochlus* has been compared to bipedal bounders (Woodward, 1907; Benton & Walker, 1985; Benton, 1999), and frogs and toads are included because I noted marked similarity of body form and limb proportions between them and *Scleromochlus* and thought the comparison would be interesting. The measurements included: skull length, width and height; neck (=occiput to center of glenoid fossa measured along vertebral column), trunk (=glenoacetabular) and tail lengths (=center of acetabulum to posterior end of body or tail); humerus, antebrachium and manus lengths; pelvic girdle depth (perpendicular to the vertebral column); and femur, tibia, third hindlimb segment (usually metatarsus or tarsometatarsus, but tarsus only in anurans), and fourth hindlimb segment lengths (usually longest digit, but longest metatarsal and digit in anurans).

**Figure 3:**
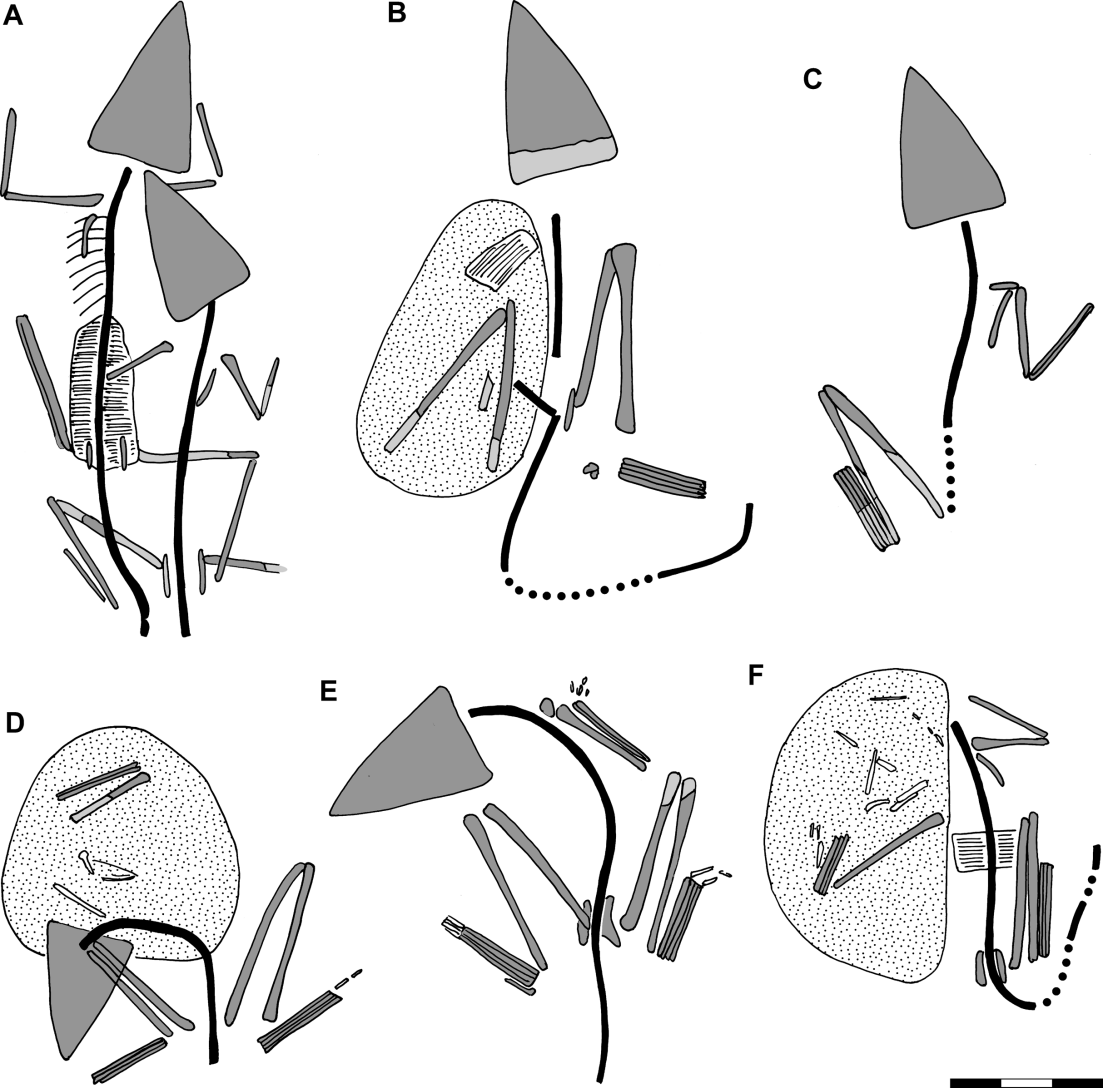
Preserved body positions of *Scleromochlus taylori* specimens in dorsal view based on combined information from dorsal and ventral slabs where present. Vertebral column in black; skull, limb girdles, propodials and epipodials with dark gray fill; dorsal osteoderms indicated by hatching; reconstructed parts indicated by black dots and light gray fill; and stippling denotes disturbed areas. For more detailed drawings, see Benton (1999; Figs. 2–7). (A) NHMUK R3146. (B) NHMUK R3557. (C) NHMUK R3914. (D) NHMUK R3556. (E) NHMUK R5589. (F) NHMUK R4823/4824. Scale bar = 3 cm.

Measurements of *Scleromochlus* were taken from the reconstruction in this article, from Benton’s (1999: fig. 14) reconstruction with the scale adjusted to best match measurements in his Table 1 and from Sereno’s (1991: figs. 16 and 18b) reconstruction with femur length scaled to match that of Benton’s reconstruction. Measurements of extant vertebrates were taken from specimens in museum collections. Ostrom (1969) noted that most theropods had femur:tibia length ratios of >1, but because *Scleromochlus* had a femur:tibia length ratio of <1 only measurements of theropods with femur:tibia length ratios of <1 were included. Measurements of the following specimens were taken from published descriptions:

- *Archaeopteryx lithographica*—Measurements of the Berlin (HMN 1880/1881) and Eichstätt (JM 2257) specimens were taken from Wellnhofer (2009).
- *Compsognathus longipes* (BSP AS I 563)—Measurements were taken from Ostrom (1978). Ostrom gave the skull length as “70–75” mm; 72.5 mm was used. Where Ostrom did not provide measurements, they were taken from his figures 3 and 14. Skull width was based on height:width ratio in *Archaeopteryx* in Wellnhofer (2009).
- *Sinornithoides youngi* (IVPP V9612)—Measurements were taken from Russell & Dong (1993). Where Russell & Dong did not provide measurements, they were taken from their figure 3 and comparisons to *Saurornithoides junior* in Osmólska & Barsbold (1990).
- *Sinornithomimus dongi* (IVPP V11797−10)—Measurements were taken from Kobayashi & Lü (2003). Where Kobayashi & Lü did not provide measurements, they were taken from their figure 4 and comparisons to *Dromiceiomimus brevitertius* in Osmólska & Barsbold (1990).
- *Struthiomimus altus* (AMNH 5539)—Measurements were taken from Osborn (1917). Where Osborn did not provide measurements, they were taken from his figure 5 and comparisons to *Dromiceiomimus brevitertius* in Osmólska & Barsbold (1990).

A reduced data set consisting of trunk and limb segment lengths, standardized by dividing by trunk length, was subjected to principal component analysis using IBM SPSS Statistics for Windows, version 24 (IBM Corp., Armonk, NY, USA). The results of the analysis are described and illustrated in bivariate plots of components in the “Discussion” section.

Phylogenetic analyses were done using PAUP* 4.0b10 (Swofford, 2003) and two different data matrices, Ezcurra’s (2016) 79 taxon data matrix and Bennett’s (2013) 19 taxon 134 character Updated Data Matrix. PAUP* is not as new as some other phylogenetics programs, but it is arguably the most-used and most-cited phylogenetics program, it has a wealth of tree search options and algorithms, and it successfully replicates Ezcurra’s (2016) 79 taxon 600 character data matrix using his NEXUS file, so there is no reason to think that the results of analyzing the 80 taxon matrix with *Scleromochlus* added with PAUP* would be less satisfactory than those of the 79 taxon matrix without *Scleromochlus*, “garbage in, garbage out” notwithstanding.

Ezcurra’s (2016) analyses were replicated. Ezcurra’s Analysis 1 in which species with potentially problematical hypodigms were coded only on the basis of their holotypes, Analysis 2 in which species with potentially problematical hypodigms were coded on the basis of their hypodigms, and Analysis 3 with taxa reduced a priori to 81 were replicated with characters ordered or unordered as described in Ezcurra’s NEXUS file, as was a fourth unnumbered analysis of the Analysis 3 data matrix after Ezcurra’s a posteriori exclusion of *Kalisuchus rewanensis* and *Asperoris mnyama*. Ezcurra stated that his Analysis 3 was intended to allow subsequent workers to conduct studies without large amounts of computer memory and lengthy tree searches, but analysis of the 81 taxon data set produced >1,000 most parsimonious trees and its strict consensus tree included large polytomies (see discussion below), whereas the fourth analysis with taxa pruned to 79 produced only 24 most parsimonious trees and no large polytomies; therefore, the 79 taxon variant formed the basis for the subsequent analyses.

The data set of 600 characters was partitioned into four partitions reflecting body regions: Cranial, Postcranial, Forelimb, and Hindlimb partitions including 308 characters from the skull and mandible (Char. 1–308), 88 characters from the postcranial axial skeleton and osteoderms (Char. 309–383, 588–600), 71 characters from the pectoral girdle and forelimb Char. 384–454), and 133 characters from the pelvic girdle and hindlimb (Char. 545–587), respectively. The partitions were analyzed individually, and homogeneity testing was done on pair-wise comparisons of individual partitions and on each partition vs. all other characters, each based on 1,000 replicates. For comparison, Nesbitt’s (2011) data set of 412 characters was similarly partitioned into Cranial, Postcranial, Forelimb, and Hindlimb partitions including 176 characters from the skull and mandible (Char. 1–176), 47 characters from the postcranial axial skeleton and osteoderms (Char. 177–211, 401–412), 52 characters from the pectoral girdle and forelimb (Char. 212–263), and 137 characters from the pelvic girdle and hindlimb (Char. 264–400), respectively, and analyzed in the same way.

In order to examine the phylogenetic position of *Scleromochlus taylori*, it was coded for Ezcurra’s (2016) 600 characters based on the new information and interpretation in the present article, and was added to Ezcurra’s 79 taxon data matrix, resulting in an 80 taxon matrix (Supplemental Information). The 80 taxon data matrix was analyzed, with characters ordered or unordered as described in Ezcurra’s NEXUS file unless otherwise stated, to search for most parsimonious trees, strict consensus trees, and 50% majority-rule consensus trees. Two additional analyses were performed after altering the properties of two characters. For comparison, the coding of *Scleromochlus* in Bennett’s (2013) 19 taxon 134 character Updated Data Matrix was corrected on the basis of the new information and interpretation in the present article (Supplemental Information), and the matrix was similarly analyzed to search for most parsimonious trees, strict consensus trees, and 50% majority-rule consensus trees.

The remainder of this article consists of a Description section in which the six *Scleromochlus* specimens are described, a Discussion section in which information about the specimens is discussed and synthesized with subsections on the osteology and function, locomotion, and phylogenetic relationships of *Scleromochlus*, and a brief Conclusion.

## Description

Because the osteology of *Scleromochlus* has been described (Huene, 1914; Benton, 1999) and Benton (1999) presented diagrams of all six specimens, it is not necessary to present a general description of its osteology, and so only those features that are pertinent to the interpretation of *Scleromochlus’*s locomotion and phylogenetic relationships or that I think have been misinterpreted are described here.

### NHMUK R3146

The specimen consists of dorsal and ventral slabs that preserve two individuals lying side by side. That to the left is NHMUK R3146A, whereas that to the right and a little behind is NHMUK R3146B. Combining information from the dorsal and ventral slabs, NHMUK R3146A lies with its head turned slightly to the right, the trunk quite straight, and the tail bent slightly to the right (Fig. 3A). The humeri are directed laterally, the antebrachia directed anteriorly, the left hindlimb directed anterolaterally at ~26° with the knee fully flexed, and the right hindlimb with the femur directed laterally probably under NHMUK R3146B and the knee flexed at ~68°. NHMUK R3146B is somewhat smaller than NHMUK R3146A and lies with its head turned sharply to the left so that the snout lies over the right humerus of NHMUK R3146A and directly behind its head, the neck bent to the right, and the trunk quite straight. The humeri are directed posterolaterally and the right antebrachium directed anteriorly. The left femur is abducted ~68° and lying over the tail of NHMUK R3146A, the crus flexed to within ~30° of the femur, and the ankle fully flexed to lie along the crus. The right femur is directed laterally. Note that I disagree with Benton’s (1999: fig. 2b) interpretation of the left hindlimb of NHMUK R3146B on the ventral slab in that what he interpreted as the fibula is interpreted as the articulated tibia and fibula with a proximal tarsal articulated at the distal end and what he interpreted as the tibia is interpreted as the closely appressed metatarsals based on the morphology of the metatarsals and the lack of evidence of disturbance.

**Figure 4:**
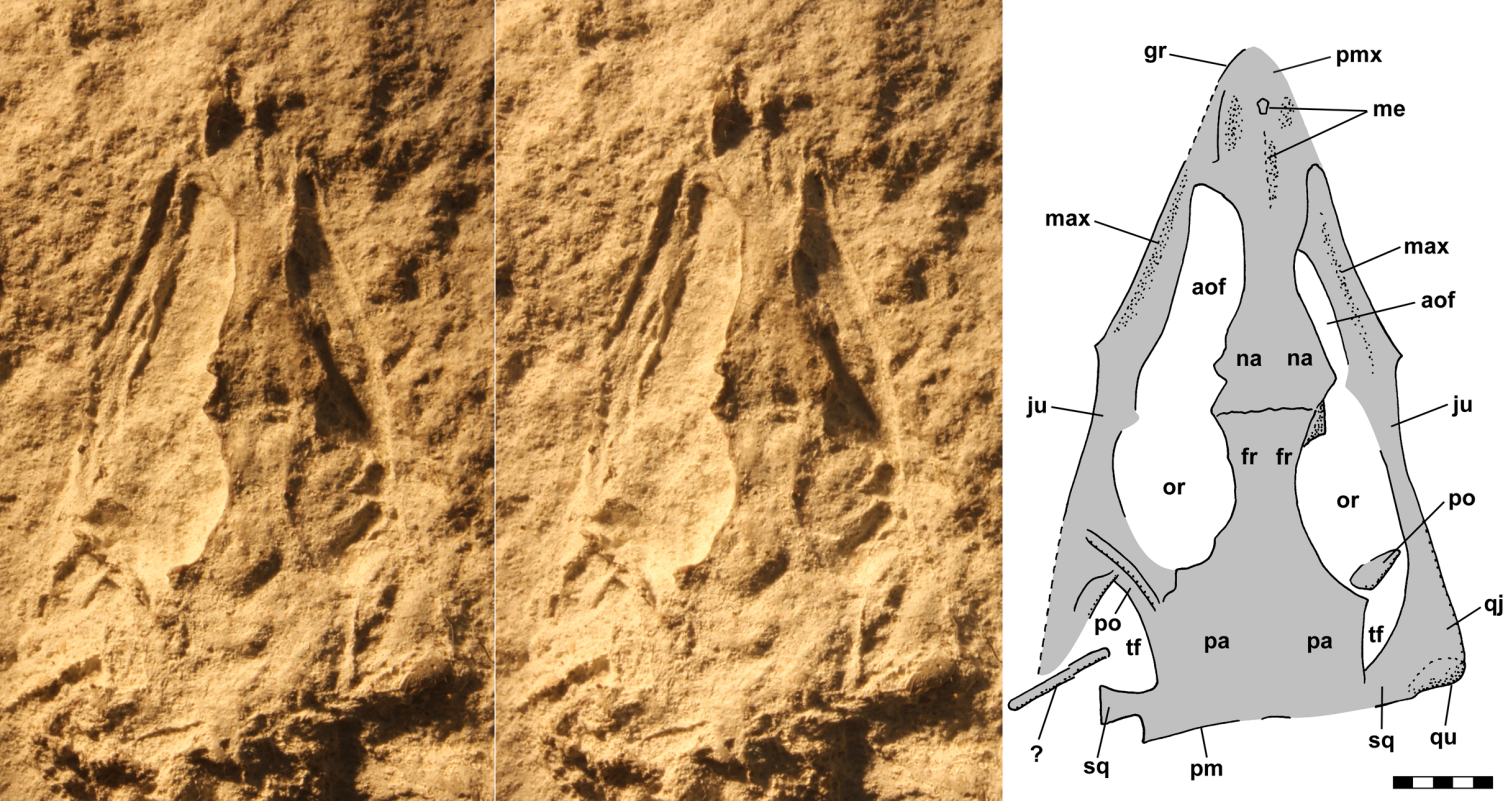
Stereo pair and interpretive drawing of skull of *Scleromochlus taylori*, NHMUK R3146A, in dorsal view on dorsal slab. Stereo pair of photographs and interpretive drawing of polyurethane rubber cast of the skull of *Scleromochlus taylori*, NHMUK R3146A, in dorsal view on dorsal slab. aof, antorbital fenestra; fr, frontal; gr, groove; ju, jugal; max, maxilla; me, median elements; na, nasal; or, orbit; pa, parietal; pm, posterior margin of parietal; pmx, premaxilla; po, postorbital; qj, quadratojugal; qu, quadrate; sq, squamosal; tf, temporal fenestra; and ?, indeterminate element. Scale bar = 5 mm.

The skull of NHMUK R3146A is well preserved on the dorsal slab and exhibits an overall triangular shape (Fig. 4). The upper jaw tip and narial region are poorly preserved, but a curving groove on the left suggests that the jaw tip was curved with a small radius. Traces of median elements suggest a septum between nasal capsules, see the shadows in Fig. 4, but there is no evidence of dorsal parts of the premaxillae or of external nares. Behind the narial region, the premaxilla and maxilla are preserved on the left, the maxilla on the right, the upper jaws diverging at ~45°, and between them narrow nasals and frontals are preserved slightly to the right of the midline. No sutures can be identified other than an angular depression that runs across the roofing elements a short distance behind the widest point between the antorbital fenestra and orbits, which probably represents the frontonasal sutures. The antorbital fenestrae are large and appear as if confluent with the orbits on both sides; however, that appearance is due to breakage of the prefrontals, lacrimals and jugals as the skull was crushed. There is a prominent ridge along the anterior margin of the antorbital fenestra, which is better preserved on the left. Sereno (1991) described the ridge as bounding a very large antorbital fossa and illustrated the contact of the premaxilla and maxilla as just anterior to the ridge. I can find no evidence of such a contact on either side and no evidence of antorbital fossae. Behind the orbits, the parietals are broad and seemingly flat, with a straight posterior margin nearly perpendicular to the midline present on the left. The lateral margins of the parietal region curve anterolaterally and presumably contact the postorbital, which bounds the upper and lower temporal fenestrae anteriorly as it passes down to contact the jugals.

The upper jaws formed of maxillae and jugals were L-shaped in cross-section, and their superior margins were rotated outward by the compression of the skull. As a consequence, what looks like the left lateral margin of the skull, gently concave lateral to the antorbital fenestrae, angular points where the preorbital bars would have been, and again concave lateral to the orbits, reflects the shape of the inferior margins of the antorbital fenestrae and the orbit, respectively, rather than the actual lateral margin of the skull, which was straight to gently convex. On the right, the lateral margin of the jugal continues posteriorly to a rounded posterior corner formed by the quadratojugal and quadrate, and a broad process passes dorsomedially from the corner. Medially, the right jugal and quadratojugal present what seems to be the curving posteroinferior margin of the lower temporal fenestra. On the left, an unidentified and seemingly displaced element lies at an oblique angle with its end in the temporal fenestra, and behind it the quadrate region is damaged. On the PVC cast the area behind the unidentified element is rather flat, whereas on the urethane cast and silicone rubber peel the area appears raised, presumably reflecting the loss of a chunk of matrix when the PVC cast was separated from the slab. The raised area does not seem to preserve bone traces except posteromedially where a portion of the squamosal, which is not preserved on the PVC cast, contacts the parietal. It presumably extended inferolaterally to the quadrate, but its lateral end exhibits breakage. The posterior margin is rather straight, whereas the anterior margin has a convex curve such that the squamosal widens toward the broken end. The curve presumably is the posterior margin of the upper temporal fenestra and its shape suggests that the break was close to the upper temporal bar separating the two temporal fenestrae.

**Figure 5:**
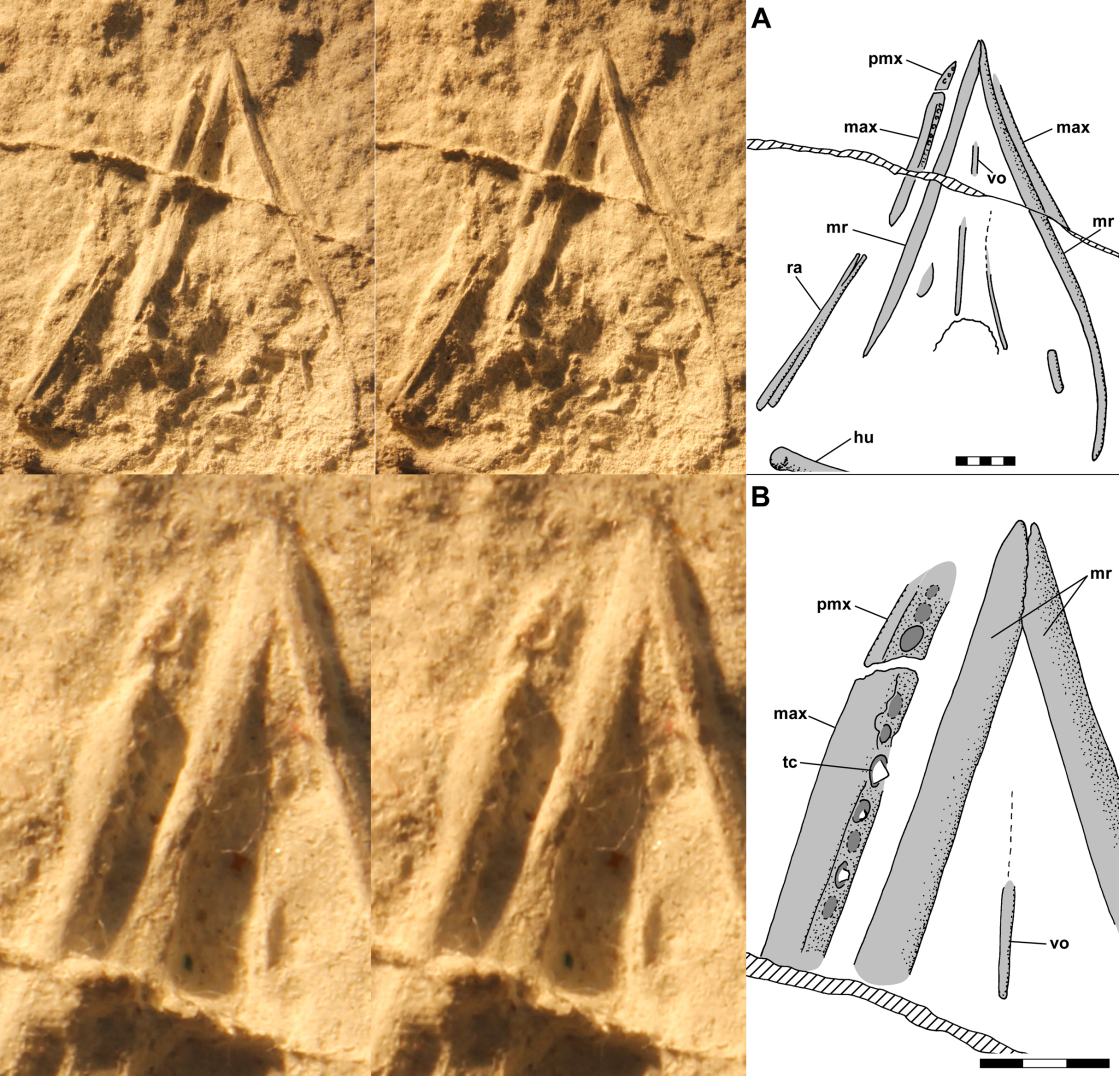
Stereo pairs and interpretive drawings of skull of NHMUK R3146A, in ventral view on ventral slab. Stereo pairs of photographs and interpretive drawings of polyurethane rubber cast of *Scleromochlus taylori*, NHMUK R3146A, in ventral view on ventral slab. (A) Skull. (B) Detail of mandibular tip and teeth. The irregular ridge (hatched) running obliquely across the cast is a crack in the slab. max, maxilla; hu, humerus; mr, mandibular ramus; pmx, premaxilla; ra, radius; tc, tooth crown; and vo, vomer. Scale bars = 5 and 3 mm.

On the ventral slab, the skull of NHMUK R3146A preserves the right premaxilla and maxilla, left maxilla, both mandibular rami in articulation, and a few isolated elements that probably pertain to the palate (Fig. 5). The right premaxilla and maxilla are separated by a slight gap that presumably represents a disarticulation, and the dentigerous surface of the maxilla, which is rather flat and meets the lateral surface at ~90°, is rotated inward ~60°, which agrees with the outward rotation noted on the dorsal slab, whereas the premaxilla exhibits much less rotation. The left maxilla is also rotated inward, and it lies above the mandibular ramus such that the ramus obscures the dentigerous surface of the left maxilla. Of the palatal elements, only a vomer close to the midline can be identified with any confidence. Benton (1999) identified one element as a hyoid, but I can find no evidence to support that interpretation.

The preserved portion of the right premaxilla bears three suboval alveoli, the last ~75% longer than wide, but there may have been additional teeth anterior on the missing portion. The maxilla bears seven alveoli with a tooth in the third alveolus and traces of teeth in the fourth and sixth (Fig. 5B). The third tooth is preserved in ventrolateral view because of the rotation of the maxilla. The crown is low and subconical with the anterior and posterior surfaces meeting at a ~90° angle, whereas the medial and lateral surfaces would have met at an acute angle. There might have been a diastema between the premaxillary and maxillary teeth, but there is no clear evidence that there was.

The mandible lies between the upper jaws with its rami parallel to them. The rami are slender, their medial surfaces appear flat, and the lateral surfaces convex and curving medially to meet the medial surface in a sharp-edged ventral margin. The rami meet in a short pointed symphysis in which they are closely appressed but seem not to be fused, and they diverge at ~41°. The symphyseal region tapers upward in lateral view to a pointed tip with a longer and shallower taper than in Benton’s (1999; Fig. 2H) lateral view. The right ramus is almost straight, but is missing its posterior third. The left appears to be complete, and its anterior part exhibits a slightly concave lateral curvature before curving to a slightly inward-pointed retroarticular process. Given the straight upper jaws and right ramus, the concave curvature of the left ramus is probably an artifact of compression. Note that the left ramus is in contact with the left maxilla laterally, whereas there is a ~1 mm gap between the right ramus and right maxilla. That probably reflects slight displacement of the mandible to the left during compression, lateral displacement of the left maxilla and jugal during their rotation, or both.

**Figure 6:**
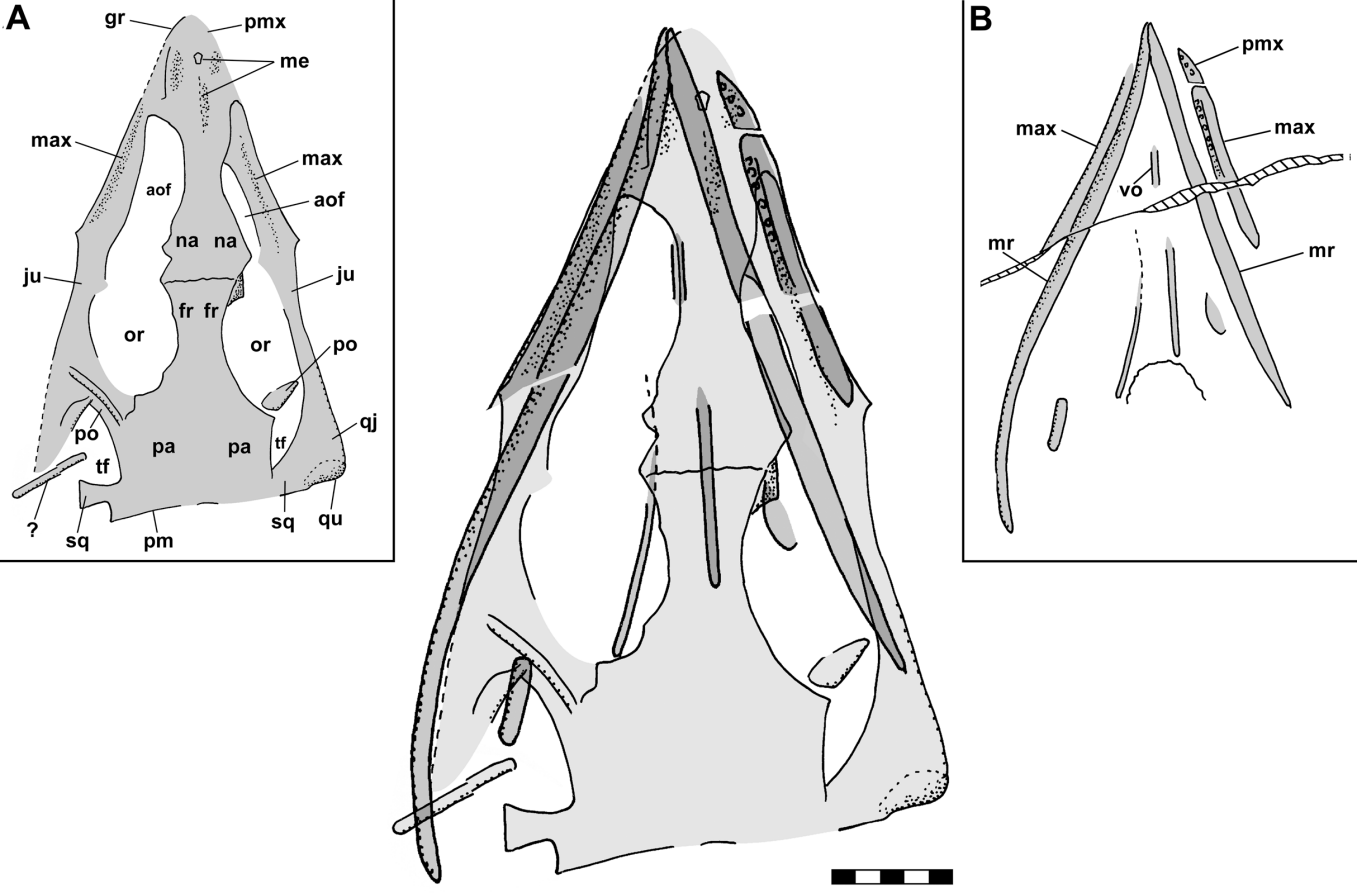
Composite interpretive drawing of the skull of *Scleromochlus taylori*, NHMUK R3146A, in dorsal view combining information from polyurethane rubber casts of the dorsal and ventral slabs. Elements on the dorsal slab indicated with light gray, elements on the ventral slab indicated with medium gray, and overlapping elements indicated with dark gray. Insets (A) and (B) show the drawings of the dorsal slab and ventral slab (flipped horizontally) to aid in interpretation. aof, antorbital fenestra; fr, frontal; gr, groove; ju, jugal; max, maxilla; me, median elements; mr, mandibular ramus; na, nasal; or, orbit; pa, parietal; pm, posterior margin of parietal; pmx, premaxilla; po, postorbital; qj, quadratojugal; qu, quadrate; sq, squamosal; tc, tooth crown; tf, temporal fenestra; vo, vomer; and ?, indeterminate element. Scale bar = 5 mm.

Benton (1999) interpreted the left maxilla and jugal of NHMUK R3146A on the dorsal slab as the left dentary and splenial and the left maxilla on the ventral slab as the dentary, and Benton & Walker (1985) and Benton (1999) interpreted the left ramus as having a long retroarticular process. However, combining information from the dorsal and ventral slabs (Fig. 6) confirms the identifications of the left maxilla and shows that the quadrate would have been close to the posterior end of the mandibular ramus, supporting the present interpretation of a short retroarticular process.

The dorsal slab of NHMUK R3146A preserves an articulated series of anterior vertebrae and eight gently curving ribs on the left, and articulated dorsal osteoderms over the posterior trunk. The first rib is adjacent to the anterior end of the scapula, shorter than the subsequent ribs, and probably was too short to have articulated with the sternum, and on that basis the first rib is interpreted as the last cervical rib and the vertebrae that supports it, which preserves an anteroposteriorly long neural spine, is interpreted as the last cervical. It is not possible to identify or measure other cervical vertebrae, but the distance from the presumed posterior skull margin to the cervico-dorsal transition is 12.3 mm. That would be equivalent to five 2.4 mm or six 2 mm long vertebral segments, and thus six or seven cervicals if the atlas was of only negligible length. NHMUK R3146A does not exhibit articulated anterior dorsal centra, but the length from the last cervical rib to 7th dorsal rib on the articulated left side of the ribcage on the dorsal slab, measured as far medially as possible, is 19.6 mm, which corresponds to a length of 2.8 mm/segment. The distance from the cervico-dorsal transition to the anterior end of the sacrum is 49.0 mm. There are three sacral vertebrae bounded laterally by the right ilium, and their combined length is 6.8 mm (2.3 mm/segment). It is not clear that either the immediately preceding or subsequent vertebrae articulated with the ilia such that they could be considered sacral vertebrae.

The dorsal ribs extend up to 9.4 mm from the midline, and there is a shallow curving groove ~1 mm lateral to them that may represent the lateral margin of the body. The ribs are gently curved posteriorly in dorsal view, most with a radius of ~17 mm though an anterior one has a radius of ~10 mm, and the distance between adjacent ribs increases laterally. The lateral ends of the ribs provide no evidence of terminations and the ribs probably extended some distance down into the ventral slab.

The posterior trunk of NHMUK R3146A on the dorsal slab preserves a large area with transverse striations visible under low angle illumination. The anteriormost striation is a short distance behind the 7th dorsal rib and 21.6 mm anterior to the first sacral vertebra, but the width of the area is difficult to determine. Here the striations are interpreted as representing dorsal osteoderms (see “Discussion”). Benton (1999: fig. 2a) illustrated the osteoderms with transverse lines medially and posterolaterally slanting lines laterally, perhaps implying that he thought there were medial and lateral elements, with the lateral elements presumably covering the lateral surfaces of a narrow trunk. Woodward (1907) did not illustrate such angled striations, and I can find neither posterolaterally angled striations lateral to the transverse striations nor any evidence of a division into medial and lateral elements. Note that there is a series of five closely spaced anterolaterally slanting structures on the right at the anterior end of the osteoderms (Benton, 1999: fig. 2a illustrated three of them). The structures appear cylindrical and their spacing is less than the anteroposterior length of osteoderm segments, which along with their anterolaterally slanting orientation suggest that the structures are not lateral osteoderm elements. It is not clear what the structures are.

The humeri of NHMUK R3146A are directed laterally with their heads 11.6 mm apart. On the ventral slab, a displaced cervical vertebra in anterior view with low neural arch and robust zygapophyses lies just medial to the head of the left humerus. Benton (1999: fig. 2b) illustrated the cervical and humerus drawn as if a single element. On the dorsal slab, the left scapula lies parallel to the vertebral column and ~4 mm lateral to it, and curves medially toward its anterior end. The close spacing of the shoulder joints suggests postmortem displacement during compression of the specimen.

The skull of NHMUK R3146B agrees with that of NHMUK R3146A in many details, but does not provide much additional information. On the dorsal slab, the left posterior corner of the skull presents an anterolaterally slanting lateral margin, which may have been the basis of the slanting lateral margin in Benton’s (1999: fig. 8a; Fig. 2F) skull reconstruction. However, I can find no evidence to suggest that the slanting margin is not fractured and reflects the condition in life. On the ventral slab, the mandibular rami are preserved with maxillae and jugals lying just lateral to them as in NHMUK R3146A. Benton (1999: fig. 9d) illustrated the posterior part of the right ramus as significantly deeper than the anterior part, but the specimen preserves only the ventral margin of the posterior part of the ramus and provides no information as to its depth.

The distance from the presumed posterior skull margin of NHMUK R3146B on the dorsal slab to the cervico-dorsal transition is ~15 mm and the combined length of three posterior cervical centra on the ventral slab, identified on the basis of their position relative to the proximal humeri, is 6.35 mm (2.1 mm/segment). This suggests there were seven cervical vertebrae. The combined length of three sacrals on the dorsal slab is 6.4 mm (2.1 mm/segment), and that of the subsequent four caudals on the dorsal slab is 8.2 mm (2.1 mm/segment). Note that the right ilium of NHMUK R3146B on the dorsal slab is ~6.8 mm long, though the posterior end may be missing. The distance from the cervico-dorsal transition to the anterior end of the sacrum on the dorsal slab is 40.7 mm, thus ~83% the size of NHMUK R3146A.

**Figure 7:**
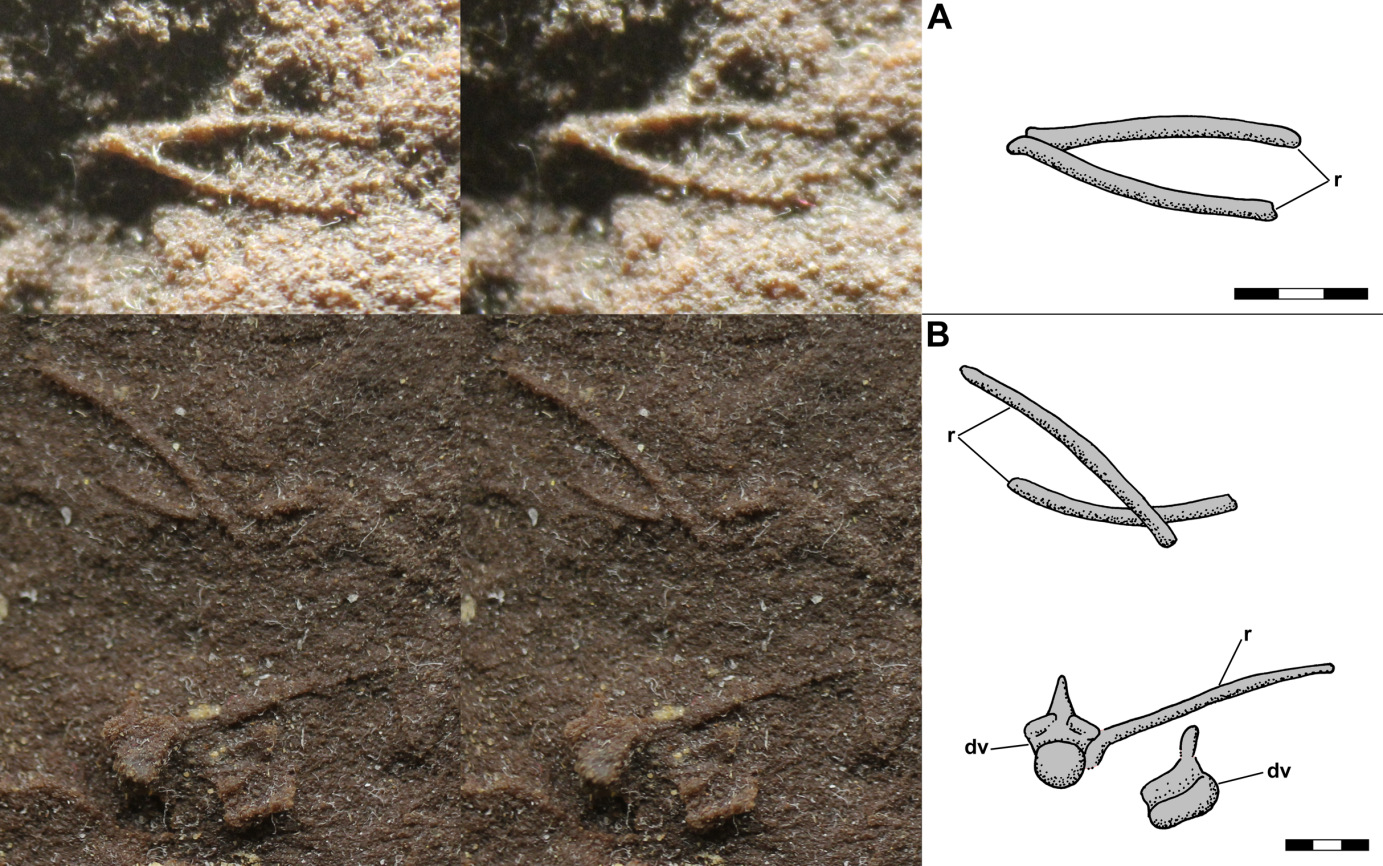
Stereo pairs and interpretive drawings of *Scleromochlus taylori ribs*. Stereo pairs of photographs and interpretive drawings of *Scleromochlus taylori*. (A) Fortuitous alignment of dorsal ribs that resembles a chevron on right side of NHMUK R3146B on polyurethane rubber cast of dorsal slab. (B) Overlapping dorsal ribs and articulated dorsal vertebra and right rib of NHMUK R5589 in posterior view on PVC cast of dorsal slab. dv, dorsal vertebra; r, dorsal rib. Scale bars = 3 mm.

Benton (1999: fig. 2a) illustrated a feature of NHMUK R3146B to the right of the mid-dorsal vertebrae on the dorsal slab and interpreted it as a chevron. The feature is 5.6 mm long, 2.1 mm wide, and appears symmetrical. Although the apparent symmetry suggests the feature is a median element, it is much larger than the chevrons of NHMUK R3557 illustrated by Woodward (1907) and Huene (1914), which are slightly shorter than, but equivalent in width to, the caudal vertebrae with which they articulated. In addition, it would be unusual to find a chevron preserved in the mid-trunk region of NHMUK R3146B, which exhibits no significant disturbance. Close examination of the leftmost end of the feature reveals that it is a fortuitous alignment of two gently curving dorsal rib segments with the end of the posterior one lying atop the end of the anterior one (Fig. 7A). The radius of the ribs’ curvature is ~17.5 mm.

### NHMUK R3556

The specimen consists of dorsal and ventral slabs that preserve one individual. Combining their information, the head, neck, and anterior trunk skeleton are bent strongly to the left so that the skull lies near the left knee (Fig. 3D). The posterior part of the trunk skeleton is straight, and the tail is bent slightly to the right. The right forelimb is directed posteriorly along the body with the antebrachium fully flexed against the humerus. The left forelimb is not visible. The right hindlimb is directed anteriorly along the body with the knee and ankle fully flexed, and the left hindlimb is directed anterolaterally at ~43° with the crus flexed to within ~14° of the femur and the ankle flexed to within ~29° of the crus.

**Figure 8:**
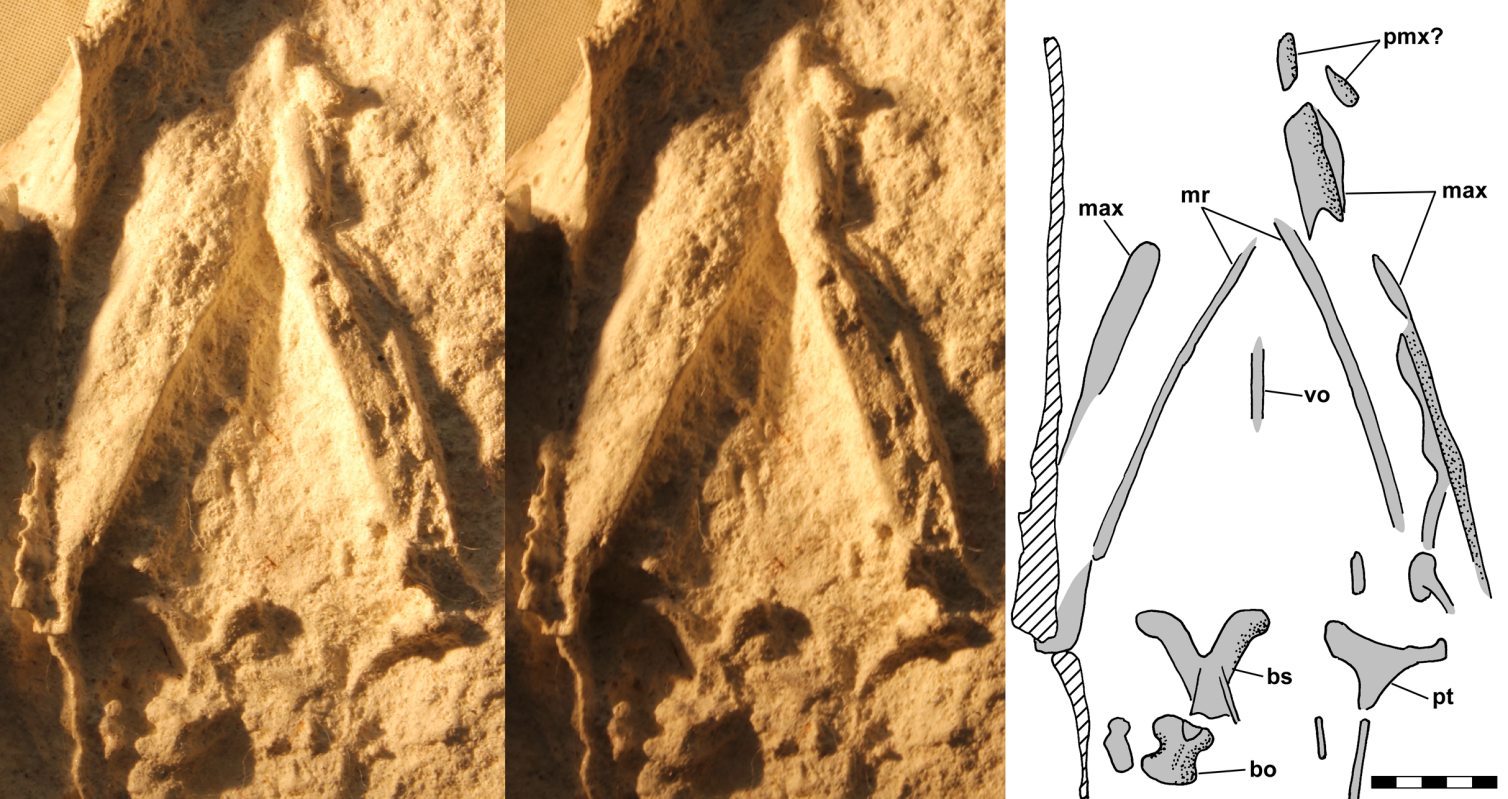
Stereo pair and interpretive drawing of polyurethane rubber cast of skull and mandible of *Scleromochlus taylori*, NHMUK R3556, in ventral view on ventral slab. Stereo pair of photographs and interpretive drawing of polyurethane rubber cast of skull and mandible of *Scleromochlus taylori*, NHMUK R3556, in ventral view on ventral slab. The irregular sharp ridge (hatched) at far left is a crack in the slab. Lighting is from the left rather than the usual upper left so as to adequately illuminate the right side ramus and palate. bo, basioccipital; bs, basisphenoid; max, maxilla; pmx?, premaxilla?; pt, pterygoid; and v, vomer. Scale bar = 5 mm.

The skull is poorly preserved in dorsal view on the dorsal slab. The jaw tip and narial region are missing and the posterior margin of the skull roof is indistinct, but the antorbital fenestrae and orbits flank the narrow nasals and frontals and the left jaw margin is straight anteriorly and curving posteriorly. The ventral slab preserves parts of the upper and lower jaws and a few palatal and braincase elements (Fig. 8). Anteriorly there are two fragments that may pertain to the premaxillae, and behind them there are parts of the maxillae and perhaps jugals preserved on the left and right that diverge at ~40°. The ventral parts of the mandibular rami are preserved ~2.5 mm medial to the upper jaw elements. Anteriorly, the rami diverge at ~55° and both are preserved well medial to the upper jaw elements and exhibit convex outward curves, which suggests postmortem distortion. As such, the specimen provides little useful information beyond the angle and shape of the lower jaw. When the slabs were split, the matrix broke away between the maxillae, jugals and mandibular rami, resulting on each side in a broad rather flat surface bounded by upper and lower jaw elements and raised above the level of the surrounding matrix. Benton (1999: fig. 10b) interpreted the combinations of upper and lower jaw margins and flat intervening matrix as the lateral surfaces of the mandibular rami. I accepted Benton’s (1999) interpretation of the jaws for several years until questions as to why the rami would be so deep, would have been rotated to expose their lateral surfaces, and would have had such irregular texture on their lateral surfaces led to a reassessment and the present interpretation. Note that Benton (1999: figs. 3b and 10b) interpreted the right mandibular as extended posteriorly so as to form a long retromandibular process; however, Fig. 7 clearly shows that the supposed feature is a crack in the slab.

A small nearly median element between the mandibular rami is interpreted as a vomer, and posteriorly on the left is a triradiate element with a slender ramus extending laterally and a more robust ramus extending medially, which is interpreted as a pterygoid. A robust symmetrical Y-shaped element is preserved slightly to the right of the midline, and behind it and seemingly disarticulated from it is a smaller symmetrical element. Benton (1999) identified the two elements as basisphenoid and basioccipital, respectively, and I concur.

The ventral slab preserves a markedly curved articulated series of seven vertebral centra, the anterior end of which lies immediately behind, and thus essentially in articulation with, the basioccipital. Benton (1999) illustrated a small spherical element between the basioccipital and the first full-sized cervical vertebra, and considered it to be the atlas and the first full-sized cervical to be the axis. I question the identity of the small element, but the first full-sized cervical vertebra behind the basioccipital is too large to be the atlas alone and so is interpreted as either an atlas-axis complex or the axis alone. The curving series of C2–C6 has a midline radius of 12.4 mm, a chord length of 10.4 mm, and an arc of 55°, suggesting that the length of the series when straight would have been 11.1 mm (2.2 mm/segment). However, the vertebrae appear amphiplatyan and the gaps between adjacent vertebrae appear slightly larger than they would have when straight, so those calculations may overestimate the cervical vertebral segment length slightly. The last of the seven vertebrae is flanked laterally by the right coracoid on the ventral slab and by the anterior end of the left scapula on the dorsal slab, and on that basis that vertebra is considered to be the first dorsal.

The dorsal slab preserves a markedly curved series of neural spines corresponding to the cervical centra on the ventral slab. The anteriormost neural spine is longer anteroposteriorly than the subsequent ones, and combining information from the dorsal and ventral slabs (see Benton, 1999: figs. 3a and 3b) shows it to be the axis. Its position relative to the posterior margins of the left orbit and the skull roof shows that the basioccipital did not extend behind the posterior margin of the skull roof.

The dorsal slab preserves a series of articulated dorsals, sacrals, and subsequent caudal vertebrae. The combined length of the last four dorsal vertebrae anterior to the crack dividing the slab is 9.1 mm (2.3 mm/segment), that of three sacrals flanked laterally by both ilia is 6.5 mm (2.2 mm/segment), and that of the 10 subsequent caudals is 25.9 mm (2.6 mm/segment). Note that Benton’s (1999: fig. 3a) figure seems to show 11 caudal vertebrae, but I find only 10. The right ilium is preserved with its anterior process rotated upward, has a preserved length of 8.4 mm, and seems to be complete. The left ilium has a preserved length of 7.8 mm but its posterior process disappears into the matrix so it probably was longer. Benton (1999: fig. 3b) stated that the length of the ilium was 10 mm, but illustrated the right ilium as ~8.5 mm and the left ilium even shorter. Given the positions of the ilia relative to the vertebral centra and assuming that sacrals would be neither anterior nor posterior to the ilia suggests that there were only three sacral vertebrae. The transverse processes of the fourth vertebra posterior to the dorso-sacral transition would have had to angle forward to fully contact the ilium, which seems unlikely, and on that basis that vertebra is considered to be the first caudal. The combined length of the four posterior dorsal vertebrae posterior to the crack on the ventral slab is 10.7 mm (~2.7 mm/segment).

**Figure 9:**
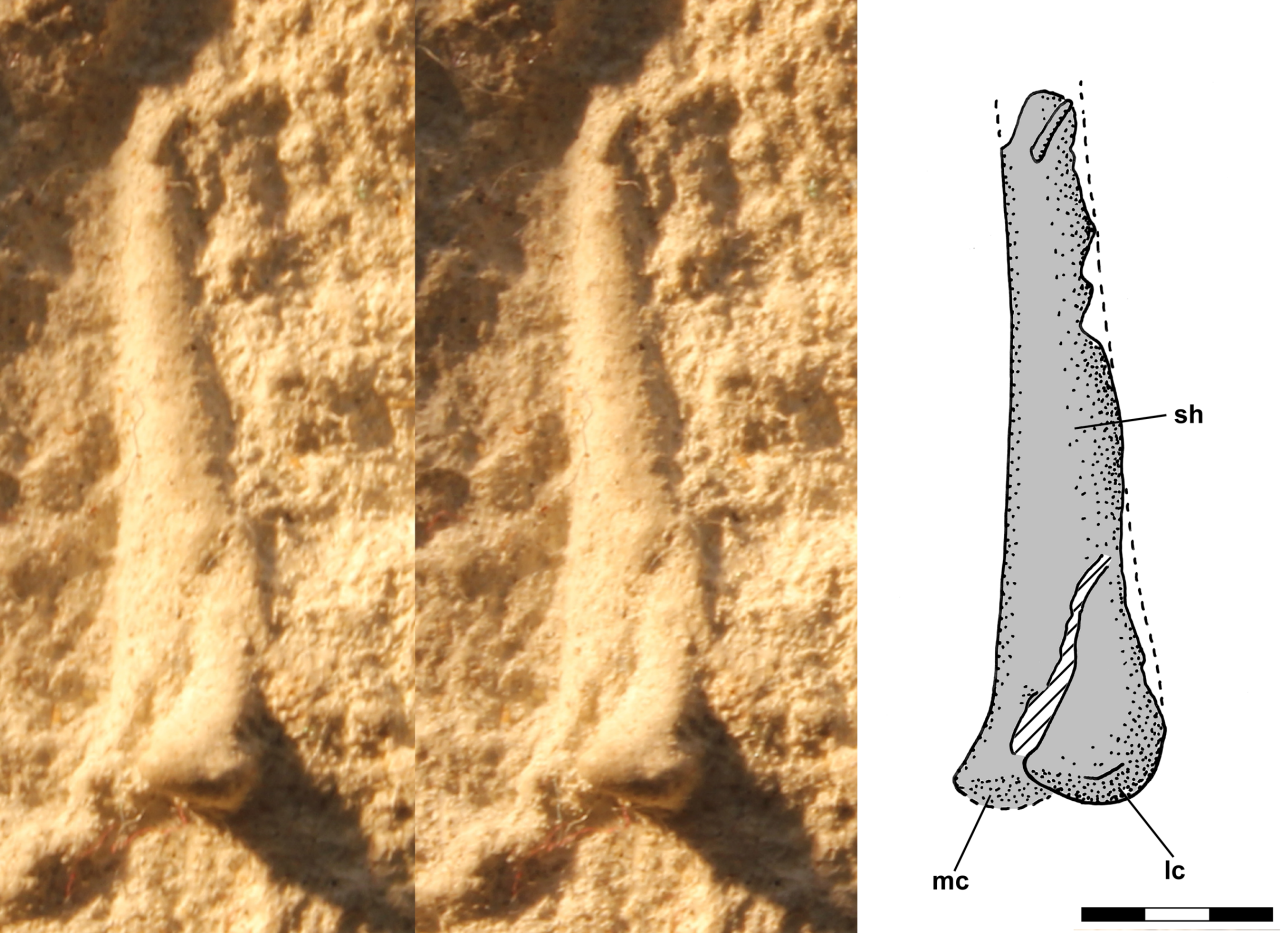
Stereo pair and interpretive drawing of polyurethane rubber cast of distal right femur of *Scleromochlus taylori*, NHMUK R3556, in oblique posterolateral view on ventral slab. The hatched feature is interpreted as a fracture in the shaft. lc, lateral condyle; mc, medial condyle; and sh, shaft. Scale bar = 3 mm.

The distal end of the right femur is exposed in oblique posterolateral view on the ventral slab with a small oblique fracture running through the impression (Fig. 9). The lateral condyle is large and extends behind and lateral to the shaft and there is a large intercondylar fossa, but little of the medial condyle is visible.

**Figure 10:**
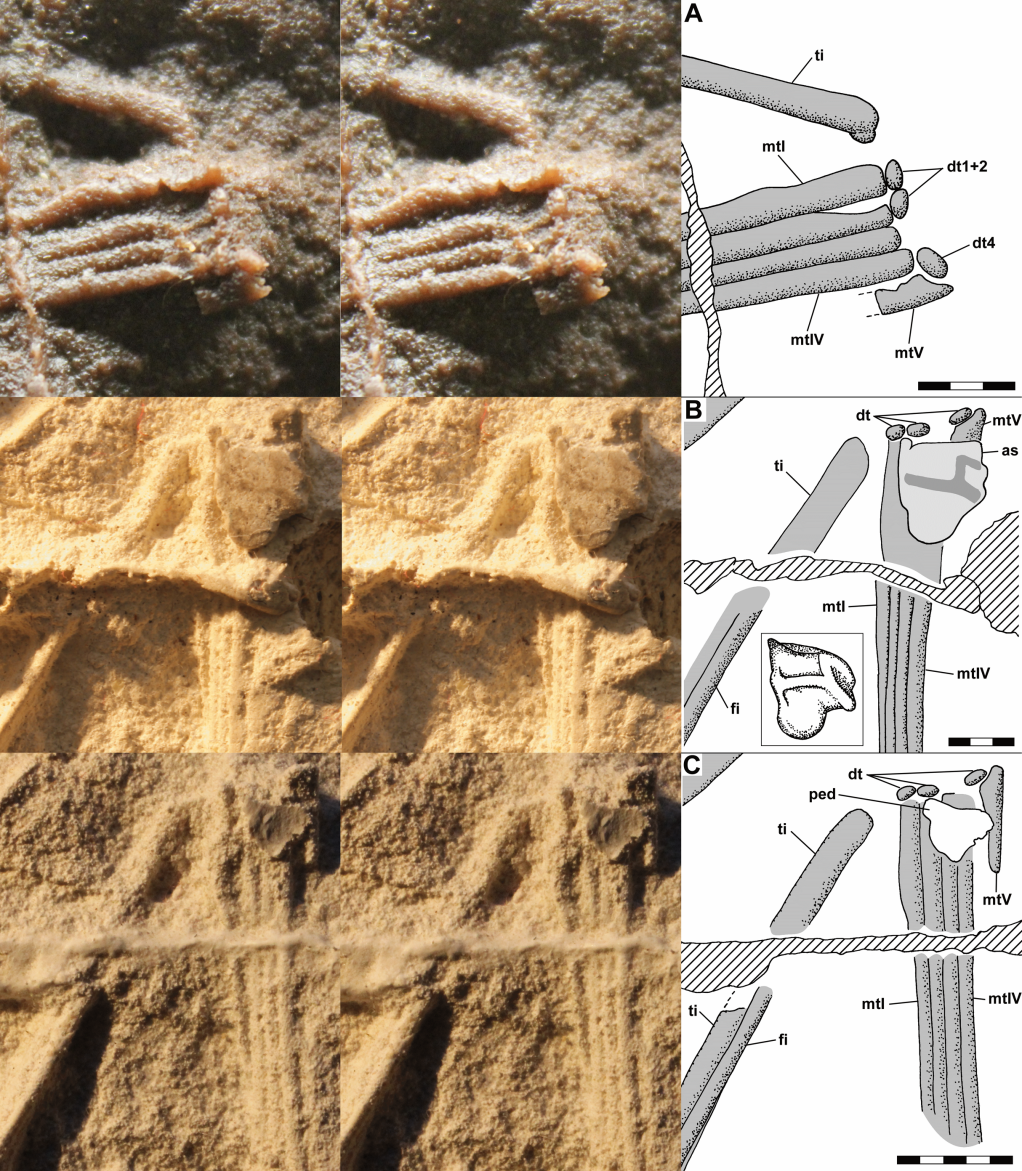
Stereo pairs of photographs and interpretive drawings of left tarsus and metatarsus of *Scleromochlus taylori*, NHMUK R3556, on dorsal slab. (A) PVC cast showing distal tarsals 1, 2 and 4 and incomplete metatarsal V. (B) Polyurethane rubber cast (rotated ~90° counterclockwise relative to (A) showing astragalus flattened by pressure covering proximal ends of metatarsals. (C) Silicone rubber peel showing distal tarsals, surface where astragalus ripped off, and complete metatarsal V. Inset in (B) shows astragalus, redrawn and rotated from Padian (1984: fig. 3b). The hatched prominent vertical feature at left in (A) and horizontal feature in (B) and (C) is a crack in the slab. Note that (B) and (C) present metatarsals I–IV in oblique view. as, astragalus; dt, distal tarsal; fi, fibula; mt, metatarsal; ped, pedestal that supported astragalus; and ti, tibia. Scale bars = 3 and 5 mm.

The dorsal slab preserves the left crus, tarsus, and metatarsus in close association. The slab is broken, and the resulting crack produces a prominent ridge running across the casts; vertically at left in Fig. 9A and horizontally across the crus and metatarsus in Figs. 9B and 9C. The tibia and fibula are preserved in articulation, and the midshaft diameter of the tibia measured on a photograph is 1.75 mm, whereas that of the fibula is 0.80 mm (Fig. 10C). On the PVC cast, small ovoid distal tarsals 1, 2 and 4 are preserved in articulation with the proximal ends of metatarsals (Mt) I, II and IV and V (Fig. 10A). Benton (1999) described distal tarsals 1–3 as cuboidal and distal tarsal 4 as small, implying that distal tarsal 3 was visible and that distal tarsal 4 was smaller than the first three. However, there is a gap between distal tarsals 2 and 4 where distal tarsal 3 presumably had been, and distal tarsal 4 is slightly larger than 1 and 2, presumably because it articulated with both Mt IV and V. Whereas I accept that *Scleromochlus* had distal tarsals 1–4, the gap exhibits only an irregularly textured surface that differs from that of the matrix and presumably reflects a mulmy infilling of the negative impression of distal tarsal 3. The metatarsus consists of elongate Mt 1–4 with the proximal ends in contact with one another, the shafts cylindrical and closely appressed but without any sign of fusion, and a much shorter Mt V, slightly separated from Mt 1–4, but not divergent. A clean break across Mt V on the PVC cast indicates that the bone continued distally for some distance.

The polyurethane cast of the dorsal slab records a misalignment of the two halves of the slab such that the half with the tarsus and proximal metatarsus is displaced upward and to the right relative to the half with the distal metatarsus. In addition, the former half seems to have been damaged by previous (PVC?) casting such that the distal tibia and proximal metatarsus are poorly preserved, whereas the latter half still preserves the distal metatarsus well. The cast preserves a large subtriangular astragalus, 4.7 × 4.0 mm, atop the proximal end of the metatarsus (Fig. 10B). Padian (1984: fig. 3b) illustrated the astragalus (identified as a medial distal tarsal) with an h-shaped pattern of ridges on its upper surface; however, when I examined the cast pressure had obliterated the ridges though the smoothed surface exhibited a roughly h-shaped pattern of dark lines, probably dirt collected by the ridges before they were flattened. It is not clear whether pressure increased the length and width of the astragalus; Padian’s (1984: fig. 3) figure shows it as 4.0 × 3.2 mm, 15–20% smaller than my measurements, but the scale bar may be incorrect because the figure also shows the calcaneum of NHMUK R3557 as 3.2 × 2.0 mm, ~10% smaller than my measurements. The appearance of the astragalus on the polyurethane cast where there had been none on the PVC cast presumably resulted from the rupture of a thin sheet of matrix between the impressions of the metatarsals and the cavity where the astragalus had been, which permitted the polyurethane to take a positive impression of the cavity. Distal tarsals 1 and 4 and the proximal end of Mt V are also visible on the polyurethane cast, though they are not as distinct as on the PVC cast.

The silicone rubber peel of the dorsal slab presumably also had had the astragalus atop the proximal metatarsus, but when I examined it there was only the pedestal that had supported the positive impression of the astragalus before it was torn off (presumably to remain in the impression), and which recorded the size of the hole into the astragalus’s negative impression (Fig. 10C). Distal tarsals 1, 2 and 4 are visible and the Mt V is complete, 4.5 mm long, and shows no sign of having supported phalanges.

The dorsal slab of NHMUK R3556 also preserves the right metatarsus, and on the silicone rubber peel distal tarsal 1 is preserved at the proximal end of Mt IV, whereas it is not present on the PVC cast. The appearance of the distal tarsal on the silicone rubber peel where there had been none on the PVC cast was presumably the result of removal of a mulmy infilling of the negative impression of the tarsal or the rupture of a thin sheet of matrix between the impressions of the metatarsals and the cavity where the distal tarsal had been, which permitted the silicone rubber to take a positive impression of the cavity.

### NHMUK R3557

The specimen consists of dorsal and ventral slabs that preserve one individual. Combining their information, the skeleton lies with its head turned slightly to the left, its trunk quite straight and its tail bent slightly to the left before turning sharply to the right (Fig. 3B). The right humerus is directed anterolaterally and the right hindlimb directed anteriorly along the body with the crus flexed to within ~13° of the femur and the ankle somewhat extended. There has been significant disturbance to the left side such that a section of dorsal osteoderms has been displaced, the left forelimb is not visible, and the left pelvic girdle and hindlimb are displaced and somewhat jumbled though the hindlimb probably had been folded compactly much as the right.

The dorsal slab preserves parts of the upper jaws and anterior skull roof, but the jaw tip and narial region and the quadrate and parietal regions are missing. Benton (1999: fig. 10c) illustrated antorbital fossae on the specimen, but I can find no evidence of them. The ventral slab preserves upper and lower jaw elements, but adds little information. The right maxilla preserves ~8 teeth, but they are not as clear as those of NHMUK R3146A. They extend further above the dentigerous surface of the maxilla than the best preserved tooth of NHMUK R3146A, suggesting that they were fully erupted, but do not support the interpretation of teeth as long as those in the skull reconstructions of Huene (1914), Sereno (1991) and Benton (1999). Benton (1999: figs. 4b and 10d) labeled the same element as the right dentary and splenial in two different figures, and interpreted the right ramus of the mandible preserved on the ventral slab as exhibiting evidence of a mandibular fenestra. However, I can find no evidence of finished bone surfaces and consider the gap that Benton interpreted as a fenestra to be an artifact of fracturing of mandibular elements during splitting of the slabs.

A series of seven mid-dorsal vertebrae adjacent to the femur on the ventral slab exhibits variation in centrum length. The combined length of the longest three vertebrae in the middle of the series is 10.6 mm (3.5 mm/segment).

The ventral slab preserves a large area of articulated dorsal osteoderms, ~13 × 7.5 mm, displaced and preserved anterior to the left knee within the disturbed area. The fact that the osteoderms are preserved in articulation with one another despite having been displaced suggests that they were firmly connected to one another laterally and longitudinally.

**Figure 11:**
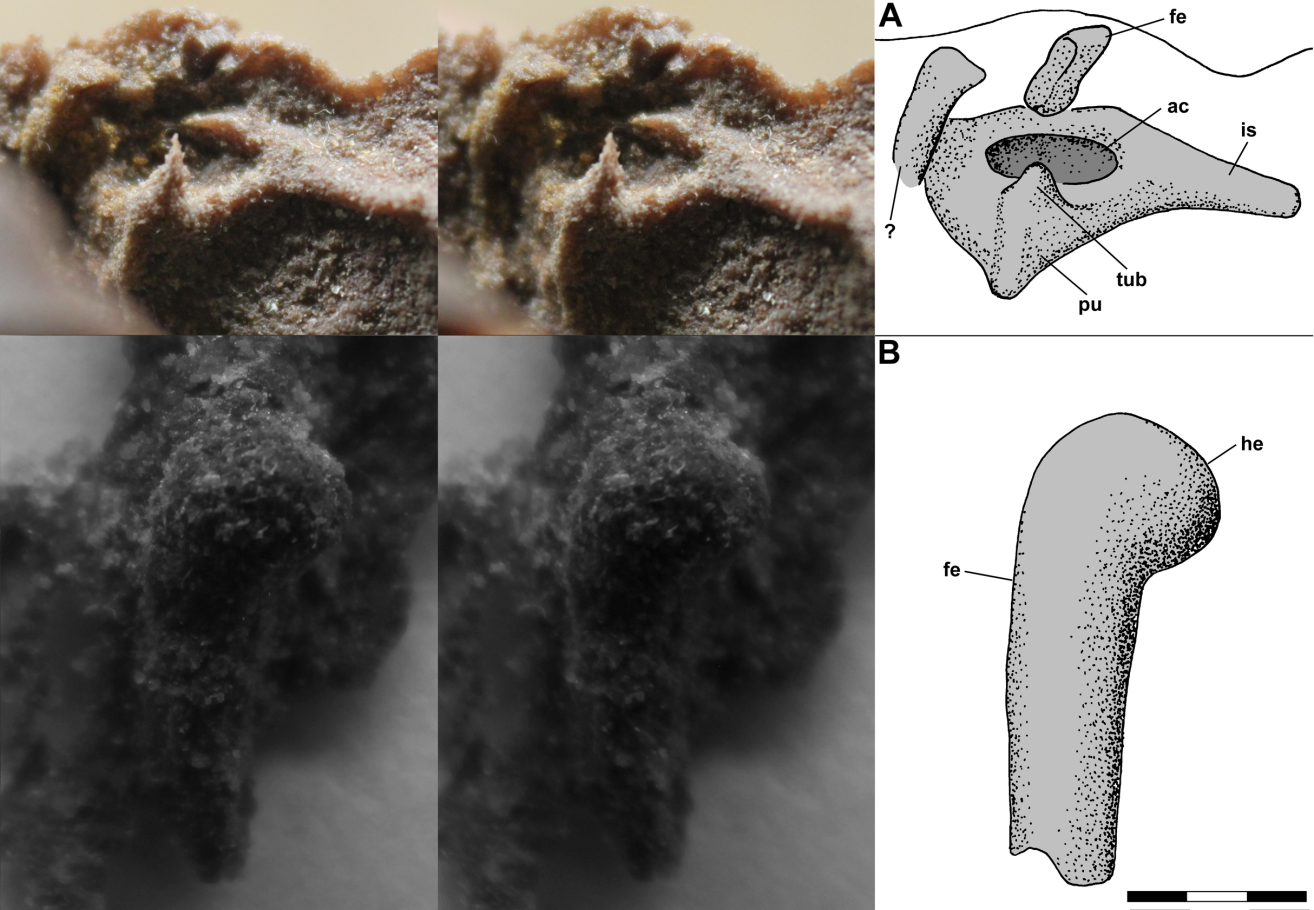
Stereo pairs of photographs and interpretive drawings of PVC casts of *Scleromochlus taylori*, NHMUK R3557. (A) Left pelvic girdle and acetabulum in oblique lateral view on side of dorsal slab. (B) Left proximal femur in oblique posterodorsal? view. ac, acetabulum; fe, femur; he, head; is, ischium; pu, pubis; tub, tubercle; and ?, indeterminate element. Scale bar = 3 mm.

The left pelvic girdle is exposed on the side of the dorsal slab, and a PVC cast of the region exhibits a weakly triradiate structure with a shallow pubis and elongate ischium and an imperforate acetabulum (Fig. 11A). There is a prominent tubercle on the pubis below the acetabulum with an irregular spike, which seems to be an artifact, atop it. The tubercle, which Benton (1999: fig. 13a) described as a substantial medial process, has a rounded surface and presumably was associated with the origin(s) of *m. pubotibialis* and/or *m. puboischiotibialis*. Above the ilium, the shaft of the femur, flattened presumably by pressure, extends a short distance out of the matrix. Note that Benton (1999: fig. 4a) drew the left femur on the dorsal slab as having a markedly bowed shaft; however, the shaft has only a slight curvature. A small isolated PVC cast of the proximal femur shows that the head was hemispherical and weakly inflected with the articular surface covering much of the proximal end (Fig. 11B).

Benton (1999) identified the right ischium and pubis and sacral vertebrae on the ventral slab, and I accept those interpretations. Note that the pubis is what Sereno (1991) interpreted as the prominent inturned head of the right femur; however, the proximal third of the femoral shaft is not visible on the ventral slab and the pubis has no connection with the preserved shaft.

**Figure 12:**
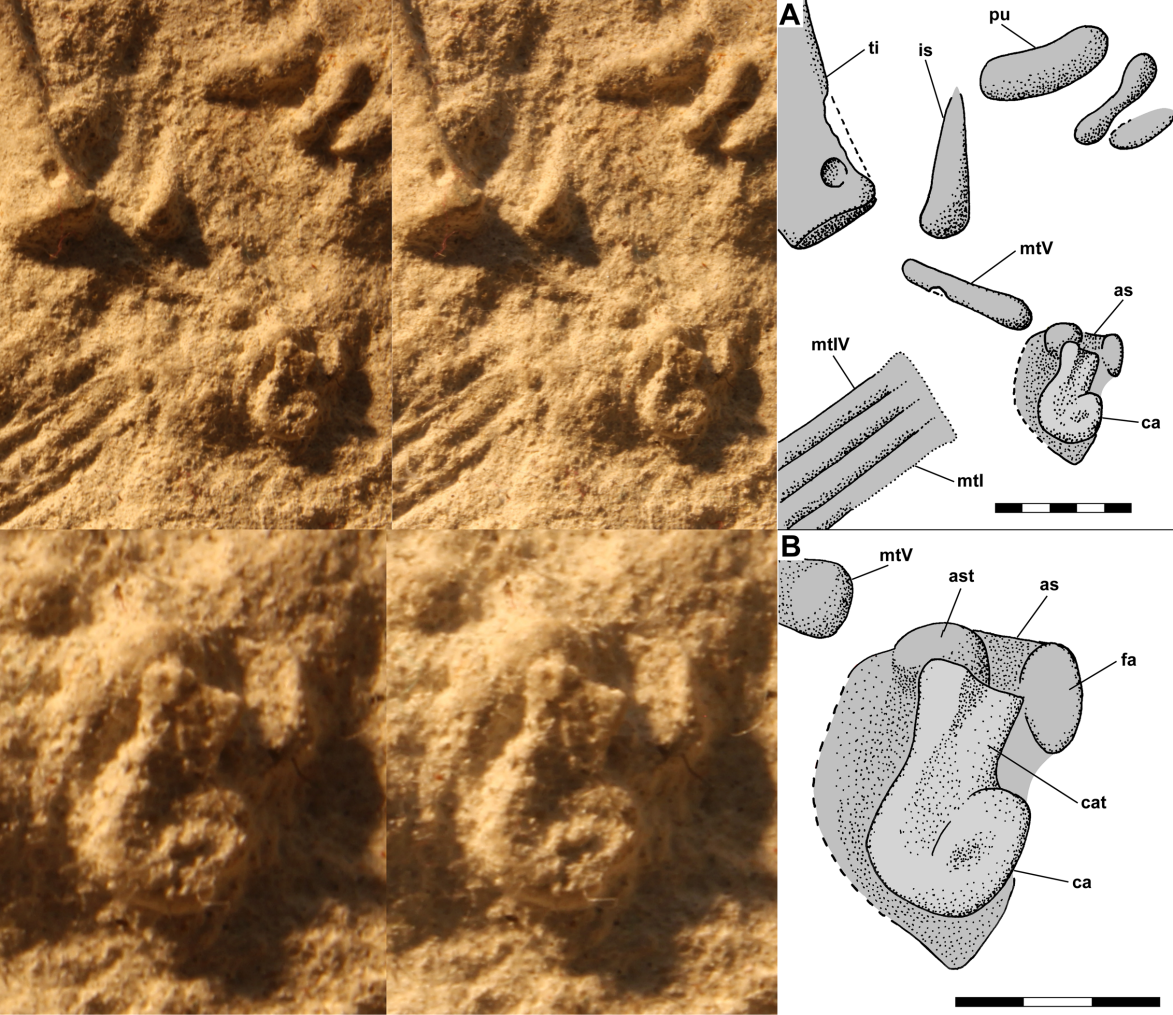
Stereo pairs of photographs and interpretive drawings of polyurethane rubber cast of ventral slab of *Scleromochlus taylori*, NHMUK R3557. (A) Left distal tibia and fibula, proximal tarsals and metatarsus. (B) Detail of proximal tarsals. Dashed lines indicate reconstructed outlines of elements based on information from the ventral slab, whereas dotted lines indicate information from the dorsal slab. Calcaneum lying atop the astragalus indicated with lighter gray. as, astragalus; ast, tubercle on astragalus; ca, calcaneum; cat, calcaneal tuber; fa, articular facet on astragalus; is, ischium; mt, metatarsal; pu, pubis; and ti, tibia. Scale bars = 5 and 3 mm.

The ventral slab preserves the somewhat disarticulated right tibia, tarsus, and metatarsus (Fig. 12). The distal articular surface of the tibia appears rather flat, and just proximal to it is an angular depression that appears unnatural, perhaps reflecting an angular crystal that grew within the matrix. The proximal tarsals are well preserved with the smaller calcaneum lying atop the astragalus. The calcaneum is 3.5 mm long and 2.3 mm wide with a subcircular body, which has a more complex shape than the simple shallow depression in its upper surface of previous illustrations (Padian, 1984: fig. 3a; Sereno, 1991: fig. 17; Benton, 1999: fig. 13e). There is a prominent tuber 1.6 mm wide and 1.6 mm long, which has a squared off posterior end. The astragalus is 4.9 mm long and ~3.5 mm wide. It has a rounded tubercle in the middle of the upper surface and an angled articular facet at one corner (upper right as illustrated in Fig. 12). The lower end presents a pointed corner, which corresponds to that of the astragalus of NHMUK R3556 (at upper left of inset in Fig. 10B). The margin to the right in the figure (the orientation of the astragalus is unclear) is mostly obscured by the calcaneum and the margin to the left is indistinct, but the shape was probably similar to that of NHMUK R3556. Mt I–IV are preserved in close contact with one another in plantar view, but their proximal ends and part of Mt I are indistinct. Mt V lies with its articular end adjacent to the astragalus.

**Figure 13:**
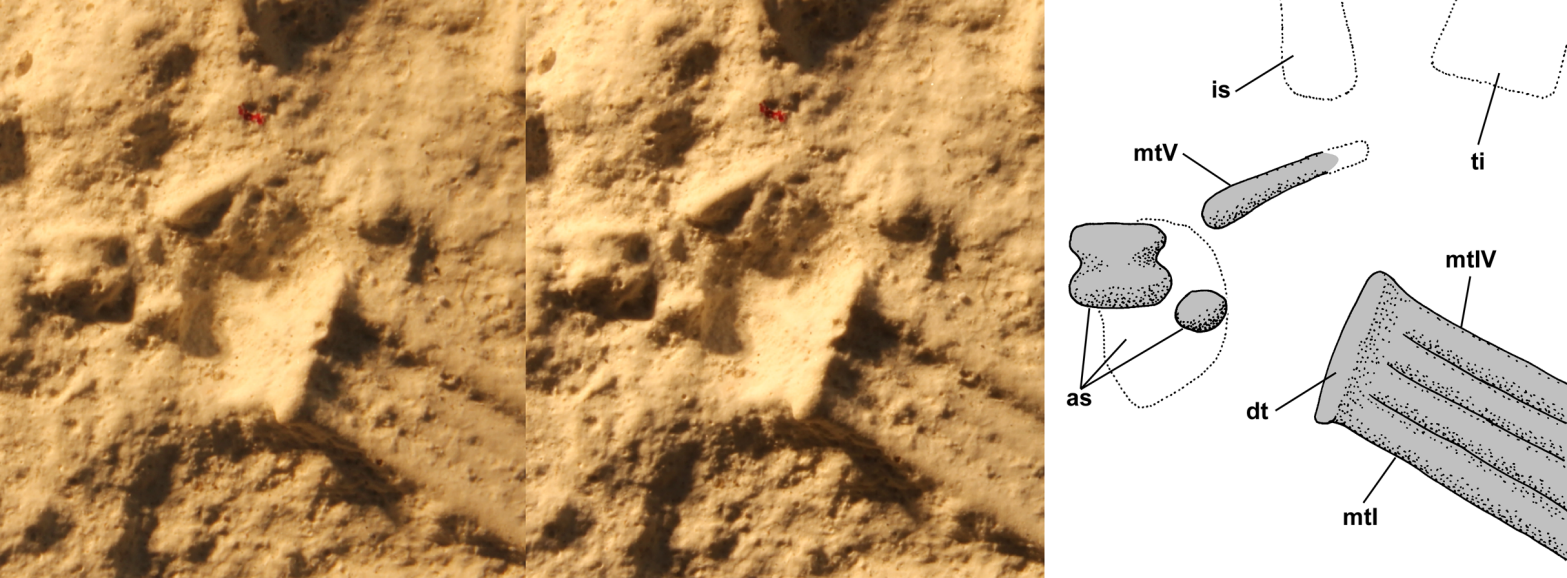
Stereo pair of photographs and interpretive drawing of polyurethane rubber cast of left distal tibia, proximal tarsals, and metatarsus of *Scleromochlus taylori*, NHMUK R3557, on dorsal slab. Dotted lines represent the position of the tibia and ischium on the ventral slab. as, parts of astragalus; dt, distal tarsals articulated with metatarsals I–IV; is, ischium; mt, metatarsal; and ti, tibia. Scale bar = 3 mm.

The dorsal slab preserves almost all of Mt I–IV in dorsal view and about two thirds of Mt V. Mt 1–4 are elongate with their proximal ends in contact with one another, the shafts cylindrical and closely appressed but without any sign of fusion. The articulated proximal ends of Mt I–IV form an oblique line such that the proximal end of Mt IV lies slightly proximal to that of Mt I. At the proximal ends of Mt I–IV there is a broad ridge raised above the level of their metatarsals, slightly expanded laterally, and faintly divided along the midline into two subequal parts. Based on comparison to NHMUK R3556, the ridge represents distal tarsals 1–4 indistinctly preserved in articulation with Mt I–IV. The silicone rubber peel provides better resolution to support the interpretation of the ridge as distal tarsals 1–4. The distal ends of the metatarsals do not exhibit any evidence of modification to support a habitual digitigrade posture with the metatarsus held at a steep angle to the substrate. About two thirds of Mt V is visible and compares well with what the ventral slab preserves. Adjacent to Mt V there is a roughly rectangular structure with depressions on the shorter sides and near one corner a small oval structure. Combining the information from the dorsal and ventral slabs, aligned on the basis of the relative positions of the metatarsus, indicates that the rectangular and oval structures probably are features of the astragalus (Fig. 13).

Benton (1999: fig. 4a) identified a ridge on the dorsal slab, just visible at the right edge of Fig. 12, as the distal fibula. However, combining information from the dorsal and ventral slabs indicates that the ridge represents the lateral margin of an indistinct negative impression produced by the tibia, which is better preserved on the ventral slab. Benton also illustrated but did not label a rounded lump between the distal tibia and the proximal ends of Mt I–IV. The surface texture of the lump is sufficiently irregular that I do not think it represents bone.

### NHMUK R3914

The specimen consists of dorsal and ventral slabs that preserve one individual. Combining their information, there has been significant disturbance of the head and neck, the left forelimb is not visible, and the right hindlimb and tail were beyond the posterior margin of the slabs (Fig. 3C). The right forelimb is loosely folded, whereas the left hindlimb is abducted at ~41°, the knee flexed to within ~19° of the crus, and the metatarsus flexed to within ~6° of the crus.

**Figure 14:**
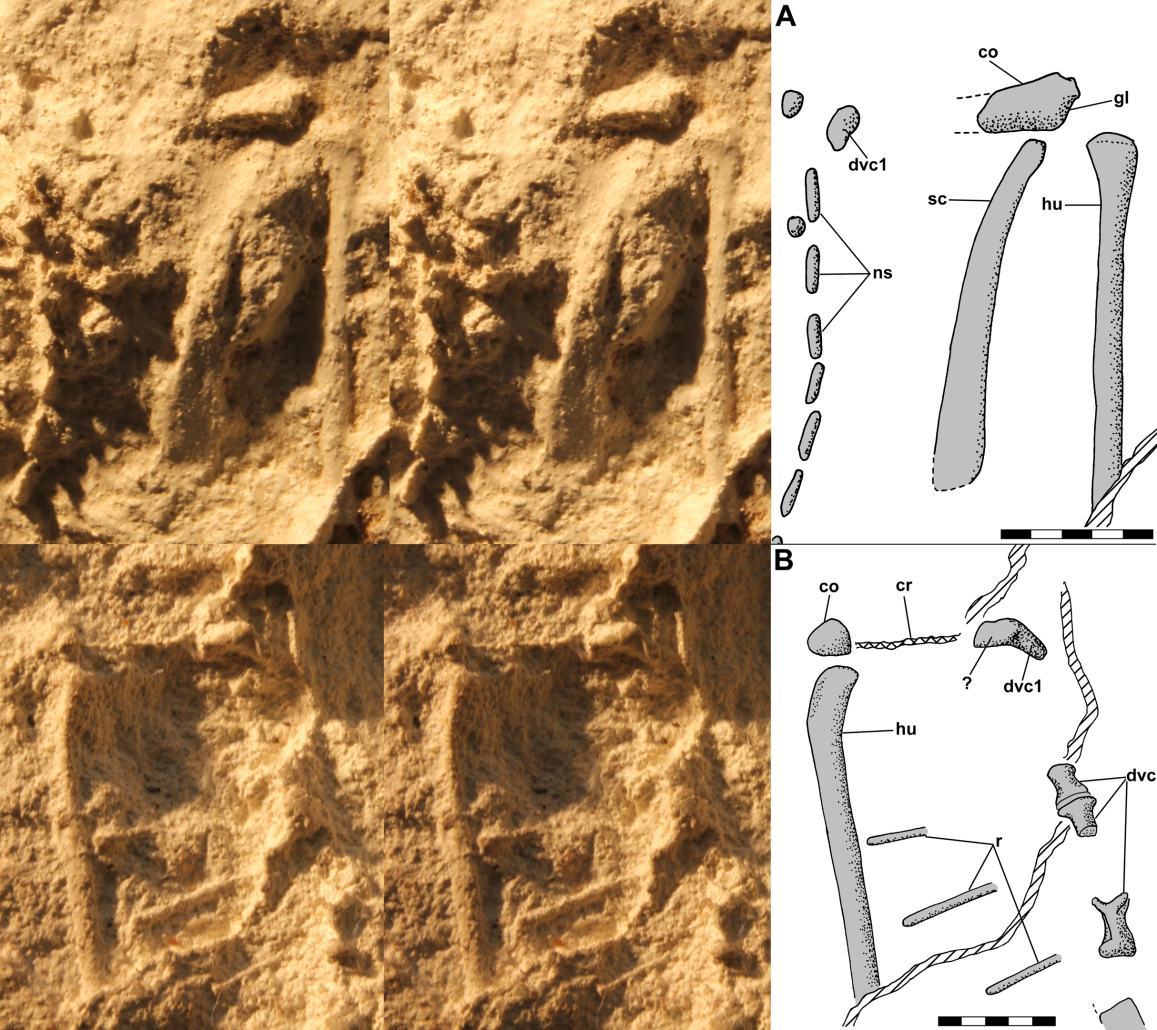
Stereo pairs of photographs and interpretive drawings of polyurethane rubber casts of right pectoral girdle and humerus of *Scleromochlus taylori*, NHMUK R3914. (A) In dorsal view on dorsal slab with articulated vertebral column at left. (B) In ventral view on ventral slab with vertebral column at right. The irregular ridges (hatched) running obliquely across the lower right of (A) and right side of (B) are cracks in the slab. Note the small ridge (cross-hatched) in *B* that represents a crack in the matrix extending from the coracoid toward the vertebral column. co, coracoid; cr, crack in matrix; dvc, vertebral centra; gl, glenoid fossa; hu, humerus; ns, neural spines; r, rib; sc, scapula; and ?, indeterminate element. Scale bars = 5 mm.

**Figure 15:**
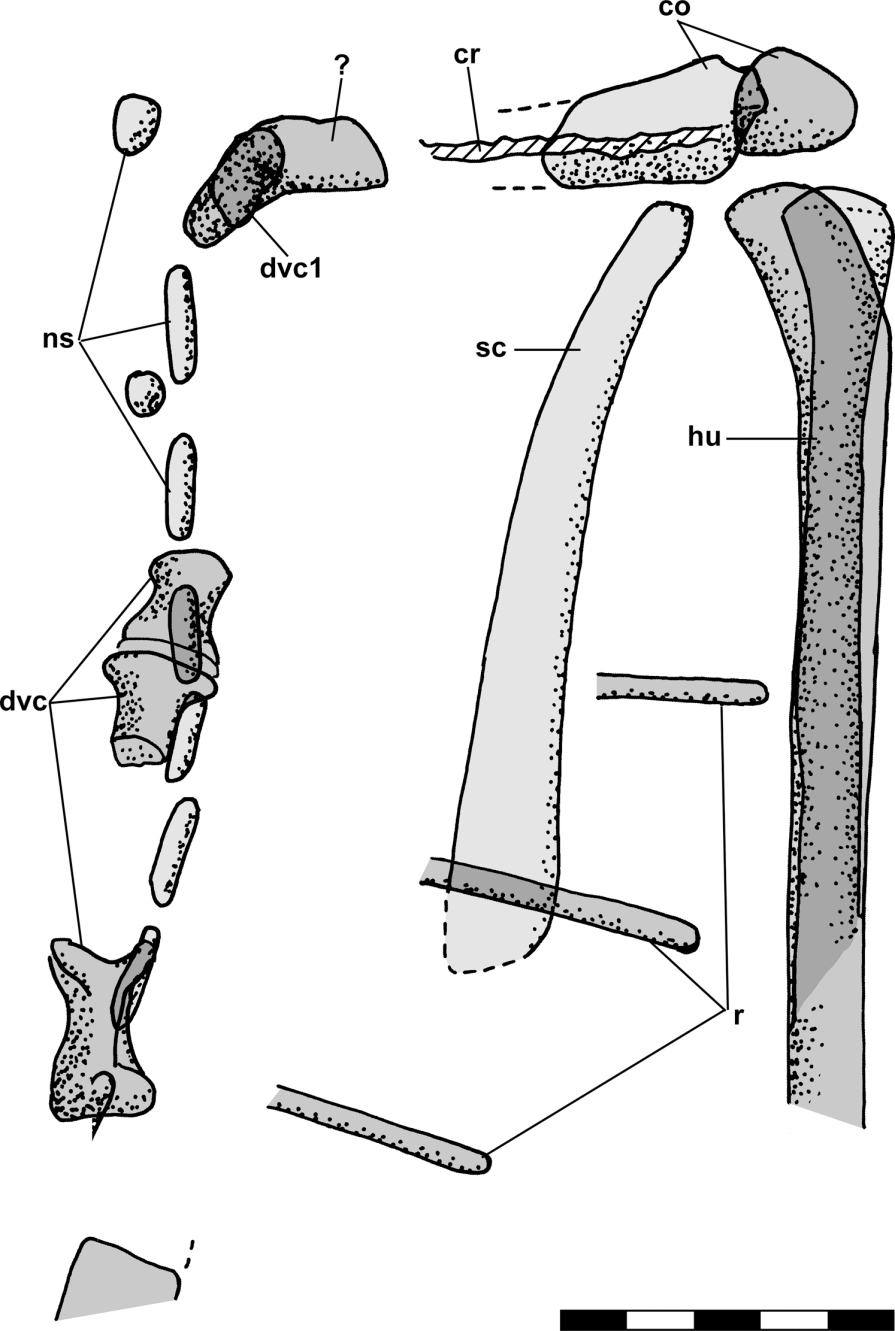
Composite interpretive drawing of right pectoral girdle and humerus of *Scleromochlus taylori*, NHMUK R3914, in dorsal view combining information from polyurethane rubber casts of the dorsal and ventral slabs. Note the small crack in the matrix extending from coracoid anterior to the humeral head toward the indeterminate element near the vertebral column, which may represent the medial end of an elongate coracoid. Elements on the dorsal slab indicated with light gray, elements on the ventral slab indicated with medium gray, and overlapping elements indicated with dark gray. Compare to Fig. 14. co, coracoid; cr, crack in matrix; dvc, dorsal vertebral centra; gl, glenoid fossa; hu, humerus; ns, neural spines; r, rib; sc, scapula; and ?, indeterminate element. Scale bar = 5 mm.

The specimen preserves an articulated dorsal vertebral column on both slabs. The dorsal slab preserves a series of seven dorsal vertebrae in dorsal view with intact blade-like neural spines (Fig. 14A). A small rounded lump lies ~1 mm lateral to the anterior end of the series of vertebrae, and directly medial to the coracoid. The ventral slab preserves four vertebral centra corresponding to the series of seven vertebrae on the dorsal slab (Fig. 14B). The first of them is angled posteromedially medial to the coracoid and is in contact with a transversely oriented subcylindrical mass. Combining the information from the dorsal and ventral slabs (Fig. 15) shows that rounded mass on the dorsal slab corresponds to the angled vertebral centrum on the ventral slab, and together they seem to be the first dorsal vertebra slightly displaced laterally relative to the subsequent vertebrae. However, it is not clear what the subcylindrical part is.

The combined length of the third through fifth dorsal vertebrae on the dorsal slab is 6.0 mm (2.0 mm/segment). Further posteriorly on the ventral slab there are four further articulated dorsal vertebrae, identified as the 10th though 13th. The combined length of the last three is 9.0 mm (3.0 mm/segment). The ventral slab preserves lateral parts of three dorsal ribs medial to the right humerus (Fig. 14B), and the distance between adjacent ribs increases laterally as in NHMUK R3146A. The lateral ends of the ribs are ~8.8 mm from the midline. On the left side there are three additional dorsal ribs preserved in articulation with dorsal vertebra 11–13 and angled posterolaterally, which Benton (1999) omitted from his figure 5b. The longest of the ribs extends 7 mm from the midline.

Note that Benton (1999) illustrated short segments of several ribs on the dorsal slab between the vertebral column and scapula. I cannot identify any such features that are clearly rib segments, and when I gave in to the pareidolia and identified vague linear features as rib segments, they did not align with the undoubted rib segments on the ventral slab. Therefore, I reject Benton’s interpretation that the dorsal slab preserves short rib segments.

The specimen preserves the right pectoral girdle and forelimb on both slabs (Fig. 14). On the dorsal slab, the humerus is preserved directed posteriorly with its head slightly lateral to, and so slightly disarticulated from, the lateral end of the coracoid and the anterior end of the scapula. The coracoid lies in a depression in the matrix and its margins pass medially into the wall of the depression with no suggestion of termination. What is exposed is roughly ovoid, 3.5 mm long, 1.3 mm wide, and appears to be of somewhat greater height. It exhibits a concave posterolaterally directed glenoid region. Benton (1999) stated that the full length of the coracoid was visible, but that is clearly not the case (Fig. 14A), and a uniformly rounded medial end would add another ~0.8 mm though there is no evidence that it was not longer. The scapula lies with its anterior end adjacent to the coracoid, and is ~12.1 mm long with a flat gently curving blade extending posteromedially. Both Huene (1914) and Benton (1999) illustrated the anterior end of the scapula as broadly expanded, but it does not appear as such on the polyurethane cast and silicone rubber peel.

The ventral slab preserves the lateral end of the right coracoid as a rounded mass immediately anterior to the humeral head (Fig. 14B). A slender ridge, which is too crooked to be a rib and presumably reflects a small crack in the matrix, passes medially from the coracoid toward the transversely oriented subcylindrical mass adjacent to the first dorsal vertebra. Combining the information from the dorsal and ventral slabs (Fig. 15) shows that the coracoid extended ~1.3 mm lateral to that preserved on the dorsal slab and the glenoid region of the coracoid was more robust than it appears on either slab. If one assumes that the medial end of the coracoid was rounded like the lateral end, then the coracoid would have been ~5.8 mm long, ~52% of scapular length, whereas if the medial end tapered away as illustrated by Benton (1999: figs. 5a and 5b), then the coracoid would have been considerably longer. In the latter case, the crack passing medially from the coracoid on the ventral slab might reflect the presence of an elongate medial end of the coracoid buried in the matrix, and the transversely-oriented subcylindrical mass may be part of the coracoid, clavicle, or interclavicle.

### NHMUK R4823/4824

The specimen consists of separately numbered dorsal and ventral slabs, each broken into anterior and posterior pieces with some intervening matrix missing from NHMUK R4824, which together preserve one individual. It is not clear who first identified NHMUK R4823 as dorsal and NHMUK R4824 as ventral, but based on labels with the casts that include A.D. Walker’s handwriting (M.J. Benton, 2016, personal communication) that identification was accepted by Walker when he was making the PVC casts. Sereno (1991) accepted the identification and described the well preserved humerus of NHMUK R4824 as a left in medial view, and Benton (1999) also accepted those identifications. However, NHMUK R4823 preserves a large section of articulated dorsal osteoderms in a depressed area with articulated series of dorsal vertebrae and their neural spines on higher planes anterior and posterior to the depressed area. Based on the relative positions of the vertebrae and the osteoderms, NHMUK R4823 is the ventral slab and NHMUK R4824 the dorsal. The misidentification of the slabs might have resulted from Taylor or whoever made the initial identification of the slabs following Woodward’s (1907) interpretation of osteoderms as gastralia such that NHMUK R4823 was interpreted as dorsal.

Combining the information of the two slabs, there has been significant disturbance to the head and the left side of the body, and the skull is not visible or unrecognizable. The neck is bent to the left, the trunk is quite straight, and the tail bent strongly to the right so as to pass anteriorly lateral to the right hindlimb (Fig. 3F). Both humeri are directed laterally with the antebrachia directed anteromedially with the right elbow flexed at ~29°. The right hindlimb is directed anteriorly along the body with the knee and ankle fully flexed. It seems likely that the left femur was also directed anteriorly along the body, but the crus and pes have been pulled anterolaterally into the area of disturbance. Note that I disagree with Benton’s (1999: fig. 6) interpretation of the large long element on the left side of the trunk as the radius and ulna and the jumble of elements adjacent to its lateral end as carpals, metacarpals, and manual phalanges. Instead, based on its length (>26 mm) and width and its proximity to the jumbled elements, the large long element is interpreted as the left tibia and fibula, the supposed carpals are interpreted as tarsals, and the elongate jumbled elements are interpreted as Mt I–IV and pedal phalanges. In addition, whereas I concur with Benton’s interpretation of the right hindlimb on the ventral slab (Benton, 1999: fig. 6), what he interpreted as the femur on the dorsal slab is interpreted as the tibia and fibula in contact and what he interpreted as the tibia and fibula in contact is interpreted as the closely appressed metatarsals based on the distance from the vertebral column and the morphology of the metatarsals.

**Figure 16:**
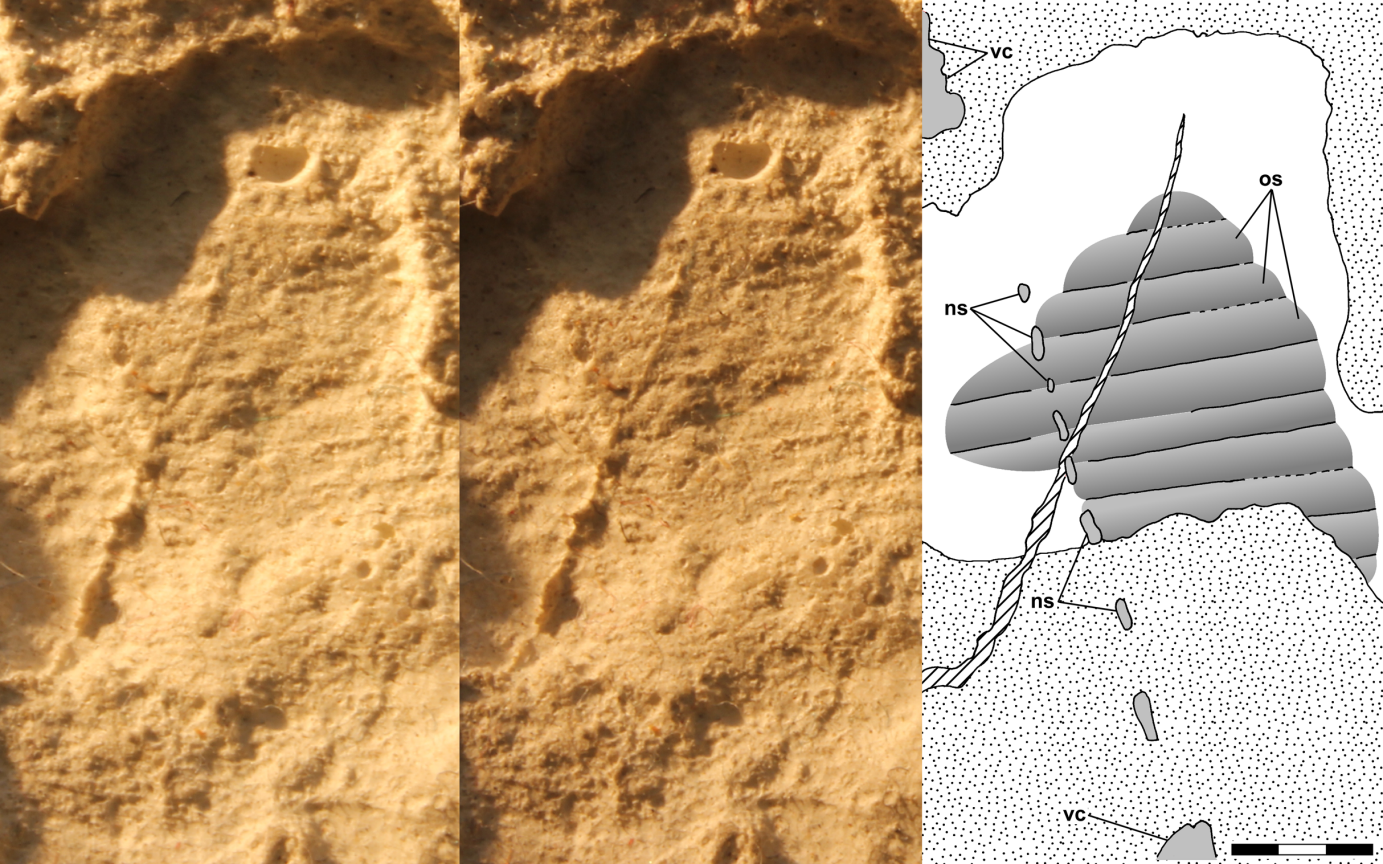
Stereo pair of photographs and interpretive drawing of polyurethane rubber cast of dorsal osteoderms of *Scleromochlus taylori*, NHMUK R4823, in ventral view on ventral slab. The oblique feature (hatched) is a crack in the slab. The irregular stipple is the layer of matrix that broke away to expose the osteoderms. ns, neural spine; os, osteoderm; and vc, vertebral centrum. Scale bar = 3 mm.

Parts of the vertebral column are preserved in articulation on the ventral slab. Benton (1999) illustrated a series of three dorsals near the posterior end of the scapula and another of four or so posterior dorsals near the pelvis. Both are rather poorly preserved and difficult to measure. Between the two series, the layer of matrix upon which the vertebral centra were preserved is broken away to expose neural spines of some intervening vertebrae, and preserved in place on an even deeper (i.e., more dorsal) layer there are traces of additional neural spines and the ventral surface of an articulated series of 10 osteoderm segments (Fig. 16). The traces of neural spines run longitudinally across the series of osteoderms and indicate the body midline where right and left osteoderm elements presumably attached to one another and the neural spines. The osteoderms are better preserved on the left side where the anteroposterior extent of the series is ~11.5 mm and the transverse extent is ~7.3 mm. One of the osteoderm segments appears narrower anteroposteriorly than the others, but that may have resulted from one segment sliding to partially overlap the adjacent segment as the carcass was flattened and the series of osteoderms longitudinally compressed. Most osteoderms exhibit a faint lineation running transversely across the middle of the element, perhaps a slight ridge. The osteoderm elements do not exhibit significant transverse curvature, which indicates that the dorsal body surface was transversely flattened rather than rounded.

**Figure 17:**
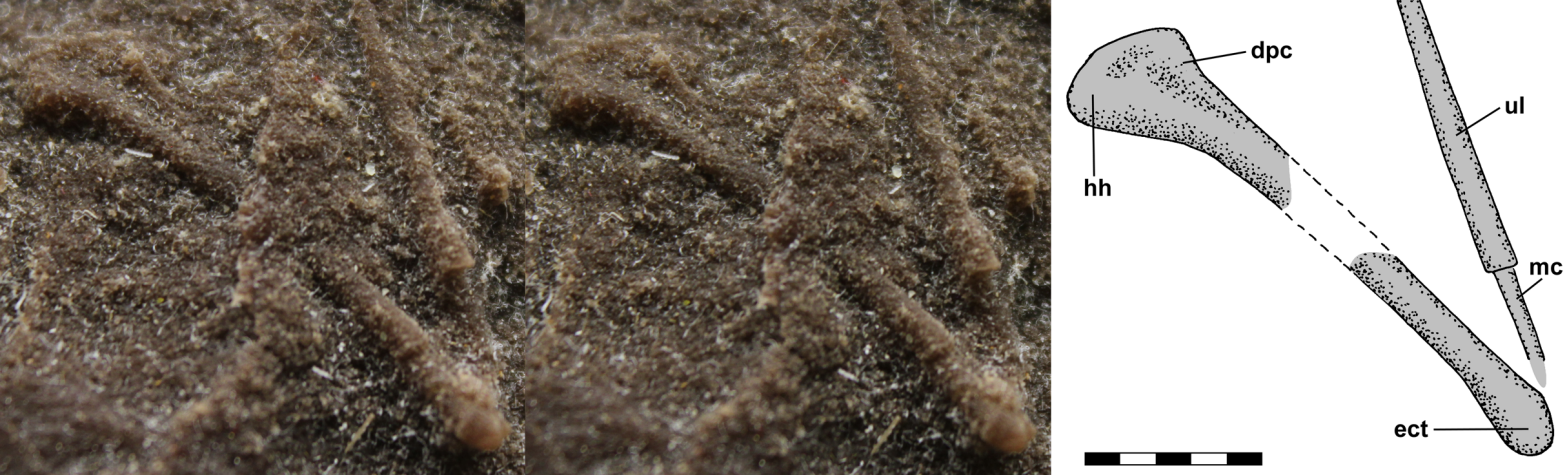
Stereo pair of photographs and interpretive drawing of PVC cast of right humerus and ulna of *Scleromochlus taylori*, NHMUK R4823, in lateral view on dorsal slab. Dashed lines indicate reconstructed midshaft of humerus. dpc, deltopectoral crest; ect, ectepicondyle; hh, humeral head; mc, medullary canal; and u, ulna. Scale bar = 5 mm.

The right humerus is well preserved in lateral view on the dorsal slab (Fig. 17). It exhibits a prominent head, large deltopectoral crest, robust shaft, and large ectepicondyle. A short section of the midshaft is not visible, but the proximal and distal ends are aligned such that the shaft was intact when preserved. The midshaft diameter of the humerus measured on a photograph is ~1.2 mm. An epipodial is preserved in a position that suggests that it was in articulation with the humerus. Benton (1999) interpreted the element as the radius in keeping with his interpretation of the humerus as a left in medial view, but it is here interpreted as the right ulna in lateral view because of its large diameter (~0.93 mm measured on a photograph). Much of the ulna’s shaft is preserved, but toward the proximal end it is represented by a smaller cylinder ~0.5 mm in diameter. I interpret this as representing the medullary cavity, which would be the case if some of the ulna’s diaphyseal wall remained in the negative impression when the PVC cast was made; however, I did not think to confirm that that was the case when I was in the NHMUK. The cross-sectional area of the medullary cavity would have been ~53% of that of the shaft, thus the diaphyseal walls were not particularly thin. The right humerus and antebrachium are also preserved on the ventral slab, but more poorly.

### NHMUK R5589

The specimen consists of a dorsal slab alone that preserves one individual that seems to have been in a position similar to that of NHMUK R3556 with its head, neck, and anterior trunk skeleton bent strongly to the left (Fig. 3E). The right forelimb seems to have been directed posterolaterally with the antebrachium flexed to within ~16° the humerus. The right hindlimb is directed anterolaterally at ~41°, the crus flexed to within ~16° of the femur, and the ankle flexed at 38°. The left hindlimb was directed anterolaterally at ~35°, the knee fully flexed, and the ankle flexed at ~74°. There seems to have been significant disturbance of the trunk because some anterior dorsal and perhaps cervical vertebrae are disarticulated and scattered and the scapula and articulated distal right humerus and antebrachium lie well away from the vertebral column.

The specimen preserves much of the preorbital part of the skull in dorsal view, and Benton (1999) seems to have based his interpretation of the upper jaw tip as broadly rounded in dorsal view on this specimen. However, based on my examination, the rounded anterior edge of the impression of the right premaxilla is not the natural finished anterior margin of the premaxillae but rather is the result of a subconical premaxilla (and nasal?) being intersected by the fracture plane of the slab. The tip of the premaxillae and the narial region would have been anterior to the preserved part of the skull, and so NHMUK R5589 provides no evidence as to the shape of the upper jaw tip or the size and position of the external nares.

Various vertebrae and ribs are scattered within the disturbed region. Among them, a dorsal vertebra and right rib are preserved in articulation in posterior view (Fig. 7B). The vertebra exhibits a subcircular posterior surface of the centrum, broad and low neural arch with prominent zygapophyses, and a neural spine of moderate height. The rib extends ~8 mm laterally from the arch and 9.7 mm from the midline, and is nearly straight. Benton (1999: fig. 2) illustrated the rib as broken in two, but it is not clear to me that it was and there is no evidence of significant angular displacement. A short distance anterior to those elements are two curving ribs, one overlapping the other such that they present an appearance that though not symmetrical is reminiscent of the two ribs of NHMUK R3146B that Benton (1999) interpreted as a chevron. The radii of ribs are ~10 and ~17 mm.

There are a few articulated dorsal osteoderms preserved between the left femur and vertebral column, more or less where osteoderms are preserved in NHMUK R3146A. In addition, there are 12 scattered elements behind the right metatarsus. Benton (1999: fig. 7) illustrated the elements, but did not label or discuss them, and though the PVC cast of the specimen includes that part of the slab, the polyurethane cast and silicone rubber peel I examined ended at the posterior margin of the right metatarsus and omitted the elements. The elements are elongate, flat, and up to ~6 mm long with a uniform width of ~1 mm. They are too long and too numerous to be pedal phalanges from the adjacent pes, and their shapes and dimensions match those of osteoderms of NHMUK R3146A and R4823/4824, so they are interpreted as disarticulated osteoderms.

## Discussion

Benton & Walker (1985) reviewed the vertebrate fauna of the Lossiemouth Sandstone, which consisted of the herbivores *Stagonolepis* (length up to 2.7 m), *Hyperodapedon* (1.3 m) and *Leptopleuron* (40 cm), the carnivores *Ornithosuchus* (up to 3.5 m), *Erpetosuchus* (60 cm) and *Saltopus* (60 cm), the omnivore *Brachyrhinodon* (25 cm) and smallest of all, *Scleromochlus* (17 cm per Benton, 1999), which made up 5% of the fossils. Although Benton & Walker (1985) considered *Scleromochlus* to be an omnivore, its isodont unicuspate teeth suggest it was carnivorous and its small size and broad skull suggest it preyed on insects and other suitably sized invertebrates. Given its small size, *Scleromochlus* was presumably preyed upon by *Erpetosuchus, Saltopus* and juvenile *Ornithosuchus*.

Benton & Walker (1985) described the Lossiemouth Sandstone as lying atop a claystone or siltstone and consisting of up to 30 m of eolian sandstone with uniform grain size and large-scale cross-beds. They cited several authors (e.g., Gordon, 1892) writing while the Lossiemouth Sandstone was being quarried and its fossils collected, who stated that the vertebrate fossils were found only in a thin bed of soft yellowish sandstone at the base of the formation below the main thickness of hard siliceous sandstone that was quarried for building stone. Fraser & Benton (1989: p. 431) stated that there were no plant or invertebrate fossils in the Lossiemouth Sandstone and noted that it represented an environment that was “inimical to vertebrate life.” They noted that the vertebrates had lived in a well-vegetated lowland that was overwhelmed by an advancing dune field. Although Benton (1999) described the depositional environment of the Lossiemouth Sandstone as sandy and dune-like, I cannot find any evidence in the *Scleromochlus* specimens to suggest that they were adapted to sand, desert, or dune environments. None of the other denizens of the Lossiemouth Sandstone, i.e., *Stagonolepis* (Walker, 1961), *Hyperodapedon* (Benton, 1983), *Ornithosuchus* (Walker, 1961, 1964), *Leptopleuron* (Säilä, 2010), *Erpetosuchus* (Benton & Walker, 2002), *Brachyrhinodon* (Fraser & Benton, 1989) and *Saltopus* (Benton & Walker, 2011), have been described in the most recent publications as living in or being adapted to sand, desert, or dune environments. Thus, it would seem that the entire vertebrate fauna of the fossiliferous horizon at the base of the Lossiemouth Sandstone was adapted to the depositional environment that laid down the claystone or siltstone below rather than to the sandy dune environment above, and the fossiliferous horizon probably recorded the local extinction of that vertebrate fauna.

Benton & Walker (1985) noted that most vertebrates from the Lossiemouth Sandstone were preserved in natural resting poses and suggested that they had died naturally, were quickly buried by sand, and their skeletons were articulated and undisturbed except by settling of the sand. Whereas that sequence of events is plausible, it is also plausible that the vertebrates adopted natural resting poses while sheltering from sandstorms, were quickly buried by sand, suffocated, and their skeletons were articulated and undisturbed except by settling of the sand. Certainly the latter sequence of events could have applied to *Scleromochlus* because it would not take much accumulated sand to bury and suffocate the small animal. Bones in the Lossiemouth Sandstone typically are not crushed or distorted; however, Benton & Walker (1985) noted that some large bones such as an ilium and caudal vertebra of *Stagonolepis* were crushed and distorted, and noted that one skull of *Ornithosuchus* was compressed to half its original height. Although many skeletons are articulated and undisturbed, Benton & Walker noted that in some cases there was clear evidence of local disturbance by scavenging. As for *Scleromochlus*, although the skulls seem to have suffered dorsoventral compression, with the exception of the flattened shaft of the left femur of NHMUK R3557 I can find no evidence that other individual skeletal elements were significantly crushed or distorted. However, three of the six *Scleromochlus* specimens (NHMUK R3557, R4823/4824, R5589) exhibit significant disturbance of the trunk region (i.e., much more than would be expected from sand settling; Fig. 3) that probably resulted from scavenging, perhaps by invertebrates as the disturbances affect only parts of the small skeletons.

Benton & Walker (1985: p. 221) stated that *Scleromochlus* specimens were preserved in “a natural squatting pose,” and although there does not seem to be any direct sedimentological evidence as to which slab is the upper and which the lower, there is no reason not to interpret them as preserving individuals lying on their venters. I consider a natural resting position to be one that an animal would normally adopt in life when at rest, and if an animal died and was buried in the Lossiemouth sands in such a position and, after decomposition and desiccation of its soft tissues and compaction of the sand, the resulting fossil was essentially intact other than being flattened and exhibiting minor disarticulations resulting from the disintegration of ligaments and settling of the sands, then it would reflect the natural position in life and could be considered natural as well. That is what seems to have happened to the *Scleromochlus* specimens because the symmetry of fore and hindlimb positions in several specimens and similarity of hindlimb positions between several specimens argue strongly that the positions are natural and that the limb posture was sprawling. Flattening might have changed the angles of joints somewhat, but it is hard to imagine how compression of a hindlimb folded compactly in a parasagittal plane could swing the femur out ~45° as in NHMUK R3556, R3914 and R5589, much less the ~90° of NHMUK R3146A without disturbing the adjacent individual. Note that specimens of the erect bipedal dinosaur *Citipati* that were buried by dunes while sitting on nests are preserved with their folded hindlimbs still in roughly parasagittal planes (Norell et al., 1995, 2018), essentially undisturbed by the settling and compaction of the sands.

Benton (1999: p. 1424) characterized the preservation of *Scleromochlus* as “dorso-ventral “roadkill” orientation, with the forelimbs and hindlimbs extended out to the sides.” I do not know what roadkill meant to Benton, but given my experience with American drivers it implies to me that the animal was violently squashed into an unnatural position, and when Benton was made aware of that view, he made no attempt to disabuse me. Benton (1999) made no attempt to reconcile his roadkill characterization with the view of Benton & Walker (1985) that specimens were preserved in natural squatting poses, articulated and undisturbed, and he did not provide any evidence or argumentation that preserved body and limb postures were unnatural. Specimens of *Stagonolepis* (Walker, 1961), *Hyperodapedon* (Benton, 1983), *Ornithosuchus* (Walker, 1964), *Saltopus* (Benton & Walker, 2011), *Leptopleuron* (Säilä, 2010) and *Brachyrhinodon* (Fraser & Benton, 1989), the latter two almost as small as *Scleromochlus*, are preserved with body positions and limb postures similar to those of *Scleromochlus* specimens and were not described as roadkill or unnatural. In sum, I can find no evidence to suggest that the preserved body and limb postures of *Scleromochlus* are unnatural, and conclude that the preserved body and limb positions are natural and that the preserved joint angles provide reliable information as to the range of motion of joints.

The preserved body and limb postures of *Scleromochlus* also seem to preserve the behavior of the animals immediately before death (Fig. 3). Most obvious in this regard is NHMUK R3146, which preserves two individuals snuggled side by side in similar positions with the trunk straight and the limbs folded. It is unusual to find two articulated specimens of terrestrial vertebrates preserved so close together (e.g., *Peltobatrachus* (Panchen, 1959) and the Mongolian fighting dinosaurs (Osmólska, 1993)) except in “traps” that accumulate skeletons over a long time period (e.g., California’s La Brea Tar Pits, Wyoming’s Natural Trap Cave), and their close association strongly suggests that the two individuals were interacting in life. NHMUK R3146B would seem to have moved to lie alongside NHMUK R3146A, as if seeking shelter of some kind, and if it were shelter from blowing sand then turning its head into the neck of NHMUK R3146A would have sheltered its nostrils. Both individuals have their forelimbs arranged symmetrically, those of NHMUK R3146A have the humeri flexed anteriorly somewhat probably because NHMUK R3146B had come up from behind, whereas NHMUK R3146B has its humeri extended posteriorly somewhat probably because it had moved into its position alongside NHMUK R3146A from behind. The outer hindlimbs of the pair (i.e., the left of NHMUK R3146A and right of NHMUK R3146B) are folded with the femora directed anterolaterally and the knees fully flexed so that the crus would have been tight against the thigh. The right femur of NHMUK R3146A was directed laterally perhaps because it was lying under NHMUK R3146B and the left femur of NHMUK R3146B is abducted ~30° perhaps because it was lying over the tail of NHMUK R3146A. NHMUK R3557, R3914 and R4823/4824 are preserved in poses similar to those of NHMUK R3146A and R3146B with the trunk straight and the limbs folded. Dinosaurs and other vertebrates with erect limb posture are almost never found in positions with the femora abducted in the horizontal plane as are those of *Scleromochlus*, whereas the fact that several specimens of *Scleromochlus* are preserved in the same position suggests that it was the normal resting posture.

**Figure 18:**
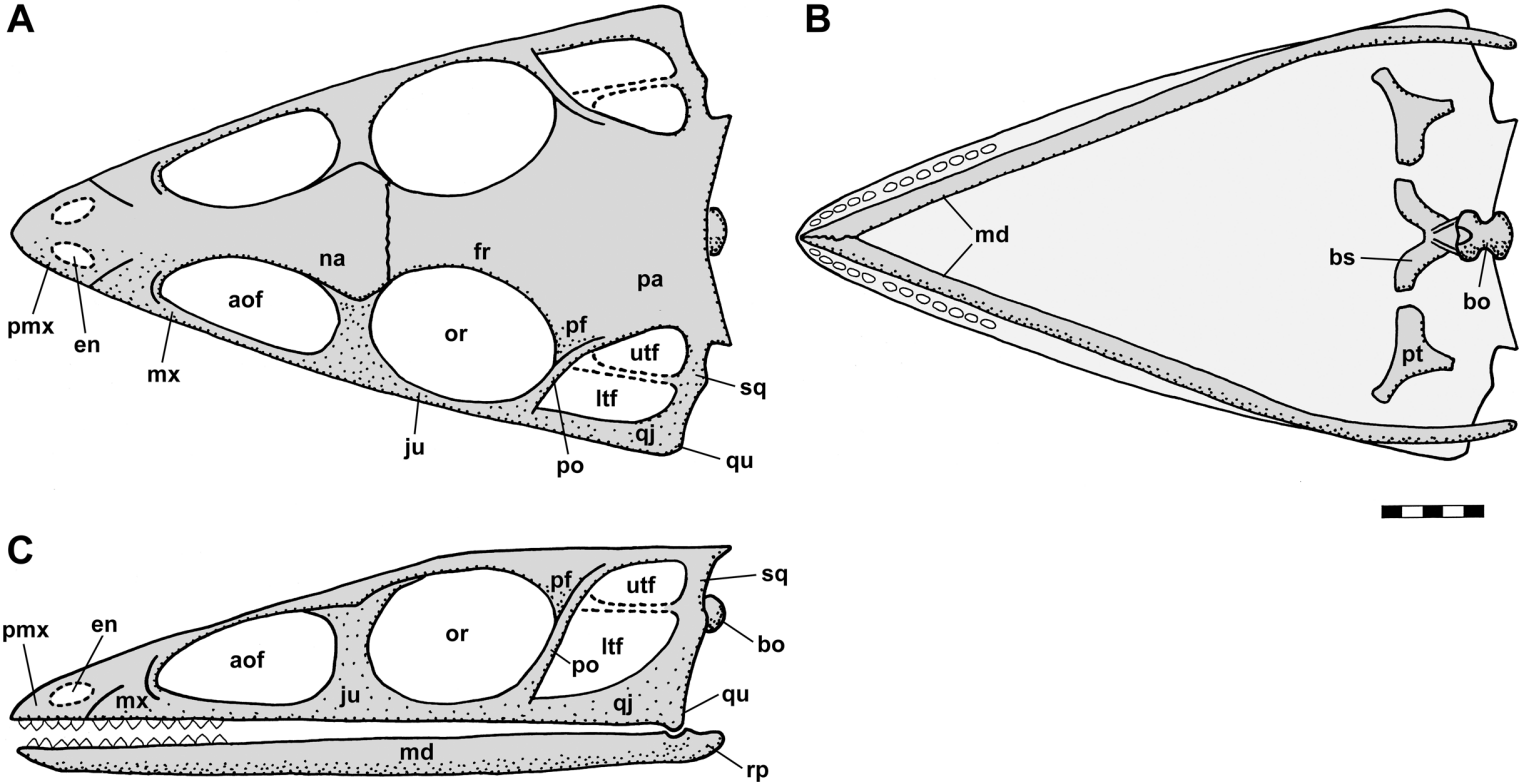
Reconstruction of the skull of *Scleromochlus taylori* based primarily on NHMUK R3146A in dorsal (A), ventral (B) and left lateral (C) views. Size and shape of the external naris is conjectural. aof, antorbital fenestra; bo, basioccipital; bs, basisphenoid; en, external naris; fr, frontal; ju, jugal; ltf, lower temporal fenestra; md, mandible; mx, maxilla; na, nasal; or, orbit; pa, parietal; pf, postfrontal; po, postorbital; pmx, premaxilla; pt, pterygoid; qj, quadratojugal; qu, quadrate; sq, squamosal; and utf, upper temporal fenestra. Scale bar = 5 mm.

**Figure 19:**
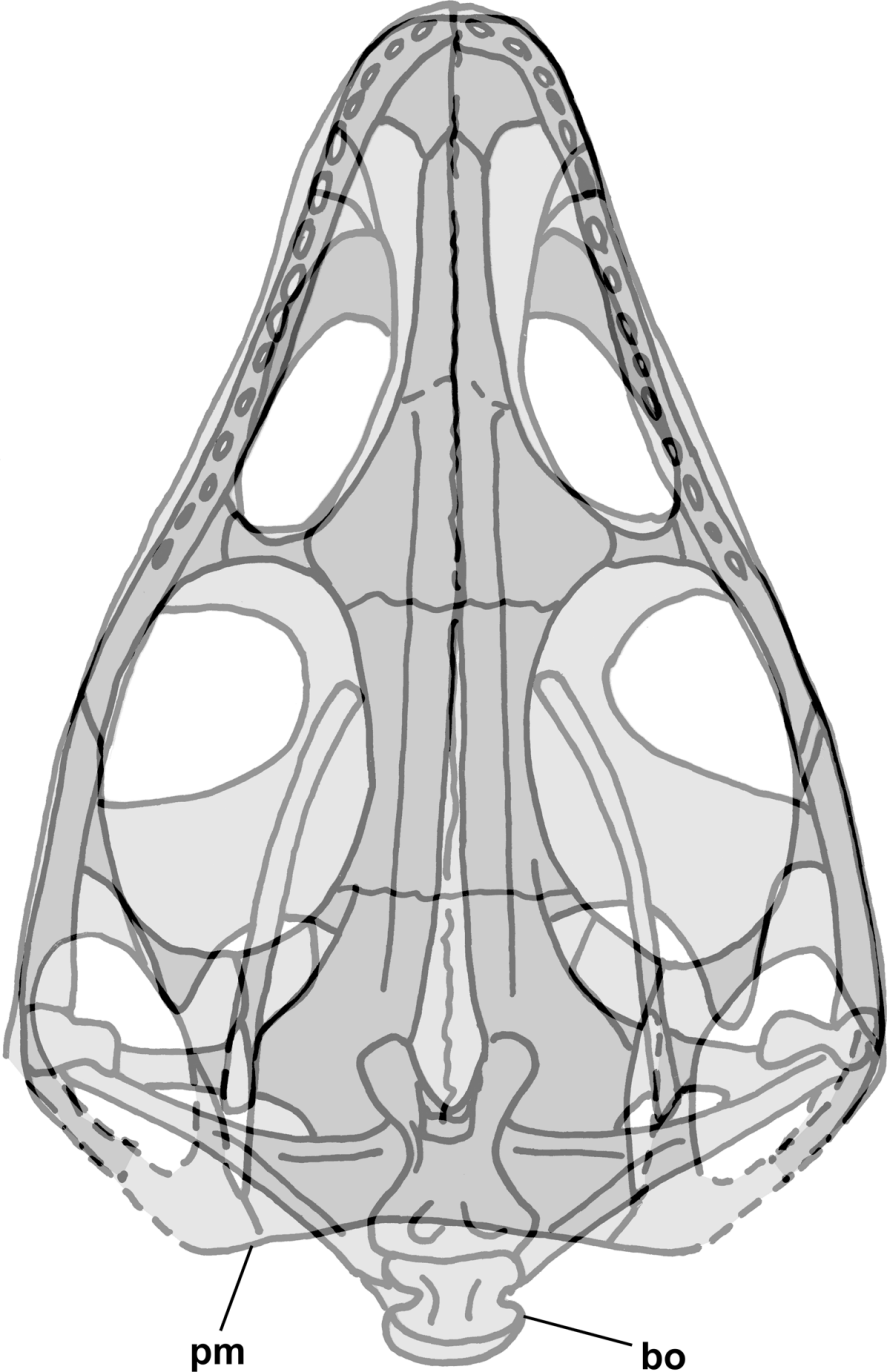
Composite of the dorsal and ventral views of Benton’s (1999; Figs. 8A and 8B) reconstruction of the skull of *Scleromochlus taylori* as redrawn in Figs. 2F and 2G. Note the basioccipital extending a full vertebral length behind the posterior margin of the occiput. bo, basioccipital; pm, posterior margin of parietal.

In contrast to those specimens, NHMUK R3556 and R5589 are preserved with the head, neck, and trunk strongly flexed to one side (Figs. 3D and 3E). Those poses cannot be explained by postmortem opisthotonos, but would be consistent with a single individual sheltering from blowing sand by turning its body tightly to one side so that its head and nostrils were sheltered in the lee of its body. Note that the posterior dorsal vertebrae of the two specimens of *Scleromochlus* are preserved with little lateral flexion despite the strong lateral flexion of their anterior dorsal vertebrae, whereas specimens of *Brachyrhinodon* (Fraser & Benton, 1989) and *Leptopleuron* (Säilä, 2010) preserved with the head, neck, and trunk strongly flexed to one side exhibit essentially uniform curvature of the entire presacral vertebral column. It is not surprising that the posterior dorsal vertebrae exhibit little lateral flexion in those specimens of *Scleromochlus* preserved with the trunk relatively straight, but the absence of significant lateral flexion of the posterior trunk is surprising in specimens with the anterior trunk strongly flexed to one side, which suggests that either the intervertebral joints of the posterior dorsal vertebrae or something else limited lateral flexion. There is another significant difference between the preserved limb postures of *Scleromochlus* and *Leptopleuron*; whereas the femora of *Leptopleuron* are typically directed laterally (e.g., 80–110°) with the knees and ankles flexed at 90° (Säilä, 2010; figs. 9, 11–19) and only RSM 1891.92.528 has the crus flexed close to the femur, the hindlimbs of *Scleromochlus* are most often preserved with the femora directed anterolaterally with the knees fully flexed or nearly so and the ankles often fully flexed or nearly so. Such limb postures of *Scleromochlu*s are unlikely to have resulted from violent squashing, and suggest that *Scleromochlus* was doing something different with its hindlimbs than *Leptopleuron*.

It should be noted that the preservation of two individuals side-by-side and of individuals with strongly laterally flexed bodies could also be consistent with specimens preserved in burrows. However, *Scleromochlus* does not exhibit burrowing adaptations (see below) and whereas infilled burrows typically differ from surrounding sediments there is no evidence from the matrix to suggest that the specimens were preserved in burrows. In addition, the significant disturbance of parts of several specimens, presumably by scavengers, seems less likely to have occurred to carcasses buried in burrows than those shallowly buried in newly drifted sand.

### Osteology and function

#### Skull

A detailed skull reconstruction was prepared based primarily on NHMUK R3146A and R3556 (Fig. 18). Note that Fig. 2 illustrates previous reconstructions of *Scleromochlus’*s skull, all scaled to uniform length, so as to facilitate comparisons. NHMUK R3146, R3556 and R3557 preserve relatively complete skulls that demonstrate the general shape and structure of the skull, but the upper jaw tip, narial region, palate, and occiput are incompletely known or unknown. The skulls demonstrate that in dorsal view there was a broad temporal region, a slight convex curve in the orbital region, and a straight taper from the orbital region to the jaw tip. NHMUK R3146A seems to demonstrate that the anterior end of the upper jaw curved with a small radius to a pointed tip, markedly different from that which Benton (1999) reconstructed. The right premaxilla of NHMUK R5589, upon which Benton seems to have based his interpretation of a broadly rounded premaxillary tip, does not preserve a finished anterolateral margin of the premaxilla and so does not provide evidence of a rounded tip, and likewise the upper jaw tips of NHMUK R3146B, R3556 and R3557 all appear truncated or broken and do not preserve finished tip margins. There is also no evidence of a concave jaw margin lateral to the antorbital fenestra, and the concave curve reconstructed by Benton may be resulted from combining a broadly rounded premaxillary tip with the more posterior parts of the skull. The straight taper of the upper jaw reconstructed here would have resulted in a premaxillary-maxillary tooth row that was closely aligned with, and just lateral to, the dentary tooth row.

No specimen preserves evidence of external nares. The suggestion of Benton & Walker (1985) that the naris had a lateral flange as a specific adaptation to living in sandy environments cannot be supported, whereas the suggestions of Woodward (1907) and Benton (1999) that the external nares were small and terminal are probably correct because evidence of the nares probably would be present in the preserved skulls if the nares were larger or more posteriorly placed.

The skulls of NHMUK R3146, R3556 and R3557 demonstrate that the antorbital fenestra was oval to subtriangular with a rounded anterior margin, and that of NHMUK R3146A demonstrates that its anterior margin was bounded by a prominent ridge. The shape of the posterior margin is unclear because the preorbital bar consisting of prefrontal and lacrimal is broken in all specimens. In some specimens there may be traces of bone preserved within the antorbital fenestra, which Sereno (1991) and Benton (1999) interpreted as representing an antorbital fossa; however, neither Sereno nor Benton provided any evidence or argumentation that the bone traces within the antorbital fenestra are internal portions of the maxilla or nasal forming an antorbital fossa. Note that although there is little evidence of the palate other than traces of the vomers, pterygoids, and possible palatine fragments, Benton (1999; Fig. 2G) reconstructed the palate as having very large internal nares that extended as far back as the posterior margin of the antorbital fenestrae. If the external nares were small and terminal, then there is no reason to think that the internal nares would be so extensive. In the end, I can find no evidence that bone traces within the antorbital fenestrae are not palatal elements, and thus no evidence that there were antorbital fossae.

The orbits, as preserved in NHMUK R3146 and R3556, appear as anteroposteriorly elongate ovals in dorsal view. The orbit also appears oval in lateral view in the skull reconstruction (Fig. 18), in which the dorsoventral diameter of the orbit between the narrow frontals forming the dorsomedial margins and the laterally placed jugals forming the ventrolateral margins is roughly equivalent to the anteroposterior diameter such that the orbits would have been subcircular and faced dorsolaterally. Dorsoventral compression of the skull would have distorted the dorsolaterally facing subcircular orbits into anteroposteriorly elongate ovals.

The orbit and its eyeball were large relative to the skull. That might suggest that *Scleromochlus* specimens were immature given the marked negative allometry of orbit and eyeball size relative to skull size common in diapsids. However, no specimen seems to exhibit size independent indicators of skeletal immaturity (e.g., unfused bones, incompletely ossified bones with simple shapes; Bennett, 1993, 1995), and the bones of the pelvic girdle of NHMUK R3557 appear fused and even the small distal tarsals of NHMUK R3556 seem to be well ossified. Therefore, all *Scleromochlus* specimens are interpreted as mature adults, in which case although the sclerotic ring is not known the large orbit and presumably large eyeball suggest that *Scleromochlus* could have been crepuscular (Schmitz & Motani, 2010).

Woodward (1907) and Sereno (1991) reconstructed sizeable upper temporal fenestrae within the area identified here as paired parietals, but I can find no evidence of such fenestrae. Benton & Walker (1985) and Benton (1999) reconstructed slit-like upper temporal fenestrae just below the plane of the parietals; however, the preserved shape of the left squamosal of NHMUK R3146A suggests that the upper temporal bar separating the upper and lower fenestrae was close to the inferolateral ends of the preserved squamosal. The unidentified displaced element on the left of NHMUK R3146A is interpreted here as part of the upper temporal bar.

Benton (1999) reconstructed the basioccipital of *Scleromochlus* as extending posterior to the posterior margin of the parietals as can be seen by combining his dorsal and palatal views of the reconstructed skull (Fig. 19). However, combining information from the dorsal and ventral slabs of NHMUK R3556 provides no evidence that the basioccipital was in such a position, and neither do other specimens, and NHMUK R3556 suggests that the basisphenoid was angled anteroventrally and the basioccipital extended only slightly behind the parietals.

NHMUK R3146A demonstrates that the quadrate region of *Scleromochlus* formed a roughly right angled posterior corner of the skull in dorsal view. The quadrate extended dorsomedially to contact the squamosal extending ventrolaterally from the broad parietals. Benton (1999) reconstructed the quadrate as slanting anterolaterally in dorsal view and anteroinferiorly in lateral view, presumably based on the anterolaterally slanting left posterior margin of the skull of NHMUK R3146B on the dorsal slab. However, that slanting margin seems to be an artifact, and the right quadrate and quadratojugal of NHMUK R3146A and the right quadrate and quadratojugal of NHMUK R3146B are at nearly right angles with the jaw margin in dorsal view such that the quadrate would have been roughly vertical in lateral view.

NHMUK R3146 and R3556 demonstrate that the mandibular rami were slender and straight anteriorly and met in a short symphysis that tapered to a sharp point in ventral view and seems to have tapered upward to the point in lateral view. Combining information from the dorsal and ventral slabs of NHMUK R3146A shows that the posterior parts of the rami curved medially to some extent and the jaw articulations were near their posterior ends with only short retroarticular processes (Fig. 6). Benton & Walker (1985) and Benton (1999) reconstructed the mandible with long retroarticular processes, which would suggest that *Scleromochlus* exhibited powerful depression of the mandible, but neither offered an explanation as to why *Scleromochlus* might have had a long retroarticular process or powerful depression. The more posterior position of the jaw articulation reconstructed here would mean that the upper and lower jaws were effectively longer and the gape greater for a given angle of depression than if the jaw had been shorter.

Benton (1999) reconstructed the mandible as shallow anteriorly but deeper posteriorly with a convex dorsal margin beneath the orbit, and characterized it as “slipper-shaped,” whereas Benton & Walker (1985) and Sereno (1991) reconstructed the mandible as shallow in lateral view. Benton’s (1999) interpretation seems to have resulted from misinterpretation of the upper and lower jaws of NHMUK R3556 on the ventral slab (Fig. 8), whereas that specimen is interpreted here as providing no evidence of a deep posterior mandible. Note that the right upper and lower jaws of NHMUK R3146B on the ventral slab present a similar appearance to that of NHMUK R3556 in that the upper jaw lies lateral to the ramus and the two might be misinterpreted as evidence of a broad ramus in lateral view (see Benton, 1999: fig. 2b).

Huene (1914) and Benton (1999) reconstructed an external mandibular fenestra and Benton & Walker (1985) illustrated one with a dashed outline. Neither Huene nor Benton & Walker presented evidence of a fenestra and Benton’s (1999) interpretation of a possible fenestra seems to have been based on the right ramus of NHMUK R3557 on the ventral slab. However, based on my examination of NHMUK R3556 and R3557, neither specimen provides evidence of a fenestra, for example, finished edges on the bones that would surround a fenestra, and there is no reason to think that fenestrae would have been present in the shallow rami.

NHMUK R3146A and R3557 preserve traces of teeth. NHMUK R3146A exhibits three premaxillary alveoli, but probably had two additional alveoli anteromedial to the three, which agrees with the five premaxillary teeth reconstructed by Benton & Walker (1985) and Sereno (1991). NHMUK R3146A also exhibits seven maxillary alveoli, whereas NHMUK R3557 seems to have had eight maxillary teeth. Thus, *Scleromochlus* had at least 13 teeth in the upper jaw, but it might have had more. Huene (1914) reconstructed 14 and 13 teeth in the upper and lower jaws, respectively, Benton & Walker (1985) 18 and 16, Sereno (1991) 20 and 16 and Benton (1999) 15 each. The best preserved tooth of NHMUK R3146A seems not to have been fully erupted, but if reconstructed with the widest part of the crown slightly above the margin of the alveolus, then the length of the tooth probably would not exceed its diameter, which would agree with the teeth of NHMUK R3557 (see Benton, 1999: fig. 10d). NHMUK R3146A suggests that there may have been variation in the size of premaxillary alveoli and so variation in the diameter and perhaps the length of the premaxillary teeth, but the maxillary alveoli vary little suggesting little to no variation in maxillary tooth size. Sereno (1991) and Benton (1999) reconstructed the teeth as long and posteriorly curved like theropod teeth, but there is no evidence of curvature or marked lateral compression or evidence that the teeth were as long as reconstructed by those authors. Given the small size of the teeth, they probably would have been suited to holding small invertebrate prey before swallowing whole, but would not have been suited to biting off pieces of large food items or processing food items in the mouth before swallowing.

Reconstructions of the skull of *Scleromochlus* show it as strongly dorsoventrally compressed with height:width ratios ranging from 0.5 to 0.62 (Fig. 2; Supplemental Information). Dorsoventrally compressed skulls are found in terrestrial vertebrates that exhibit sprawling postures and often bring the skull into contact with the substrate, for example, frogs and lizards, whereas erect sauropsids that hold their skull above the substrate do not have dorsoventrally compressed skulls, for example, theropods with height:width ratios >1.15. The strongly dorsoventrally compressed skull of *Scleromochlus* is inconsistent with an erect posture, but is consistent with a sprawling posture.

#### Vertebral column and ribs

Huene (1914) and Benton (1999) reconstructed the vertebral series as consisting of eight cervical vertebrae and 12–13 and 16–17 dorsal vertebrae, respectively (Table 3). NHMUK R3146A & B provide information as to the length of the presacral vertebral column, but only NHMUK R3556 preserves more than short isolated series of vertebral centra and the number of dorsal vertebrae is unclear. Here the cervical series is interpreted as consisting of seven vertebrae based on NHMUK R3556, which preserves seven cervical vertebrae in articulation with the basioccipital anteriorly and dorsal vertebrae posteriorly, and in which the positions of the right coracoid on the ventral slab and anterior end of the left scapula on the dorsal slab mark the position of the cervico-dorsal transition. The cervicals have anteroposteriorly broad neural spines, with that of the axis being larger than the subsequent cervicals. NHMUK R3146A and B also seem to have seven cervicals based on the relative positions of the posterior skull margin and the cervico-dorsal transition.

**Table 3:** Reconstructed vertebral formulae of *Scleromochlus taylori*. Where authors did not provide complete formulae, numbers in square brackets were counted from skeletal reconstructions based on the assumptions that the cervico-dorsal transition was coincident with the anterior margin of the pectoral girdle (but see Bennett, 2014 for discussion of problems inherent in such determinations) and that sacrals were fully bounded laterally by ilia. See text for Discussion.

Author	Cervicals	Dorsals	Presacrals	Sacrals	Caudals
Woodward (1907)	(6–7)	(15)	~21	4	(51)
Huene (1914: fig. 33)	8	12–13	20–21	4	≥50 (62)
Sereno (1991: fig. 18b)	(7)	(17)	(24)	(3)	(25)
Benton (1999)	8	16–17	24–25	4	~35
This work	7	18	25	3	≥35

The cervical series of NHMUK R3556 suggests a length of ~2.2 mm/segment, and one cervical centrum of NHMUK R3146A on the ventral slab has a length of ~2.3 mm. NHMUK R3146A does not exhibit articulated anterior dorsal centra, but the length from the 1st to 8th dorsal ribs on the articulated left side of the ribcage on the dorsal slab suggests a length of 2.8 mm/segment. A series of three dorsal vertebral centra of NHMUK R3914 on the ventral slab, which are interpreted as dorsals 11–13, has a length of 3.1 mm/segment, and a series of three mid-dorsal vertebral centra of NHMUK R3557 on the ventral slab has a length of ~3.5 mm/segment. However, the three dorsal vertebrae of NHMUK R3557 just anterior to the sacrum on the dorsal slab have a length of ~2.6 mm/segment, and the three sacral vertebrae of NHMUK R3146A on the dorsal slab have a length of ~2.3 mm/segment. The various measurements suggest that the anterior dorsal vertebrae were longer than the cervicals, posteriormost dorsals, and sacrals. If one reconstructs the dorsal vertebral series as 49 mm long from the cervico-dorsal transition to the first sacral as in NHMUK R3146A, and assumes that the anterior 13 dorsal vertebrae had an average length of 2.8 mm/segment based on the spacing of the anterior eight ribs, then there would be 12.6 mm remaining for a series of posterior dorsals gradually decreasing in length to meet the ~2.3 mm long sacrals. The average segment length of the series would be ~2.5 mm, and so there would be five such dorsals, bringing the number of dorsals to 18, whereas Benton (1999) reconstructed the dorsal vertebral series as ~42 mm long and consisting of 17 vertebrae.

Woodward (1907), Huene (1914) and Benton (1999) reconstructed the sacrum as consisting of four vertebrae. Only NHMUK R3146B preserves articulated sacral vertebrae and ilia in dorsal view such that the articulation can be observed, and it clearly shows there are at least three sacral vertebrae. However, the ilia do not seem to be long enough to fully accommodate articulation with a fourth sacral. Similarly, the positions of the ilia and adjacent vertebral centra on NHMUK R3556 suggest that there were only three sacral vertebrae. Therefore, I interpret *Scleromochlus* as having only three sacrals.

No specimen preserves a complete caudal series. I accept Benton’s (1999) interpretation of the preserved caudal vertebrae as indicating a minimum of ~35 vertebrae, but can find neither evidence that tail was not longer than 35 vertebrae nor evidence of terminal caudal vertebrae. Benton (1999) reconstructed the caudal vertebral series as ~80 mm long, whereas my measurements of NHMUK R3557 suggest that the tail was at least 90 mm long and may have been longer.

Woodward (1907: fig. 3b) and Huene (1914: fig. 9) described and illustrated disarticulated chevrons no longer than the mid-caudal vertebral centra on NHMUK R3557. Both described them as delicate, though Huene stated that Woodward illustrated them as too massive. I saw no evidence of chevrons on any specimen. However, I accept that Woodward and Huene saw small chevrons on NHMUK R3557, and suspect that the impressions were degraded by the time Walker made the PVC casts, indeed Huene’s description that they were more delicate than Woodward illustrated might have been based on observations of partially degraded impressions. In his skeletal reconstruction, Huene (1914), who illustrated the tail with 62 caudal vertebrae, illustrated the 24th caudal vertebra as bearing the last chevron, and illustrated caudal neural spines as small and virtually absent by the 40th vertebra. He commented that transverse processes were absent in mid-caudal vertebra, and both he and Woodward (1907) described the tail as slender. I concur that the tail probably was rather slender, and although it probably could have been used as a dynamic stabilizer of body orientation it probably did not have enough mass to be an effective counterbalance for the head, neck, and trunk when in a static bipedal pose.

The dorsal ribs of NHMUK R3146A extend laterally up to 9.4 mm from the midline, curve slightly posteriorly, diverge from one another laterally and exhibit a gentle posterior curvature reminiscent of the ribcage of *Phrynosoma cornutum* (Hodges, 2001) and seem to have curved ventrally only near their lateral ends. The dorsal ribs of NHMUK R3914 extend up to 8.8 mm from the midline and that of the 13th vertebra was at least 7 mm long. NHMUK R5589 preserves an isolated dorsal vertebra and rib in articulation in posterior view, which shows that the rib was straight and extended laterally 9.7 mm from the midline without significant ventral curvature. There is no evidence that the straightness of that rib resulted from compression, and combining that information with the gentle posterior curvature (~17 mm radius) of the ribs of NHMUK R3146A suggests that the dorsal ribs were straight in anterior view and curving posteriorly in dorsal view for much of their length. In addition, the isolated ribs of NHMUK R3146A and R5589 (Fig. 7) exhibit similar curvatures (~17 mm radius) and do not suggest that the ribs were curved in posterior view in addition to the curvature in dorsal view. Ribs are preserved on most specimens, but I found no examples with radii of less than ~10 cm and no ribs curved ventrally to a significant degree so as to result in a rounded rather than dorsoventrally compressed trunk. The smaller radius of a few ribs may reflect the transition between the neck and trunk. Ribs straight in anterior view would have resulted in a broad, flat dorsal surface, and so the trunk presumably was dorsoventrally compressed. One might argue that the lack of curvature resulted from crushing of the specimen, but crushing could not explain the fact that the isolated rib of NHMUK R5589 preserved in posterior view was straight in posterior view or the fact that the radii of the isolated ribs of NHMUK R3146B and R5589 ranged from 10 to 17.5 mm. The dorsoventrally compressed ribcage, like the dorsoventrally compressed skull, is inconsistent with erect posture, but is consistent with sprawling.

#### Osteoderms

The posterior trunk of NHMUK R3146A preserves transverse striations visible under low angle illumination, which Woodward (1907) and Huene (1914) interpreted as gastralia. Benton (1999: p. 1433; see also Benton & Walker, 1985: p. 213) argued that they were dorsal structures that lay over the vertebral column and interpreted them as “probably very thin scutes or horny scales.” I concur that they were dorsal, but it is not clear what Benton meant by scute or scale because although he did not label the structures in his figure 2, the similar structures of NHMUK R3557 and R4823 in his figures 4 and 6 were labeled as osteoderms and yet his cladistic analysis coded *Scleromochlus* as lacking osteoderms. Furthermore, Romer (1956) used scute and scale to refer to both cornified epidermis and dermal bone, and both cornified epidermal shields of the chelonian shell and dermal bones of crocodilian dorsal body armor are commonly referred to as scutes. It is unlikely that the structures in *Scleromochlus* were cornified epidermis because they occur on four of the six specimens of *Scleromochlus*, whereas keratinized structures (e.g., claw sheathes of unguals, epidermal body scales) seem not to have been preserved in any specimens of *Scleromochlus* or other taxa from the Lossiemouth Sandstone. Therefore, the structures are interpreted as osteoderms, i.e., bony structures formed within the dermis.

The best preserved articulated series of osteoderms is that of NHMUK R4823 in which there are bilateral pairs of osteoderms with each element having an anteroposterior length of ~1.15 mm and a transverse width of ~7.3 mm, thus roughly two segments of osteoderm per vertebral segment. I found no evidence of additional osteoderms lateral to the bilateral pair. NHMUK R3146A and R4823 demonstrate that the osteoderms were closely spaced with no gap evident between one segment and the next. NHMUK R3557 demonstrates that the osteoderm elements were firmly connected to one another because it preserves a series of osteoderms still in tight articulation even though significantly displaced to the side in a disturbed area.

The osteoderms seem to have been restricted to the posterior half of the trunk. The anterior margin of the osteoderms of NHMUK R3146A lies behind the 7th dorsal rib, roughly in line with the knee when the hindlimb is folded at rest. In NHMUK R4823, it is not possible to count dorsal vertebrae or ribs, but based on the position of the anterior margin of the preserved osteoderms relative to the positions of the humerus and knee on the ventral slab, it appears that the anterior margin is in a similar position. In NHMUK R3557 an articulated series of osteoderms lies more anteriorly on the ventral slab, but it is displaced to the left within the area of disturbance and so does not provide clear evidence of a more anterior extent.

The osteoderms covering the posterior trunk formed a structure here termed a culet (in armory, a culet is a piece of plate armor consisting of small, horizontal plates or lames articulated so as to protect the small of the back or buttocks). Although the culet could have had a protective function, there is no evidence that one segment could normally slide over another and so the firmly connected osteoderms would have severely limited lateral flexion of the posterior trunk such that the culet probably was responsible for the reduced lateral flexion of the lumbar vertebral column evident in some specimens.

#### Pectoral girdle and forelimb

NHMUK R3914 preserves the best example of the pectoral girdle of *Scleromochlus*, which consists of a long scapula and a robust coracoid (Figs. 14 and 15). The scapula is strap-like, ~12.1 mm long, and lies more or less parallel to the vertebral column with its anterior end curving laterally toward the humerus. NHMUK R3146A exhibits a comparable scapula lying parallel to the vertebral column, but its anterior end curves medially and the proximal ends of the humeri are quite close together, probably because dorsoventral compression drove the glenohumeral joints medially. The right coracoid on the dorsal slab of NHMUK R3914 disappears into the medial wall of a depression in the matrix without any evidence of a termination medial to the depression, and the small crack passing medially from the lateral end of the coracoid on the ventral slab might be an indirect trace of an elongate medial part of the coracoid. Combining information from the dorsal and ventral slabs shows that the coracoid had a robust lateral end and was at least ~5.8 mm long (Fig. 15), thus ~52% of scapula length even if the crack on the ventral slab and the transversely oriented subcylindrical mass had nothing to do with the coracoid. The shoulder joint had a large preserved range of motion, ~150° in the horizontal plane, and there is no reason to think that motion would have been restricted in the vertical plane.

The forelimb, though much shorter than the hindlimb, was long relative to the body. The humerus had a prominent proximally positioned deltopectoral crest. Although impressions of the humerus on several casts suggest that the humeral shaft was rather slender, they probably do not reflect the full diameter of the shaft, and the impression of NHMUK R4824 on the dorsal slab shows the shaft diameter was ~1.2 mm (compare to tibia and fibula midshaft diameters of NHMUK R3556 of 1.75 and 0.80 mm, respectively). Like the humeral impressions, impressions of the radius and ulna may not reflect the full diameters of their shafts, but the impression of NHMUK R4824 on the dorsal slab shows the ulnar shaft was ~0.95 mm in diameter with rather thick walls. The elbow’s preserved range of motion is from fully flexed to ~87°, but it seems likely that it could have been extended considerably further. The manus was small, and it seems unlikely that it could have reached the mouth to pass food items to the mouth or assist in manipulating food, so the forelimb presumably was used primarily for locomotion.

#### Pelvic girdle and hindlimb

The pelvic girdle was shallow with rather short processes and an imperforate acetabulum. The longest ilium preserved is that of NHMUK R3556, 8.4 mm long, and the ilia are shorter than the series of four adjacent vertebrae in all specimens. Benton (1999: fig. 4C; see also Fig. 11A) illustrated the pelvic girdle of NHMUK R3557 as preserving a pubis and ischium that extended ~4.3 and ~7.9 mm from the center of the acetabulum, respectively. Such a pelvic girdle with ischium longer than pubis is similar to that of *Euparkeria*, whereas Benton’s (1999: fig. 14; see also Sereno, 1991: fig. 18c) skeletal reconstruction shows a theropod-like pelvic girdle with pubis and ischium extending ~8.5 and ~6.8 mm from the center of the acetabulum, respectively. I found no evidence of a long pubis in any specimen, and a long pubis would be inconsistent with the dorsoventrally compressed trunk.

The femur of NHMUK R3557 has a weakly inturned hemispherical femoral head with the articular surface covering much of the proximal end, which combined with the imperforate acetabulum probably would not have permitted the femur to swing in a parasagittal plane, and even if the hip joint could be adducted to lie close to a parasagittal plane, further adduction for control movements would be impossible and the joint would be unsuitable for erect locomotion. Even if one assumed that the hip joint morphology was not a hindrance to such movement, the prominent tubercle on the pubis would have been. Instead, the hip morphology suggests the femur could be flexed anteriorly, extended posteriorly, and abducted laterally to a considerable degree (~68° in NHMUK R3146B), but could not be adducted to swing fore and aft in a parasagittal plane. Thus, the hip joint and femur were not suited to erect bipedal locomotion with parasagittal gait, but sprawling to semi-erect postures were possible. The knee joint consists of a distal femur with two prominent condyles separated by an intercondylar groove and a rather flat proximal tibia, and has been interpreted as a hinge joint that allowed flexion and extension without significant rotation (Padian, 1984; Benton, 1999). I agree with that interpretation. The preserved range of motion is from fully flexed to ~68°, but there is nothing in the morphology of the distal femur and proximal tibia to suggest that the knee could not be extended to ~180°, such that the knee would be suited to be both leaping and erect running.

The interpretation of the tarsus has been critical to the interpretation of the locomotion and relationships of *Scleromochlus*, and the two right tarsals of NHMUK R3557 preserved lying in contact with one another on the ventral slab have been central to the interpretation of the tarsus. Both Woodward (1907) and Huene (1914) illustrated the upper, smaller of the two and interpreted it as a calcaneum with prominent tuber, and Martin (1983) concurred. Padian (1984) illustrated that tarsal and the larger tarsal of NHMUK R3556 and interpreted them as lateral and medial distal tarsals, respectively, like those of pterosaurs, but seems to have overlooked the small distal tarsals of NHMUK R3556 that can be seen on the polyurethane cast that he made. Sereno (1991) illustrated both tarsals of NHMUK R3557 and interpreted the larger as distal tarsal 3 and the smaller as distal tarsal 4, but also seems to have overlooked the small distal tarsals of NHMUK R3556 and did not comment on the larger tarsal of NHMUK R3556 figured by Padian, which should have been visible on Sereno’s latex peel because it was made after Padian’s polyurethane cast. Benton (1999) described distal tarsals 1–4 of NHMUK R3556 and so interpreted the two tarsals of NHMUK R3557 as proximal tarsals, but did not comment on Padian’s illustration of the larger proximal tarsal of NHMUK R3556 and reversed Woodward’s and Huene’s identifications based on comparison to the proximal tarsals of *Lagosuchus* (Sereno, 1991: fig. 9). Benton (1999: fig. 13d) illustrated the two proximal tarsals of NHMUK R3557 as of nearly the same size, and Sereno (1991: fig. 17) illustrated the larger medial tarsal as only slightly larger than the smaller. However, comparison of the tarsals of NHMUK R3557 with the larger tarsal of NHMUK R3556 reveals that both Sereno and Benton overlooked the pointed tip of the larger tarsal of NHMUK R3557, i.e., the triangular feature in Fig. 12B extending toward the bottom of the figure from beneath the body of the smaller tarsal. Therefore, the larger proximal tarsal was roughly twice the size of the smaller.

The medial elements of the crus and tarsus of basal archosauriforms were significantly larger than the lateral elements, see for example *Proterosuchus, Euparkeria, Rutiodon*, *Riojasuchus* and *Lagosuchus* (Sereno, 1991; figs. 3, 4, 6, 7 and 9), with the tibia significantly larger than the fibula and the astragalus significantly larger than the calcaneal body from which the tuber, if present, projected. In *Scleromochlus*, too, the tibia was significantly larger than the fibula, i.e., in NHMUK R3556 tibial and fibular shaft diameters are 1.75 mm and 0.8 mm, respectively, and the one proximal tarsal was roughly twice the size of the other (i.e., 4.9 × 3.5 mm vs. 3.5 × 2.3 mm). On that basis, the larger proximal tarsal is interpreted as medial and the astragalus and the smaller tarsal as lateral and the calcaneum with prominent tuber. So identified, they compare well with the astragalus and calcaneum of *Proterosuchus, Euparkeria, Rutiodon* and *Riojasuchus* (Sereno, 1991), in which the astragalus was larger and subrectangular and the calcaneum was smaller and bore a prominent tuber, but they do not compare well with those of *Lagosuchus* (Sereno, 1991). Based on the morphology of the astragalus and calcaneum and comparisons to the tarsi of *Proterosuchus, Euparkeria, Rutiodon*, *Riojasuchus* and *Lagosuchus*, the tarsus of *Scleromochlus* had a crurotarsal joint. Sereno (1991: p. 37) acknowledged that the calcaneum “indeed does resemble a left crurotarsal calcaneum in lateral view, but this resemblance must be misleading because the bone clearly belongs to the right tarsus.” Such reasoning was illogical because the outline of a left crurotarsal calcaneum in lateral view would not be significantly different from that of a right in medial view, and Sereno gave no justification for interpreting the exposed face of the bone as lateral rather than medial. The preserved range of motion of the ankle is ~100° from folded close to the crus to moderately extended, but there is no reason to think that it could not have been extended to nearly 180°.

The morphology of the metatarsus with its four closely appressed elongate metatarsals suggests that flexion and extension at the metatarsophalangeal joints was somehow important in locomotion with the metatarsus capable of acting as a discrete segment of the hindlimb. As such, the pes should be considered semiplantigrade (Gebo, 1992). However, the arguments of Clark et al. (1998) against digitigrady in *Dimorphodon weintraubi* would apply as well to *Scleromochlus* because the metatarsophalangeal joints do not exhibit any modifications to suggest that they were suited to a habitual digitigrade posture with the metatarsus held at ≥45° to the substrate. Digits I–IV increased in length and diverged only slightly, digit IV was ~70% of the metatarsus length, and the proximal phalanges markedly longer than the more distal (Woodward, 1907; see also Benton, 1999: fig. 13b). Such toes combined with the elongate closely appressed metatarsals would result in a long and narrow pes, unsuited to erect digitigrady.

#### Skeletal reconstruction

**Figure 20:**
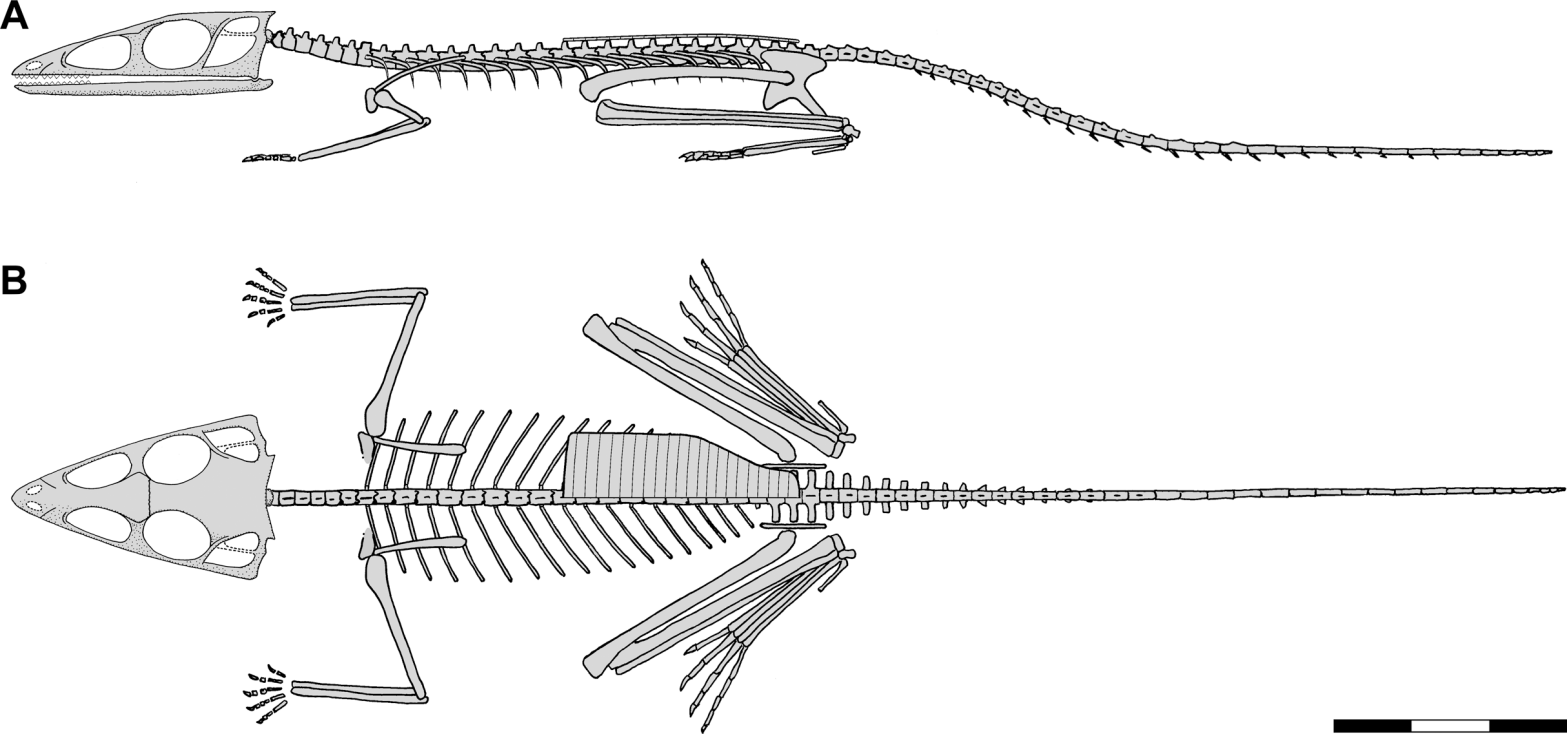
Skeletal reconstruction of *Scleromochlus taylori* in typical resting posture in dorsal (A) and left lateral (B) views. The skull and the precaudal vertebral column proportions are based on NHMUK R3146A, the pectoral girdle is based on NHMUK R3914, the manus and the pedal digits are after Woodward’s (1907) reconstruction, and the limb proportions are after Benton’s (1999: fig. 14) reconstruction. Note that the ribs, humerus, and hindlimb are foreshortened in (A). The vertical extent of the dorsal rib ends is unknown. In (B), dorsal osteoderms are shown on the right and omitted on the left to show posterior ribs. Scale bar = 3 cm.

A skeletal reconstruction of *Scleromochlus* incorporating the new evidence described and discussed above is shown in Fig. 20 (measurements in Supplemental Information). The proportions of the precaudal axial skeleton of the reconstruction were based primarily on NHMUK R3146A and caudal length was based on NHMUK R3557. The resulting proportions differ significantly from those of Sereno’s (1991: fig. 18b) and Benton’s (1999: fig. 14) reconstructions. Limb proportions were based on comparisons of NHMUK R3146A, R3914 and R3556, and are close to those of Sereno’s and Benton’s reconstructions. The skull and trunk are dorsoventrally compressed and the pelvic girdle shallow resulting in a body ~150% as wide as high, much as is found in extant lizards that spend much of their time with their trunk in contact with the substrate. The skeleton is reconstructed in what is interpreted as a typical sprawling resting posture with the brachia directed laterally, the antebrachia directed anteriorly, and the hindlimbs folded compactly and directed anterolaterally with the hip, knee, and ankle joints fully flexed. Such a posture is consistent with the reconstructed highly mobile shoulder joint, the hip joint that can be abducted in the horizontal plane but cannot swing in a parasagittal plane, the crurotarsal ankle, and the plantigrade to semiplantigrade pes. Such a posture is also consistent with the preservation of *Scleromochlus* skeletons in dorsal view with sprawling fore and hindlimb postures, articulated and undisturbed except by settling of the sand. Those who favor erect bipedal body postures such as Sereno (1991: fig. 18b) and Benton (1999: fig. 14) reconstructed should present arguments as to how the narrow ribcage and erect limbs of such erect bipeds could have been crushed into the preserved positions of the specimens.

### Locomotion

Three interpretations of the locomotion of *Scleromochlus* were proposed in early descriptions: Woodward (1907) interpreted *Scleromochlus* as a bipedal runner or jerboa-like bipedal bounder, whereas Huene (1914) interpreted it as a quadrupedal arboreal climber and leaper. Huene based his interpretation of *Scleromochlus* as an arboreal leaper on the highly mobile shoulder joint, long hindlimbs, plantigrade foot, and large claws. Padian (1984; see also Padian, Gauthier & Fraser, 1995) argued that *Scleromochlus* lacked identifiable arboreal specializations, but there is no consensus as to what features are associated with and indicators of arboreality (even goats climb trees!), so such arguments are unconvincing. Bennett (1997) accepted Huene’s interpretation and viewed *Scleromochlus* as an arboreal climber and leaper in the mode of vertical clinging and leaping primates (Napier & Walker, 1967). However, I now reject that interpretation because *Scleromochlus’*s manus and manual unguals seem to have been too small for grasping small supports or clinging to larger ones and its pedal unguals were no more curved than is typical of terrestrial animals, so it probably would not have been able to make controlled landings in trees.

Neither the Lossiemouth Sandstone nor the siltstone or claystone beneath it preserve evidence of significant numbers of trees (Benton & Walker, 1985), so there is no reason to think they provided an environment suitable for an arboreal *Scleromochlus*. In addition, if as suggested here the two individuals of NHMUK R3146 and two specimens with strong lateral flexion of the vertebral column, NHMUK R3556 and R5589, were sheltering on the ground from blowing sand, that would argue against arboreality because saltating sand grains make the ground the worst place to be in a sandstorm such that arboreal animals would surely shelter in trees.

#### Erect bipedal runner?

Woodward (1907) interpreted *Scleromochlus* as a bipedal runner but reconstructed it in a quadrupedal pose, which suggests that he viewed *Scleromochlus* as a sprawling to semi-erect facultative biped rather than as an obligate biped. However, Padian (1984) interpreted *Scleromochlus* as an obligate erect digitigrade biped, and subsequent authors (Gauthier, 1986; Sereno, 1991; Benton, 1999, 2006; Fraser, 2006) accepted that interpretation though Benton (1999: fig. 15) suggested that erect quadrupedal poses were also possible. Padian stated that *Scleromochlus* exhibited a bowed femur, fibula reduced to splint-like form, mesotarsal ankle with astragalus and calcaneum joined to the tibia, and elongate closely appressed metatarsus that implied digitigrade stance, features that formed a subset of a suite of hindlimb features that Padian (1980: 96–99; 1983), citing Coombs (1978), identified as correlates of erect digitigrade bipedal locomotion in pterosaurs (the other features were: femur shorter than tibia, double condyle knee with well developed intercondylar sulcus, median symmetry of the pes, and reduction of outer digits). However, no evidence or argumentation beyond lists of features has been offered to support that interpretation as a whole or specific aspects of it (e.g., bipedality, digitigrady, or erect limb posture), and Padian (1984) was incorrect in that the femur was not significantly bowed (and frogs also have somewhat bowed femora), the fibula was not splint-like, the ankle was not mesotarsal and the astragalus and calcaneum were not fixed to the tibia, and the ankle does not include pterosaur-like medial and lateral distal tarsals. Furthermore, no attempt has been made to explain the “high degree of specialization” that Woodward (1907: p. 144) found “truly astonishing” in what he took to be a Triassic dinosaur. Indeed, if *Scleromochlus* was an erect digitigrade bipedal runner, then its extreme cursorial adaptation remained unmatched for many million years until ornithomimid dinosaurs evolved.

Woodward (1907) implied that *Scleromochlus’*s limb proportions alone were sufficient to interpret it as a bipedal runner. Multivariate analysis of skeletal measurement data often have been used to reconstruct the locomotor or predatory behavior of fossil taxa by comparisons to extant and fossil taxa (Van Valkenburgh, 1987; Carrano, 1997, 1999; Elissamburu & Vizcaíno, 2004; Weisbecker & Warton, 2006; Shockey, Croft & Anaya, 2007; Samuels & Van Valkenburgh, 2008; Samuels, Meachen & Sakai, 2013; Janis, Buttrill & Figueirido, 2014; Janis & Figueirido, 2014; Chen & Wilson, 2015), and so a principal component analysis of trunk and limb segment lengths of *Scleromochlus* reconstructions, selected theropod dinosaurs, and representative extant vertebrates of known locomotor type was done to test Woodward’s idea.

**Figure 21:**
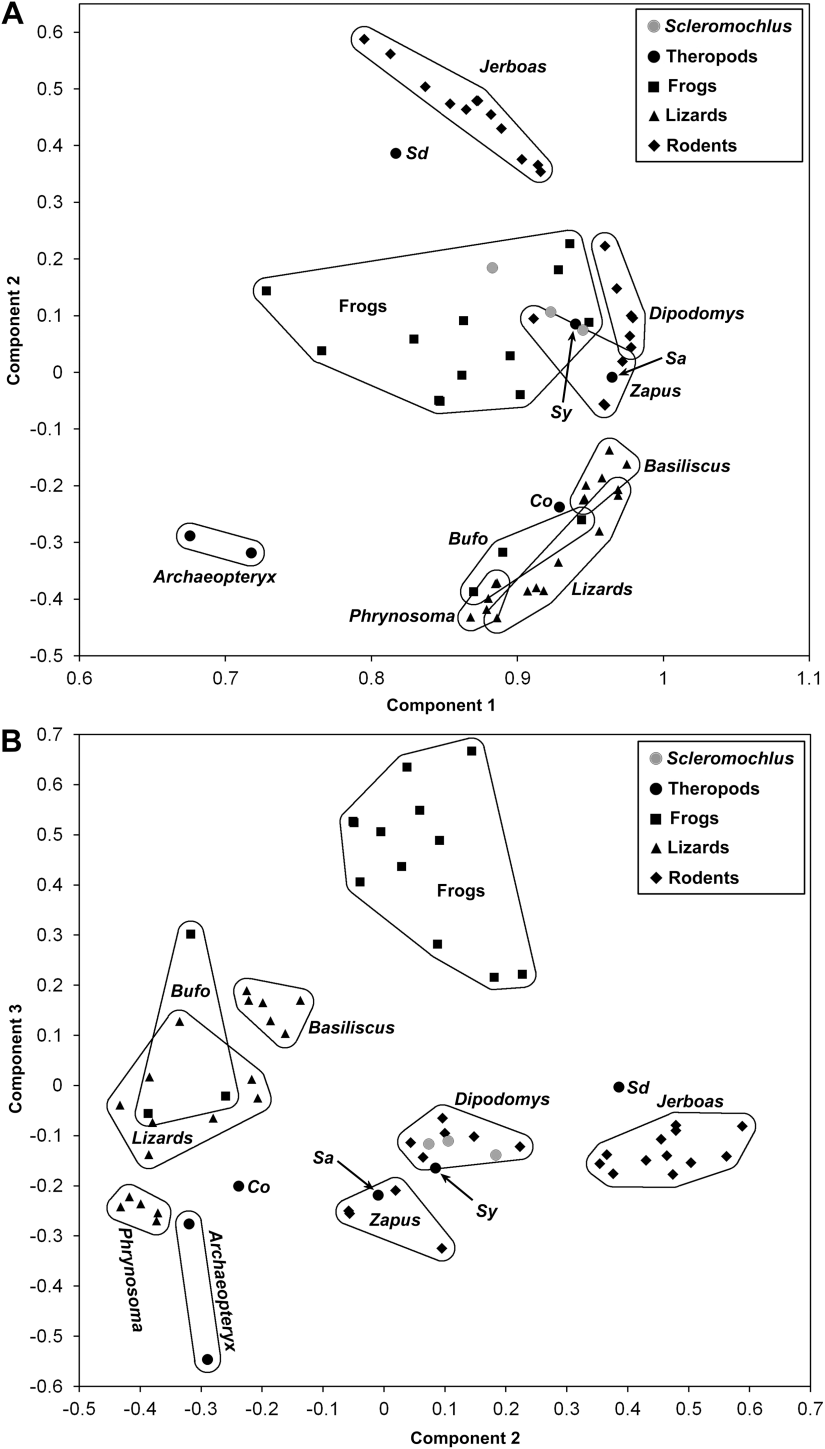
Results of the principal component analysis of limb segment and trunk lengths of *Scleromochlus taylori* reconstructions and representative vertebrates of various locomotor types. (A) Components 1 and 2; and (B) Components 2 and 3. Ar, *Archaeopteryx*; Co, *Compsognathus*; Sa, *Struthiomimus altus*; Sd, *Sinornithoides dongi*; Sy, *Sinornithomimus youngi*.

The principal component analysis resulted in three components that explain 81.4%, 8.5% and 6.8% of variance, respectively. Bivariate plots of the components with polygons drawn around the ranges of different types of vertebrates are shown in Fig. 21. In the plot of Components 1 & 2 representing 89.9% of variance, most taxa are distributed in a broad arc with jerboas at the top, *Zapus* and *Dipodomys* in the middle, and lizards and toads at the bottom, whereas the frogs stretch leftward from *Zapus* and *Dipodomys*. The theropods *Sinornithoides*, *Sinornithomimus*, *Struthiomimus* and *Compsognathus* are in a similar arc slightly to the left with *Sinornithoides* above near jerboas, *Sinornithomimus* in the middle within the range of frogs and *Struthiomimus* nearby within the range of *Zapus* and *Compsognathus* below near *Basiliscus* and *Bufo*. *Archaeopteryx* is at lower left, well away from other taxa presumably because of its long forelimbs. The *Scleromochlus* reconstructions fall within the range of frogs (near the White-lipped Tree Frog and Crawfish Frog), near the range of *Zapus*, and one is right next to *Sinornithomimus*. In the plot of Components 2 & 3 representing 15.3% of variance, frogs are upper middle, toads and lizards overlap at left, jerboas are at right, and *Scleromochlus* is within the range of *Dipodomys* with *Sinornithomimus* nearby and *Struthiomimus* is within the range of *Zapus* somewhat below.

The wide distribution of theropods and the overlap with the ranges of frogs and *Zapus* shows that limb proportions alone are not sufficient to interpret an animal as an erect bipedal runner. *Struthiomimus* (~4 m long, ~150 kg) and *Sinornithomimus* (~2.5 m, ~25 kg) are much larger than the frogs (~14 cm snout-vent length) and *Zapus* (~24 cm, ~30 gm) that they plot near, and although they share very long hindlimbs with tibia longer than femur with frogs and jumping mice, the effects of scaling are such that similar limb and trunk proportions alone in *Zapus* and the 160 times longer and 5,000 times more massive *Struthiomimus* cannot be informative (Gasc, 2001; Iriarte-Díaz, 2002; Biewener, 2005). Thus, although *Scleromochlus* plots close to *Struthiomimus* and *Sinornithomimus*, it cannot be assumed that *Scleromochlus* was an erect bipedal runner. Because *Scleromochlus* (~17 cm) was similar in size to the frogs, lizards and rodents, the fact that it does not plot close to jerboas or lizards strongly suggests that it was not an obligate bipedal bounder or a quadrupedal runner, whereas the fact that it plots close to frogs and *Zapus* suggests that its locomotion was similar to quadrupedal hopping or quadrupedal bounding.

Osteological information can be used to put the principal component analysis results in context and constrain the interpretation of locomotor type. *Scleromochlus’*s imperforate acetabulum and weakly inturned hemispherical femoral head would not have permitted the hindlimb to adopt erect postures and swing in a parasagittal plane, and even if one assumed that the hip joint morphology was not a hindrance to such movement, then the prominent tubercle on the pubis would have been. Instead, the hip morphology and preserved range of motion indicate that the hip was highly mobile and could be flexed anteriorly, extended posteriorly, and abducted laterally to a considerable degree and so was suited to sprawling to semi-erect postures. The double condyle knee suggests that *Scleromochlus* was capable of powerful knee extension, but such knees and capabilities are not limited to bipedal runners and are not necessarily inconsistent with sprawling to semi-erect postures. The crurotarsal ankle with prominent calcaneal tuber, though not necessarily mechanically unsuited to digitigrade bipedality, is typical of sprawling to semi-erect archosauriforms and is not found in erect digitigrade bipedal archosaurs. The closely appressed equilength metatarsals suggest locomotory specialization, but the metatarsophalangeal joints do not exhibit any modifications to suggest that they were suited to a habitual digitigrade posture with the metatarsus held at ≥45° to the substrate, and the asymmetrical pes with digits diverging slightly seems to have been semiplantigrade. Together these features contradict the interpretation of *Scleromochlus* as an erect digitigrade bipedal runner and suggest it was suited to sprawling to semi-erect locomotion.

There is also an evolutionary reason to think that *Scleromochlus* was not an erect digitigrade bipedal runner. Cursorial adaptations have a metabolic cost and will only be evolved if they provide selective advantage. In extant tetrapods with marked cursorial adaptations that advantage comes from improved prey acquisition and/or predator avoidance, and that presumably was the case in extinct forms as well. Cursorial adaptations usually have been coevolved by similarly sized predators and prey in escalating competition over the course of millions of years (Bakker, 1983); extant examples of such coevolution include cheetahs preying on small to medium antelopes and lions preying on large antelopes, zebras, and buffalos, and fossil examples include dromaeosaurines preying on ornithomimosaurs. Such coevolving cursorial predators and prey are similarly sized because maximum running speeds of tetrapods are roughly proportional to body size (Alexander, 1977; Weitz & Levin, 2006), so large animals do not need marked cursorial adaptations to run down or outrun significantly smaller animals and small animals even with marked cursorial adaptations cannot run down or outrun significantly larger animals. As for *Scleromochlus*, it is unlikely that extreme cursoriality would have aided in prey acquisition, for example, no extant vertebrates seem to be specialized for running down small invertebrate prey. It is also unlikely that extreme cursoriality would have aided in predator avoidance because *Erpetosuchus, Saltopus* and juvenile *Ornithosuchus* were significantly larger (≥3.5 × body length) than *Scleromochlus*. In the end, the interpretation of *Scleromochlus* as an erect digitigrade bipedal runner must be rejected because of the problems discussed above and the absence of support from the principal component analysis.

#### Erect jerboa-like bounder?

Woodward (1907), presumably prompted by C.W. Andrews’ comment in the appended discussion, proposed jerboa-like bipedal bounding as a possible alternative to bipedal running. Benton (1999, 2006) also considered it a possible alternative, whereas Benton & Walker (1985; see also Witton, 2014) argued for bounding and made no mention of running. Jerboas and kangaroo rats typically inhabit arid, sandy environments, and Benton & Walker (1985) supported their interpretation of *Scleromochlus* as a jerboa-like bounder by stating that it shared several features with desert living lizards (i.e., nares nearly closed by lateral flanges, posterior flange of squamosal and quadratojugal protecting tympanic region, countersunk mandible, and flattened metatarsals) and was adapted to and lived in a sandy dune environment. Those four features do not seem to be present in *Scleromochlus* and were not discussed by Benton (1999), but despite that he interpreted *Scleromochlus* as a possible “sand hopper.” However, as discussed above, the depositional environment provides no support for an interpretation of *Scleromochlus* as living in a sand, desert, or dune environments, and so the depositional environment cannot be used to support an interpretation of *Scleromochlus* as a jerboa-like bounder. The only remaining argument for interpreting *Scleromochlus* as a jerboa-like bounder is the marked limb length disparity and very long hindlimbs, and as discussed above the hip, ankle, and metatarsophalangeal joint morphology are unsuited to erect digitigrade bipedality with the hindlimb swinging parasagittally, whereas the hindlimb was suited to sprawling to semi-erect postures. Like erect bipedal running, the interpretation of *Scleromochlus* as a jerboa-like bounder must be rejected because of the problems discussed above and the absence of support from the principal component analysis.

#### Quadrupedal hopper

Having rejected arboreality, erect bipedal running, and jerboa-like bounding, two further alternatives remain. The sprawling quadrupedal *Scleromochlus* might be interpreted as a hopper like frogs or as a facultative bipedal runner like some lizards.

Hopping in frogs starts from a sprawling resting pose with the hindlimb fully flexed at the hip, knee, crurotarsal, and tarsometatarsal joints such that the extensor muscles are fully stretched maximizing passive tension. The extension phase begins as the body is tilted upward by extension of the forelimb because leaping at an angle of 45° above horizontal maximizes distance (Peters, Kamel & Bashor, 1996). Flexion of the glenohumeral joint may briefly assist the powerful extension of the hindlimb in accelerating the body forward and upward, but the forelimb soon clears the substrate. Full extension of the hip, knee, crurotarsal, and tarsometatarsal joints is attained just before the pedal digits clear the substrate, and the frog flies through the air with hindlimbs extended backward. Experimental removal of the tail of kangaroo rats and jumping mice results in loss of pitch control such that the animal tumbles in the air, demonstrating that their tails provide such control (Svihla & Svihla, 1933; Bartholomew & Caswell, 1951; Libby et al., 2012). Although frogs lack tails, it is likely that the long posteriorly extended hindlimbs provide pitch and yaw control in lieu of a tail. Unfortunately, experiments to demonstrate that frog hindlimbs provide such control by removal of the hindlimbs are not feasible. The recovery phase begins as the forelimbs are extended shortly before landing, and upon landing the forelimbs forcefully decelerate and stabilize the body as it pivots downward and the hindlimbs are fully flexed to return to a stable resting pose in case another hop is necessary. Note that toads, which typically have shorter hindlimbs than other frogs and so cannot hop as far, are specialized for bounding, continuous quadrupedal hopping without return to a stable resting pose (Reilly et al., 2015).

The skeleton of *Scleromochlus* was well suited to frog-like hopping. The hindlimb resembles that of frogs in having a highly mobile hip joint, long femur and somewhat longer crus, and strong knee with prominent condyles. The four closely appressed equilength metatarsals were presumably functionally equivalent to the elongate tibiale and fibulare of frogs and the elongate navicular and calcaneum in leaping *Galago* and *Tarsius*, and markedly different from the metatarsus of lizards in which metatarsals diverge and are of varying lengths. In frogs, the pes is semiplantigrade in that the elongate tibiale and fibulare are usually held slightly above the substrate when the hindlimb is fully flexed, and that may have been the case with the metatarsus of *Scleromochlus* as well. The resting pose seen in *Scleromochlus* specimens, while markedly different from that of *Leptopleuron*, resembles that of frogs in having the hindlimbs folded compactly and directed anterolaterally with the hip, knee and ankle joints fully flexed such that the extensor muscles would be maximally stretched with passive tension maximized, ready to hop if startled (Fig. 18). The strong pectoral girdle with long scapula and robust coracoid, highly mobile glenohumeral joint, and long and strong forelimb with prominent deltopectoral crest and olecranon process could tilt the body upward and change direction in anticipation of hopping, contribute to initial acceleration, and decelerate and control the body as it returned to a resting pose upon landing.

Frog hindlimbs typically are more heavily muscled than those of lizards because all the force needed for leaps of 10 body lengths or more (Zug, 1972; Gomes et al., 2009) is applied in the brief acceleration before becoming airborne (~140 ms in *Rana pipiens*, Peters, Kamel & Bashor, 1996), whereas runners apply force with every stride. Therefore, a hopping *Scleromochlus* would be expected to have significantly larger hindlimb muscles than a running one and the forces on the posterior dorsal vertebrae would be much greater than in a runner. That fact probably explains the presence of the culet of closely spaced dorsal osteoderms restricted to the posterior half of the trunk, which seems to have severely limited lateral flexion of the posterior dorsal vertebrae. The culet would have stiffened the posterior trunk, enabling it to withstand the stresses associated with the powerful extension of the hindlimbs, and it probably was functionally equivalent in that regard to the urostyle and long ilia of frogs. It has been suggested that frogs lack tails because they would limit upward body tilt and thereby distance hopped (Handrigan & Wassersug, 2007), and if frog hindlimbs provide pitch and yaw control, then a tail would add mass yet contribute nothing to hopping. However, the slender tail of *Scleromochlus* might not have significantly limited upward body tilt, and a hopping *Scleromochlus* might have retained a tail for pitch and yaw control, because *m. caudofemoralis* was an important hip extensor, or perhaps because loss would produce pleiotropic effects.

The energy costs of running locomotion in mammals are equivalent for quadrupeds and bipeds (Roberts et al., 1998), and costs of bipedal bounding in jerboas and kangaroo rats are also roughly equivalent to running though slow is less efficient and fast more efficient (Webster & Dawson, 2004). Therefore, rodents presumably did not evolve bipedal bounding to reduce energy costs. Instead, it is likely that bounding evolved for predator avoidance (Bartholomew & Caswell, 1951). Jerboas and kangaroo rats are preyed upon by larger animals (e.g., foxes) that presumably could run them down when straightaway bounding, but given their small mass and inertia jerboas and kangaroo rats can accelerate and change direction much faster than larger animals, and their erratic bounding consisting of series of powerful leaps with repeated unpredictable changes of direction aids in predator avoidance. Selection for predator avoidance could also explain the evolution of quadrupedal hopping in *Scleromochlus*. As noted above, *Scleromochlus* was presumably preyed upon by *Erpetosuchus, Saltopus* and juvenile *Ornithosuchus*, which were significantly larger (≥3.5 × body length) and would have been faster straightaway runners. However, the combination of small mass and inertia and heavily muscled hindlimbs would enable a hopping *Scleromochlus* to perform series of powerful leaps with repeated unpredictable changes of direction so as to evade the predators.

The interpretation of *Scleromochlus* as a quadrupedal hopper proposed here would require long hindlimbs and a large hindlimb muscle mass but would not require unusual muscular or physiological sophistication. It would require control of the amount and balance of force produced by the two hindlimbs during hindlimb extension to vary distance and direction, but because frogs, grasshoppers, crickets, leafhoppers, and jumping spiders can perform those tasks there is no reason to think they would be beyond the capabilities of *Scleromochlus*. In addition, the interpretation would not represent a “truly astonishing high degree of specialization,” because longer hindlimb bones and increased muscle mass presumably would be rather simple to evolve.

The other alternative interpretation is that of *Scleromochlus* as a sprawling facultative bipedal runner like extant *Basiliscus*, *Liolaemus* and *Tropidurus*, which are habitually quadrupedal but run bipedally when startled with semi-erect gait and widely spaced pedes (Rand & Marx, 1967; Glasheen & McMahon, 1996; Rocha-Barbosa et al., 2008). The widely spaced pedes provide lateral (roll) stability and their long tail provides longitudinal (pitch and yaw) stability. Although the principal component analysis did not place close to *Basiliscus*, it is possible that *Scleromochlus* was capable of sprawling facultative bipedal running; however, such an interpretation would not explain the presence of the culet of osteoderms, the closely appressed equilength metatarsals, or the frog-like resting pose in *Scleromochlu*s.

Bipedally running lizards with widely spaced pedes exhibit the same lateral undulations of the vertebral column as quadrupedal lizards and salamanders, and such undulations are desirable because they increase the stride length and speed and enable trunk musculature to contribute to locomotion. As discussed above, the culet of dorsal osteoderms would have stiffened the trunk of *Scleromochlus*, and such stiffening would be undesirable in a runner because it would prevent the lateral undulations of the vertebral column, reducing stride length and speed and preventing involvement of trunk musculature in locomotion. Bipedally running lizards have plantigrade pedes with spreading metatarsals of varying lengths, and the closely appressed equilength metatarsals of *Scleromochlus* would be unnecessary and unexpected if it was a sprawling facultative bipedal runner. The frog-like resting pose with the fully flexed hindlimb and fully stretched extensor muscles also would be unnecessary and unexpected in such a runner.

Clemente et al. (2008) found that bipedal running in lizards does not confer greater running speed or endurance compared to quadrupedally running relatives, but does result in greater initial acceleration. Perhaps longer hindlimbs have been selected for in some lizards to improve initial acceleration and so predator avoidance, and once the hindlimbs are significantly longer than the fore it is easier to run bipedally than quadrupedally. However, it seems likely that *Scleromochlus* evolved even longer and more heavily muscled hindlimbs for even greater initial acceleration and ended up flying through the air like a frog. Although the frog-like hopper and lizard-like facultative bipedal runner interpretations of *Scleromochlus* are both largely consistent with its morphology, the culet of osteoderms, closely appressed equilength metatarsals, and resting pose with fully flexed hindlimb are important components of frog-like hopping that would be disadvantageous or unnecessary in a bipedal runner, and on that basis and supported by the principal component analyses, the frog-like hopper interpretation is accepted here.

### Phylogenetic position

The phylogenetic position of *Scleromochlus taylori* was examined using Ezcurra’s (2016) 79 taxon data matrix (= his 81 taxon Analysis 3 matrix after a posteriori exclusion of *Kalisuchus rewanensis* and *Asperoris mnyama*) and Bennett’s (2013) 19 taxon 134 character Updated Data Matrix. Excurra’s analysis was used because it included basal taxa and characters necessary to resolve the relationships, which other analyses (e.g., Sereno, 1991; Benton, 1999; Brusatte et al., 2010; Nesbitt, 2011) omitted.

#### Replication of Ezcurra’s (2016) analyses

Attempts at replicating Ezcurra’s (2016) Analyses 1–3 revealed inconsistencies. The NEXUS file in his Supplemental Information was modified by the deletion of the MacClade and Mesquite commands and the addition of delete commands in the PAUP block to replicate Ezcurra’s Analyses 1–3 with 98, 97 and 81 taxa respectively, and when analyzed resulted in most parsimonious trees of 2,832, 2,948 and 2,828 steps, respectively, all markedly longer than the 2,651, 2,664 and 2,646 steps reported by Ezcurra. The PAUP block in the NEXUS file included the command “pset = mstaxa variable,” which results in the heuristic search setting “multistate taxa interpretation depends on “{}” vs. “()” designation” such that characters enclosed by braces, “{}”, are treated as uncertain and characters enclosed by parentheses, “()”, are treated as polymorphic. When the pset command was deleted from the PAUP block, then the heuristic search setting “multistate taxa interpreted as uncertainty” was applied, which resulted in most parsimonious trees of 2,651, 2,664 and 2,646 steps, respectively, the same as reported by Ezcurra. The strict consensus trees from Analyses 1 and 2 when replicated both with and without the “pset = mstaxa variable” command were the same as those in Ezcurra’s figure 52. The two treatments of multistate characters thus resulted in consensus trees with identical topologies but different lengths.

Ezcurra (2016) conducted his analyses with TNT, a program with which I am unfamiliar; however, it seems likely that if Ezcurra obtained shortest trees of 2,651, 2,664 and 2,646 from his Analyses 1–3 when using TNT, then that program was also treating all multistate characters as uncertain rather than treating those bounded by braces as uncertain and those bounded by parentheses as polymorphic.

Ezcurra (2016: pp. 271, 274) described the 81 taxon Analysis 3 as resulting in 40 most parsimonious trees of 2,646 steps and Consistency Index (CI) = 0.2978, but illustrated a strict consensus tree “after the a posteriori pruning of *Kalisuchus rewanensis* and *Asperoris mnyama”* in his Figures 48 and 50; thus the statistics do not apply to the illustrated tree. Analysis 3 when replicated with the “pset = mstaxa variable” command resulted in 1,028 most parsimonious trees of 2,828 steps and the strict consensus tree was broadly similar to that in Ezcurra’s Figures 48 and 50 but with *Asperoris mnyama, Dongusuchus efremovi, Dorosuchus neoetus, Euparkeria capensis, Yarasuchus deccanensis*, and the clade Archosauria + Proterochampsia in a polytomy, and with *Cuyosuchus huenei, Fugusuchus hejiapanensis, Kalisuchus rewanensis, Sarmatosuchus otschevi*, and the clade Erythrosuchidae + Eucrocopoda in a polytomy. Analysis 3 when replicated without the “pset = mstaxa variable” command resulted in 1008 most parsimonious trees of 2,646 steps and the same consensus tree topology.

In order to replicate the strict consensus tree in Ezcurra’s (2016) Figures 48 and 50, I added *Asperoris mnyama* and *Kalisuchus rewanensis* to the delete command in the PAUP block and repeated the analysis. This 79 taxon analysis when replicated with the command “pset = mstaxa variable” resulted in 24 most parsimonious trees of 2,821 steps and the strict consensus tree lacked the polytomies discussed above and matched the topology of the strict consensus tree in his Figures 48 and 50, whereas when replicated without the command “pset = mstaxa variable” resulted in 24 most parsimonious trees of 2,639 steps and CI of 0.2982 and the same consensus tree topology. Thus the reason for the a posteriori deletion of the two taxa was presumably cosmetic, replacing the polytomies with dichotomous branching, but why did Ezcurra not report the statistics from that analysis that produced the strict consensus tree he illustrated? In the end, it is remarkable that Ezcurra’s 79 taxon data matrix produced only 24 most parsimonious trees and a fully resolved strict consensus tree. However, it should be remembered that appearances can be deceiving and its dichotomous branching is but two taxa away from major polytomies.

#### Homogeneity testing and data matrix quality

Bull et al. (1993) showed that the probability of recovering the correct phylogeny decreases as the percent of inconsistent characters in a data set increases, and stated that data set partitioning and homogeneity testing are appropriate if one lacks confidence in a data set’s quality. Where significant homogeneity is found it is inappropriate to combine the data set partitions without modification or further justification. I lacked confidence in the quality of Ezcurra’s (2016) 79 taxon 600 character data set, so I partitioned it into Cranial, Postcranial, Forelimb and Hindlimb partitions and subjected them to homogeneity testing (see explanation in Materials and Methods). The results revealed significant incongruence in 90% of homogeneity tests at the α = 0.01 probability level (Table 4) whereas, for example, Bennett’s (2013) Revised Data Set had 0% incongruence. Although homogeneity testing may reveal incongruence related to homoplasy between one set of characters and others, just as Bennett (2013) used it to detect homoplasy in the coding of a suite of hindlimb characters that Padian (1980, 1983) asserted were associated with cursorial digitigrade bipedal locomotion in pterosaurs, here the significant incongruence in 90% of homogeneity tests indicates that there is much character inconsistency and significantly reduced probability of recovering the correct phylogeny. As a result, the data set and its phylogenetic signal can be considered to be of low quality.

**Table 4:** Results (α-values) of the homogeneity testing of four character partitions of Ezcurra’s (2016). Analysis 3 data set with a posteriori pruning of Asperoris mnyama and Kalisuchus rewanensis to 79 taxa and four character partitions of Nesbitt’s (2011) 77 taxon data set in pair-wise comparisons and individual partitions vs. all other characters based on 1,000 replicates each.

		Cranial	Postcranial	Forelimb	All others
Ezcurra (2016) analysis 3	Cranial	–			0.001*
	Postcranial	0.001*	–		0.001*
	Forelimb	0.001*	0.001*	–	0.002*
	Hindlimb	0.003*	0.001*	0.001*	0.110
Nesbitt (2011)	Cranial	–			0.001*
	Postcranial	0.001*	–		0.002*
	Forelimb	0.320	0.014	–	0.220
	Hindlimb	0.001*	0.001*	0.024	0.004*

**Note:**

* Significant at the α = 0.01 probability level.

The low quality of Ezcurra’s (2016) data set seems to result from following the general trends in recent phylogenetic analyses of increased numbers of taxa and characters (Table 5). Increasing numbers of taxa may result from increasing taxonomic breadth and/or using multiple species as terminal taxa instead of a single composite taxon to represent higher level taxa (e.g., family or order). Multiple individually coded species will usually have more missing data than a composite taxon, in which case a consequence of their use is increased missing data in the data matrix [Note that although Ezcurra (2016) and other authors used both MISSING (?) and GAP (–) symbols in their data matrices, GAP symbols are ignored in PAUP* unless DATATYPE is DNA, RNA, or PROTEIN, thus gaps are treated the same as missing]. In the case of Ezcurra’s (2016) data set, the percentage of missing data is very high, 58% in the 98 taxon matrix and 49% in the 79 taxon matrix (Table 5), and the high percentage presumably contributed to the low quality of the data set. Wiens (2003) showed that missing data could cause problems either by having insufficient characters coded for a taxon so that its position could not be accurately determined or by having insufficient taxa coded for a character so that its distribution could not be accurately determined, and it seems likely that Ezcurra’s (2016) data set suffered from both problems.

**Table 5:** Dimensions, numbers of elements, percent missing data, and consistency indices (CI) of selected data sets used to analyze the phylogenetic relationships of archosauromorphs. Gauthier’s (1984) and Benton’s (1985) analyses were done before the advent of computer analysis and published data matrices, so the number of elements was implied rather than actual. Gauthier and Benton also included additional characters and taxa to resolve lepidosauromorph relationships that are not counted here.

Analysis	Dimensions (taxa × characters)	Elements	Missing data (%)	CI
Gauthier (1984)	25 × 204	(5,100)	–	–
Benton (1985)	18 × 111	(1,998)	–	–
Sereno (1991)	9 × 36	324	12.0	0.88
Bennett (1996)	14 × 126	1,764	12.7	0.68
Benton (1999)	16 × 73	1,168	8.1	0.619
Brusatte et al. (2010)	55 × 187	10,285	35.4	0.32
Nesbitt (2011)	83 × 412	34,196	40.6	0.375
Bennett (2013), updated data set	19 × 134	2,546	18.6	0.6169
Ezcurra (2016), analysis 1	98 × 600	58,800	58.4	0.2972
Ezcurra (2016), analysis 3*	79 × 600	47,400	49.0	0.2982
Ezcurra et al. (2017)	117 × 676	79,092	56.8	0.3029^†^

**Notes:**

* With a posteriori pruning of *Asperoris mnyama* and *Kalisuchus rewanensis* to 79 taxa.

† Based on a 3543 step tree from PAUP* analysis of the NEXUS file in Ezcurra et al.’s Additional Information.

Ezcurra (2016) also followed the trend of increasing the number of characters (Table 5): Brusatte et al. (2010) had 187 characters including 47 new ones; Nesbitt (2011) had 412 characters including 114 new ones; and Ezcurra (2016) had 600 characters including 96 new ones. Comparison of the homogeneity testing of Ezcurra’s (2016) 79 taxon data matrix, in which 90% of homogeneity tests were significantly incongruent (Table 4), and Nesbitt’s (2011) 77 taxon data matrix, in which 60% of tests were significantly incongruent, suggests that the decreased quality of Ezcurra’s (2016) matrix is directly related to the 188 additional characters. However, it is probably not the number of characters, per se, but rather the quality of the added characters that affects the overall quality of the data matrix.

Because paleontologists have been compiling phylogenetic analyses to reconstruct the relationships of archosauromorphs for over 30 years, most of the good characters have already been identified. New characters whether created de novo or by atomizing old characters (i.e., splitting one character into multiple characters that code for parts of the original character) have often described simple shapes or proportions such that the derived character state may occur in multiple unrelated species. In addition, characters describing simple shapes or proportions taken from previous analyses of restricted taxonomic breadth in which they were consistent may turn out to be highly inconsistent when applied across a broader range of taxa. Such characters are phenetic, and parsimony analyses using such characters will tend to group taxa on the basis of similarities of shape and proportion rather than possession of shared unique structures inherited from common ancestors (Sneath & Sokal, 1973). Many of the characters in Ezcurra’s (2016) and Nesbitt’s (2011) analyses are phenetic with character state distributions exhibiting multiple convergent acquisitions of the derived condition, thus their analyses are hybrid phenetic-phylogenetic analyses, and both acknowledged as much: Nesbitt (2011: p. 85) stated regarding his Char. 93 “the distribution of this character is not straightforward, but it may support small clades” and Ezcurra (2016: p. 386) stated regarding his Char. 130 “this condition does not seem to have a priori a clear phylogenetic signal, but it may be informative at low taxonomic levels.” Ezcurra (2016) discussed the bimodal distribution of character CIs, but seems to have been unaware that homoplastic characters should be excluded or reformulated to reduce homoplasy as much as possible.

#### Ezcurra’s results regarding the Pterosauria

Ezcurra (2016: pp. 338–339) stated that his phylogenetic data set constituted “the best data matrix compiled so far to test the position of pterosaurs within Archosauromorpha because of the broad sample of Permo-Triassic species, including the undoubted pterosaur *Dimorphodon macronyx*.” I will grant that the taxonomic scope of Ezcurra’s data set, extending from *Petrolacosaurus* to basal dinosaurs, was the broadest at the time that included pterosaurs—Benton (1999) used *Euparkeria* as the outgroup, Brusatte et al. (2010) used *Erythrosuchus, Euparkeria* and the Proterochampsidae, Nesbitt (2011) used *Mesosuchus* and *Prolacerta* and Bennett (1996, 2013) used the Lepidosauromorpha as outgroup. However, Ezcurra’s (2016) data set seems inadequate to test the phylogenetic position of pterosaurs in that among other things: Ezcurra (2016) included only one pterosaur (Brusatte et al. used *Dimorphodon* and *Pteranodon*; Nesbitt (2011) used *Dimorphodon* and *Eudimorphodon* and Bennett’s (2013) Updated Data Set used *Anurognathus, Peteinosaurus* and *Eudimorphodon*); *Dimorphodon* was neither basal nor a typical Early Jurassic pterosaur; Ezcurra (2016) made several errors in coding *Dimorphodon*; and Ezcurra (2016) did not take the opportunity to test the relationships of *Scleromochlus taylori*, which had often been advanced as a sister taxon of the Pterosauria or of Pterosauria + Dinosauromorpha (Padian, 1984; Gauthier, 1984, 1986; Sereno, 1991; Benton, 1999, 2004; Brusatte et al., 2010).

Not only was Ezcurra’s (2016) data set not the best to test the position of pterosaurs, Ezcurra (2016) also ignored Bennett’s (2013) demonstration through data set partitioning and homogeneity testing of incongruence between a partition consisting of a suite of hindlimb characters that Padian (1980, 1983) asserted were associated with cursorial digitigrade bipedal locomotion and partitions consisting of cranial, postcranial axial, forelimb, and other hindlimb characters, respectively, and with all other characters collectively at the α = 0.01 probability level. Ezcurra (2016) ignored my further demonstration that the source of the incongruence was homoplasy in the coding of pterosaurs for the cursorial characters by removing each taxon from the data matrix one at a time and subjecting the remaining taxa to homogeneity testing. Ezcurra (2016) ignored my assertion that those homoplastic hindlimb characters had misled the earlier analyses (Gauthier, 1984, 1986; Benton, 1990; Sereno & Arcucci, 1990; Sereno, 1991) from which Bennett (1996) had drawn characters and codings, and presumably misled the subsequent analyses (Benton, 1999; Langer & Benton, 2006; Hone & Benton, 2007, 2008; Irmis et al., 2007; Nesbitt et al., 2009; Brusatte et al., 2010; Nesbitt, 2011) that also used those homoplastic characters. Ezcurra’s (2016) analysis used those homoplastic characters as well, so there is no reason not to think that it was also misled. Bennett’s (2013) interpretation of those hindlimb characters as homoplastic may or may not be correct, but until it is refuted or accepted any serious discussion of the relationships of pterosaurs to other archosauromorphs should address it.

#### Phylogenetic position of Scleromochlus

**Figure 22:**
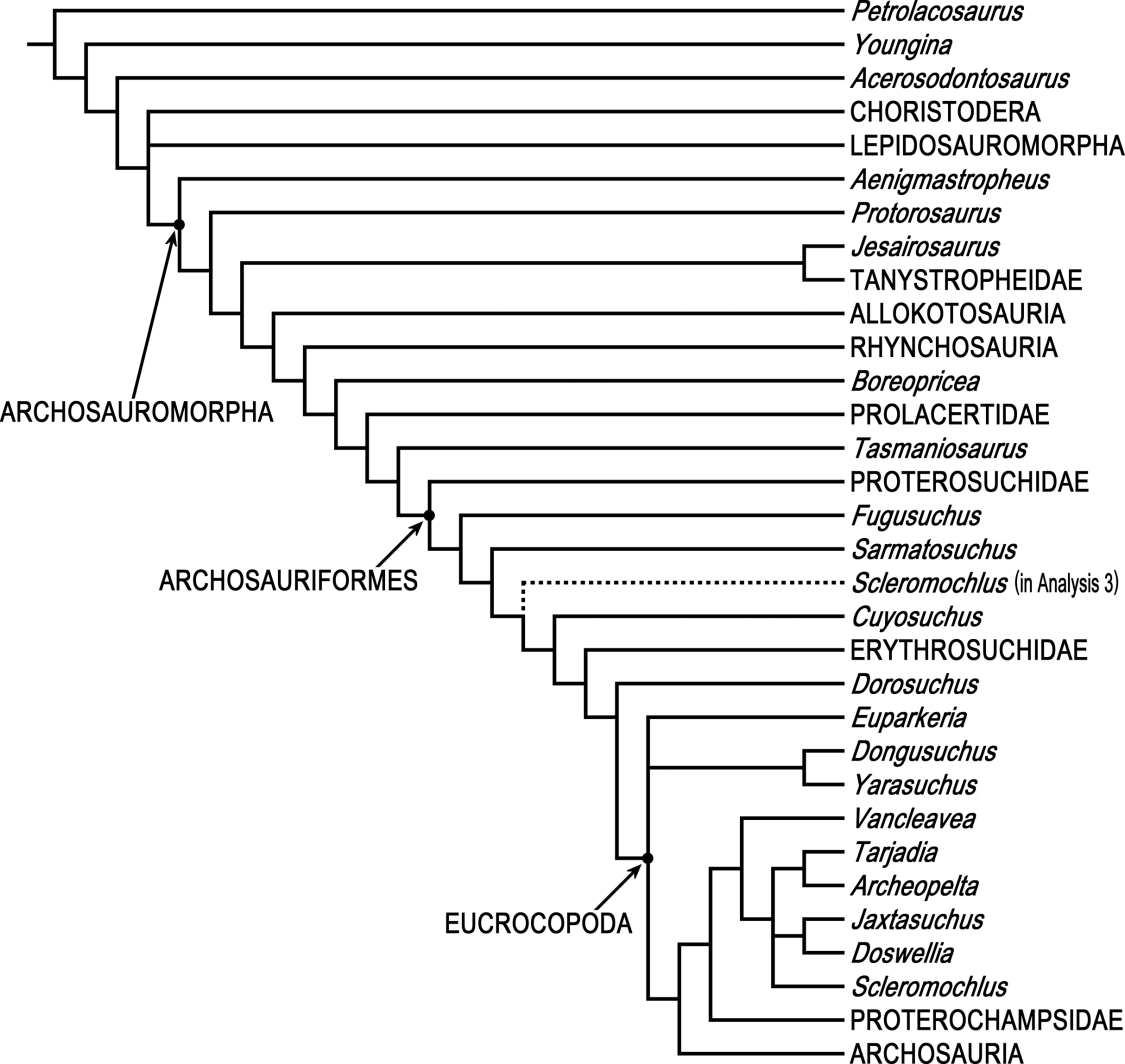
Results of parsimony analyses of the 80 taxon 600 character data set (Ezcurra’s, 2016 79 taxon data set plus *Scleromochlus taylori*). A total of 50% majority-rule consensus tree with *Scleromochlus* (3rd from bottom) in the Doswelliidae as a sister taxon to *Tarjadia ruthae* + *Archeopelta arborensis* and *Jaxtasuchus salomoni* + *Doswellia kaltenbachi*. The dashed line shows the position of *Scleromochlus* (as sister taxon of a clade equivalent to the clade Erythrosuchidae + Eucrocopoda) in the 50% majority-rule consensus tree from analysis #3 with Chars 558 and 559 set to irreversible and weighted 2, though in that analysis the relationships within the Eucrocopoda are different. Compare to Ezcurra’s (2016: figs. 48 and 50) 79 taxon strict consensus tree. Taxa at right in all capital letters are collapsed but otherwise identical in composition to those in Ezcurra’s tree.

The results of the three parsimony analyses of the 80 taxon 600 character data matrix consisting of Ezcurra’s (2016) 79 taxon data matrix plus *Scleromochlus* (Fig. 22; Supplemental Information) show *Scleromochlus* in positions other than the generally accepted sister taxon relationship with dinosauromorphs. Analysis #1 with characters ordered or unordered as described in Ezcurra’s (2016) Nexus file found 96 most parsimonious trees of 2,873 steps and CI = 0.3376. Note that all strict and 50% majority-rule consensus trees resulting from analyses #1–3 are shown in the Supplemental Information, and that in analyses #2 and 3, resulting trees were initially rooted near *Pamelaria dolichotrachela*, which made comparisons to trees from analysis #1 difficult, so *Petrolacosaurus kansensis* was designated as ancestor to make comparisons easier. The 50% majority-rule consensus tree differs from the strict consensus tree of Ezcurra’s 79 taxon analysis only in that *Scleromochlus* is a sister taxon of *Tarjadia ruthae* + *Archeopelta arborensis* and *Jaxtasuchus salomoni* + *Doswellia kaltenbachi* within the Doswelliidae, whereas the strict consensus tree has *Scleromochlus* in a polytomy with *Vancleavea campi*, *Tarjadia*, *Archeopelta* and *Jaxtasuchus* + *Doswellia*. Those topologies require the reversal of characters 558 and 559, distal tarsals 1 and 2 present or absent, respectively, and if such reversals of losses are viewed as unremarkable then we can conclude that *Scleromochlus* was a doswelliid.

Gauthier (1984) used the character “distal tarsal 1 lost and 2 slow to ossify” as a synapomorphy of the clade consisting of the most recent common ancestor of the Erythrosuchidae and crown group Archosauria and all its descendants, and Benton (1985, 1990) used the character “tarsus consists of four elements” (i.e., astragalus, calcaneum, and distal tarsals 3 and 4) as a synapomorphy of the same clade. Sereno & Arcucci (1990) stated the condition of distal tarsals 1 and 2 was unknown in erythrosuchids and used the character “distal tarsals 1 and 2 lost” as a synapomorphy of the clade *Euparkeria capensis* + Archosauria; however, Cruickshank (1978; see also Gower, 1996) showed that *Erythrosuchus africanus* had lost distal tarsals 1 and 2. Although Benton (1999) described the distal tarsals 1–4 of *Scleromochlus taylori*, his analysis used *Euparkeria* as the outgroup, his only distal tarsal characters pertained to the shape of distal tarsal 4 and its articulation with metatarsal V, and he inexplicably ignored the implication that *Scleromochlus* was either outside the clade Erythrosuchidae + Archosauria or exhibited a remarkable atavism in the reversal of the loss of distal tarsals 1 and 2. Benton (2004) commented on a 24 taxon 95 character analysis that used *Hyperodapedon* as the outgroup, but seems not to have published the data matrix. He used the characters distal tarsals 1 and 2 absent as synapomorphies of the clade *Erythrosuchus* + *Euparkeria* + Proterochampsidae + Avesuchia, which included *Scleromochlus* as the sister taxon to the clade consisting of the common ancestor of the Pterosauria and Dinosauromorpha and all its descendants, but did not comment on the reversal of the loss of distal tarsals in *Scleromochlus* that such a position presumably would have required.

The loss of distal tarsals 1 and 2 in the clade consisting of the most recent common ancestor of the Erythrosuchidae and Archosauria and all its descendants seems to be an evolutionary event equivalent to the loss of the cleithrum in primitive amniotes, manual digit V in pterosaurs, and all teeth in birds, that is, complete loss of a skeletal structure or structures and modification of the adjacent skeletal elements to adapt to and compensate for the loss. There are no known instances of reversals of the losses of the cleithrum, manual digit V, or teeth, and no known instances of reversals of the loss of distal tarsals 1 and 2 in archosauriforms, so it is remarkable that anyone would think it no problem evolutionarily to reverse the loss of distal tarsals 1 and 2 in *Scleromochlus* simply because it is most parsimonious in a cladistic analysis. Until an explanation for reversal of the loss of distal tarsals 1 and 2 in *Scleromochlus* can be found, it is reasonable to assume that it is evolutionarily most likely that the presence of distal tarsals 1 and 2 in *Scleromochlus* was not the result of the reversal of losses.

In order to explore alternatives to the result of Analysis #1, a second analysis was done with Chars. 558 and 559 set to irreversible. Analysis #2 found >1,000 most parsimonious trees of 2879 steps and CI = 0.3369. The strict and 50% majority-rule consensus trees from analysis #2 are identical to those of analysis #1 with *Scleromochlus* within the Doswelliidae, and the greater length reflects multiple convergent losses of distal tarsals 1 and 2 in other taxa instead of the above noted reversals of the losses in *Scleromochlus*. However, that also seems unlikely because such a result would require the retention of distal tarsals 1 and 2 in *Scleromochlus* while multiple closely related lineages underwent convergent losses of the tarsals.

A third analysis was done with Chars. 558 and 559 set to irreversible and weighted as 2 in order to reduce the parsimoniousness of convergent losses of the distal tarsals. Analysis #3 found >1000 most parsimonious trees of 2,886 steps and CI = 0.3369. The 50% majority-rule consensus tree has *Scleromochlus* as a sister taxon of a clade consisting of the common ancestor of *Euparkeria*, Erythrosuchidae, Proterochampsia and Archosauria and all its descendants, in a position that is as far up the tree as possible without multiple convergent losses of the distal tarsals in related taxa (see dashed line in Fig. 22). Note that although the composition of that clade is equivalent to the clade Erythrosuchidae + Eucrocopoda, the ingroup relationships are different from those in Ezcurra’s 79 taxon analysis. The strict consensus tree has *Scleromochlus* in the same position and most taxa within the Eucrocopoda in a large polytomy.

Although analyses #1–3 do not seem to suggest exactly where in the archosauriform tree *Scleromochlus* belongs, the most important message from the analyses is that there is no support in any of them for a placement of *Scleromochlus* as sister taxon of either the Pterosauria or Dinosauromorpha (Sereno, 1991) or as sister taxon to the Pterosauria + Dinosauromorpha (Benton, 1999, 2004) or as a sister taxon in a tritomy with *Lagerpeton* and the Dinosauriformes (Ezcurra et al., 2017). In the end, it must be concluded that *Scleromochlus* was not close to dinosauromorphs.

In analyses #1–3, the pterosaur *Dimorphodon macronyx* was placed as sister taxon of the Dinosauromorpha. Although Ezcurra (2016) opined that his analysis was the best data matrix compiled so far to test the position of pterosaurs within the Archosauromorpha, there are errors in the coding of *Dimorphodon* and other problems discussed above. As such, neither Ezcurra’s (2016) analysis nor the three 80 taxon analyses should be viewed as providing significant support for the Pterosauria as a sister taxon of the Dinosauromorpha (Padian, 1984; Gauthier, 1984, 1986; Sereno, 1991; Benton, 1999, 2004; Brusatte et al., 2010; Nesbitt, 2011).

Bennett (1996) argued that a suite of hindlimb characters that Padian (1980, 1983) asserted were associated with cursorial digitigrade bipedal locomotion in pterosaurs were convergent with those of dinosaurs; however, I used a posteriori recoding of a set of functionally related characters, which was deemed an inappropriate methodology and gave others an excuse to ignore the conclusion that pterosaurs were not ornithodirans. It is notable that Jiang et al. (2016) did the same thing, a posteriori recoding of a set of functionally related characters in order to examine their effect on the evolution of ichthyosauriforms, but their methods and results have not been challenged. However, Bennett (2013) demonstrated through homogeneity testing of Bennett’s (1996) data set after division into five partitions, that the partition consisting of the suite of characters that Padian (1980, 1983) asserted were associated with cursorial digitigrade bipedal locomotion was incongruent with other partitions and all other characters at the α = 0.01 probability level. To date, no one has suggested that Bennett (2013) used inappropriate methodology, but the conclusions have been ignored.

**Figure 23:**
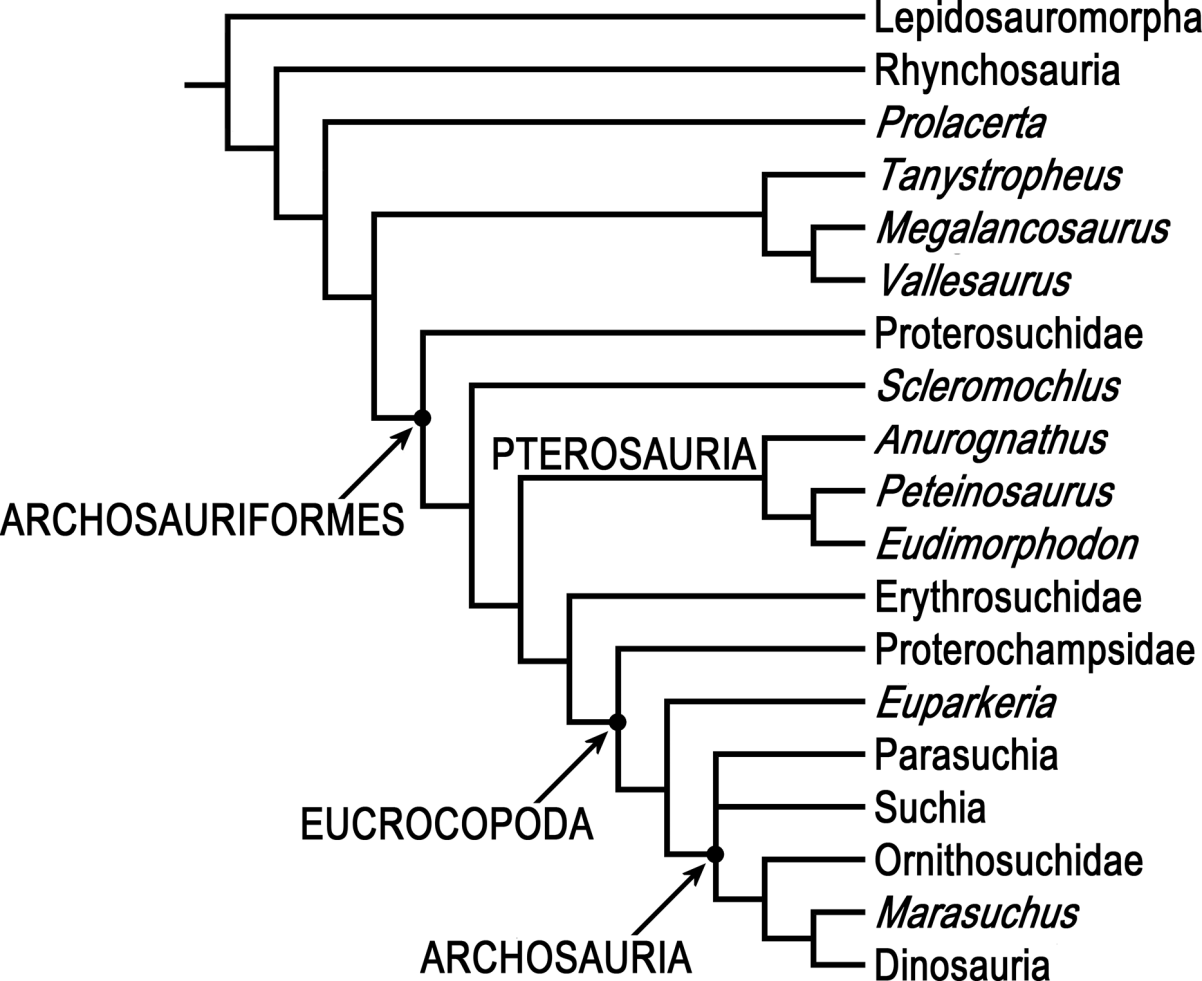
Single most parsimonious tree resulting from the parsimony analysis of Bennett’s (2013) 19 taxon 134 character Updated Data Matrix, etc. Single most parsimonious tree resulting from the parsimony analysis of Bennett’s (2013) 19 taxon 134 character Updated Data Matrix with the coding of *Scleromochlus taylori* corrected on the basis of the new information and interpretation in the present article, with *Scleromochlus* as sister taxon of the Pterosauria + (Erythrosuchidae + Eucrocopoda).

The character codings for *Scleromochlus* used in the 1996 and 2013 cladistic analyses were based on my uncritical acceptance of Padian’s (1984) interpretation of *Scleromochlus’*s hindlimb morphology, Gauthier’s (1984, 1986) and Sereno’s (1991) codings of *Scleromochlus* from their cladistic analyses, and Huene’s (1914) interpretation of *Scleromochlu*s as an arboreal leaper rather than on examinations of the specimens or casts, so the codings needed to be corrected. Analysis of Bennett’s (2013) 19 taxa 134 character Updated Data Matrix with the coding of *Scleromochlus* corrected on the basis of the new information and interpretation in the present article found a single most parsimonious tree of 274 steps and CI = 0.5876 (Fig. 23), which differs from that of Bennett (2013: fig. 3) in that *Scleromochlus* is the sister taxon of the clade Pterosauria + (Erythrosuchidae + Eucrocopoda). That result compares well with the results of Analyses #1–3 of the 80 taxon 600 character data matrix discussed above in that *Scleromochlus* is not an ornithodiran, and compares well with Analysis #3 in that *Scleromochlus* is outside the clade Erythrosuchidae + Eucrocopoda. Although *Scleromochlus* is close to the Pterosauria in the cladogram, its crurotarsal ankle and terrestrial lifestyle suggest that it was not particularly close to the last non-volant ancestor origin of pterosaurs.

## Conclusions

Several aspects of the osteology of *Scleromochlus taylori* have been reinterpreted. Evidence and arguments have been presented that the trunk was dorsoventrally compressed with the ribs directed laterally to form a broad flat-topped ribcage, broad closely spaced dorsal osteoderms formed a culet covering the posterior trunk, the coracoid was larger and more robust than generally thought, the imperforate acetabulum and only weakly inflected hemispherical femoral head would not have permitted erect hindlimb postures, and the calcaneum with prominent calcaneal tuber, crurotarsal ankle, and asymmetrical plantigrade to semiplantigrade pes with only moderately spreading digits are consistent with sprawling postures. New reconstructions of the skull and skeleton have been presented, and *Scleromochlus* has been interpreted as a sprawling quadrupedal animal with extremely long hindlimbs adapted for frog-like hopping locomotion to evade predation, an adaptation that is unique among diapsids. The principal component analysis does not provide support for the interpretation of *Scleromochlus* as an erect digitigrade bipedal cursor, and the cladistic analyses demonstrated at a minimum that *Scleromochlus* was not an ornithodiran.

Although the generally accepted interpretation of *Scleromochlus* as a digitigrade bipedal cursor close to dinosauromorphs has been rejected, I have agreed with many aspects of previous interpretations. For example, in regard to the ankle of *Scleromochlus*, I agreed with Benton (1999) that four distal tarsals were present such that the two larger tarsals were the proximal astragalus and calcaneum, with Padian’s (1984) demonstration that the medial proximal tarsal was significantly larger than the lateral, with Sereno’s (1991) acceptance that the larger tarsal was medial and that the smaller lateral tarsal resembled a crurotarsal calcaneum, and with Woodward (1907), Huene (1914) and Martin (1983) that the smaller lateral tarsal was the calcaneum with a prominent tuber and the ankle was crurotarsal. I have also agreed with Benton (1999: figs. 4 and 6) that *Scleromochlus* had closely spaced broad dorsal body osteoderms. The differences from the generally accepted view of *Scleromochlus* came from analyses that previous workers had not done. Whereas previous interpretations of the locomotion of *Scleromochlus* were based at least in part on the notion that very long hindlimbs were indicative of bipedal running, the principal component analysis did not support that view because *Scleromochlus* plotted within the range of frogs and close to the quadrupedal bounder *Zapus*. Whereas previous phylogenetic analyses that supported *Scleromochlus* as a sister taxon to dinosauromorphs were of restricted taxonomic breadth with *Euparkeria* as the outgroup (e.g., Sereno, 1991; Benton, 1999) and/or omitted characters related to *Scleromochlus’*s osteoderms, crurotarsal ankle, and retention of distal tarsals 1 and 2 (e.g., Ezcurra et al., 2017) combining Ezcurra’s (2016) taxonomically broad 600 character 79 taxon data matrix with new coding of *Scleromochlus* for those characters, etc., resulted in an analysis that found that *Scleromochlus* was not close to dinosauromorphs.

## Supplemental Information

10.7717/peerj.8418/supp-1 Supplemental Information 1 Bennett *Scleromochlus* Supplementary Information.

10.7717/peerj.8418/supp-2 Supplemental Information 2 Nexus file for analysis of Ezcurra’s (2016) 79 taxa with *Scleromochlus* added.

10.7717/peerj.8418/supp-3 Supplemental Information 3 Nexus file for analysis of Bennett’s (2013) Updated Data Matrix with *Scleromochlus* corrected.

10.7717/peerj.8418/supp-4 Supplemental Information 4 Measurements of Scleromochlus and representative vertebrates of known locomotor type.

## Institutional abbreviations

AMNH: American Museum of Natural History, New York, NY, USA
BSP: Bayerische Staatssammlung für Paläontologie und Geologie, Munich, Germany
FHSM: Fort Hays Sternberg Museum, Herpetology Collection, Hays, KS, USA
HMN: Humboldt Museum fur Naturkunde, Berlin, Germany
IVPP: Institute of Vertebrate Paleontology and Paleoanthropology, Beijing, China
JM: Jura Museum, Eichstätt, Germany
KU: Divisions of Mammalogy and Herpetology and KUVP, Division of Vertebrate Paleontology, University of Kansas Natural History Museum, Lawrence, KS, USA
NHMUK: Natural History Museum, London, England (formerly BMNH)
NMNH: National Museum of Natural History, Division of Mammals, Washington, DC, USA
NMS: National Museums Scotland, Edinburgh, Scotland (formerly RSM)
UCRC: University of Chicago Research Collection, University of Chicago, Chicago, IL, USA
USMN: United States National Museum, Division of Mammals, Washington, DC, USA
YPM: Peabody Museum of Natural History, Yale University, New Haven, CT, USA

## Additional Information and Declarations

### Data Availability

The following information was supplied regarding data availability: The measurement data used in the principal component analysis including catalog numbers for all specimens and the Nexus files used in the PAUP analysis are available in the Supplemental Files. The repositories of the specimens are available in the Institutional Abbreviations section above.
