# Supplementary material for: Reassessment of the Triassic archosauriform Scleromochlus taylori: neither runner nor biped, but hopper
Source: PeerJ. 2020 Feb 19;8:e8418. doi: 10.7717/peerj.8418 (PMC7035874; doi:10.7717/peerj.8418)
Supplement: Supplemental Information 1 [file peerj-08-8418-s001.pdf]

+

OUTOUT CONSENSUS TREES FROM BENNETT'S PAUP* ANALYSIS OF EZCURRA'S 79 TAXON DATA MATRIX PLUS *SCLEROMOCHLUS*

Analysis #1

```
Strict consensus of 96 trees:

/------------------------------------------------------------ Petrolacosaurus
|
| /---------------------------------------------------------- Acerosodontosaur
| |
| |                                                     /---- Paliguana whitei
| |                                                     |
| | /---------------------------------------------------+ /-- Planocephalosaur
| | |                                                   \-+
+-+ |                                                     \-- Gephyrosaurus br
| | |
| | |                                                     /-- Ctenio genys sp.
| | +-----------------------------------------------------+
| | |                                                     \-- Simoedosaurus le
| | |
| \-+  /----------------------------------------------------- Aenigmastropheus
|   |  |
|   |  | /--------------------------------------------------- Protorosaurus sp
|   |  | |
|   |  | |                                                /-- Amotosaurus rotf
|   |  | |                                              /-+
|   |  | |                                              | \-- Tanystropheus lo
|   \--+ |                                           /--+
|      | |                                           |  \---- Macrocnemus bass
|      | | /-----------------------------------------+
|      | | |                                         \------- Jesairosaurus le
|      | | |
|      | | |                                            /---- Pamelaria dolich
|      \-+ |                                            |
|        | | /------------------------------------------+ /-- Azendohsaurus ma
|        | | |                                          \-+
|        | | |                                            \-- Trilophosaurus b
|        | | |
|        | | |                                   /----------- Noteosuchus coll
|        | | |                                   |
|        | | |  /--------------------------------+ /--------- Mesosuchus brown
|        | | |  |                                | |
|        | | |  |                                \-+ /------- Howesia browni
|        | | |  |                                  | |
|        \-+ |  |                                  \-+  /---- Eohyosaurus wolv
|          | |  |                                    |  |
|          | |  |                                    \--+ /-- Rhynchosaurus ar
|          | |  |                                       \-+
|          | |  |                                         \-- Bentonyx sidensi
|          | |  |
|          | |  |                                         /-- Prolacerta broom
|          | |  |   /-------------------------------------+
|          | |  |   |                                     \-- K australiensis
|          | |  |   |
|          | |  |   |                                /------- Proterosuchus fe
|          | |  |   |                                |
|          | |  |   |                                |    /-- Proterosuchus go
|          | |  |   |   /----------------------------+  /-+
|          | |  |   |   |                            |  | \-- 'Chasmatosaurus'
```

```
+

|          \-+  |   |   |                            \--+
|            |  |   |   |                               \---- Proterosuchus al
|            |  |   |   |
|            |  |   |   |                          /--------- Koilamasuchus go
|            |  |   |   |                          |
|            |  |   |   |                          |      /-- Youngosuchus sin
|            |  |   |   |                          |    /-+
|            |  |   |   |                          |    | \-- Batrachotomus ku
|            |  |   |   |                          | /--+
|            |  |   |   |                        /-+ |  \---- Prestosuchus chi
|            |  |   |   |                        | +-+
|            |  |   |   |                        | | |    /-- Turfanosuchus da
|            |  |   |   |                        | | \----+
|            |  |   |   |                        | |      \-- Gracilisuchus st
|            |  |   |   |                      /-+ |
|            |  |   |   |                      | | \--------- Aetosauroides sc
|            |  |   |   |                      | |
|            |  |   |   |                      | |        /-- Ornithosuchus lo
|            |  |   |   |                   /--+ \--------+
|            |  |   |   |                   |  |          \-- Riojasuchus tenu
|            |  |   |   |                   |  |
|            |  |   |   |                   |  \------------- Nundasuchus song
|            |  |   |   |                 /-+
|            |  |   |   |                 | |             /-- Parasuchus angus
|            |  |   |   |                 | |           /-+
|            \--+   |   |                 | |           | \-- Parasuchus hislo
|               |   |   |                 | \-----------+
|               |   |   |                 |             | /-- Nicrosaurus kapf
|               |   |   |               /-+             \-+
|               |   |   |               | |               \-- Smilosuchus spp.
|               |   |   |               | |
|               |   |   |               | |      /----------- Dimorphodon macr
|               |   |   |               | |      |
|               | /-+   |               | |      | /--------- Lagerpeton chana
|               | | |   |               | \------+ |
|               | | |   |               |        | | /------- Marasuchus lillo
|               | | |   |               |        \-+ |
|               | | |   |               |          | |    /-- Lewisuchus admix
|               | | |   |               |          \-+  /-+
|               | | |   |               |            |  | \-- Silesaurus opole
|               | | |   |               |            \--+
|               | | |   |               |               | /-- Heterodontosauru
|               | | |   |               |               \-+
|               | | |   |             /-+                 \-- Herrerasaurus is
|               | | |   |             | |
|               | | |   |             | |               /---- Vancleavea campi
|               | | |   |             | |               |
|               | | | /-+             | |               +---- Archeopelta arbo
|               | | | | |             | |               |
|               | | | | |             | |               +---- Tarjadia ruthae
|               | | | | |             | |   /-----------+
|               | | | | |             | |   |           | /-- Jaxtasuchus salo
|               | | | | |             | |   |           +-+
|               | | | | |             | |   |           | \-- Doswellia kalten
|               | | | | |             | |   |           |
|               | | | | |             | |   |           \---- Scleromochlus ta
|               | | | | |             | |   |
|               | | | | |             | \---+             /-- Proterochampsa b
|               | | | | |             |     |  /----------+
|               | | | | |             |     |  |          \-- Proterochampsa n
|               | | | | |           /-+     |  |
|               | | | | |           | |     |  |   /--------- Tropidosuchus ro
|               | | | | |           | |     |  |   |
|               | | | | |           | |     \--+   |    /---- Gualosuchus reig
```

+

```
|               | | | | |           | |        | /-+    |
|               | | | | |           | |        | | | /--+ /-- Chanaresuchus bo
|               | | | | |           | |        | | | |  \-+
|               | | | | |           | |        | | \-+    \-- Rhadinosuchus gr
|               \-+ | | |           | |        \-+   |
|                 | | | |           | |          |   \------- Pseudochampsa is
|                 | | | |        /--+ |          |
|                 | | | |        |  | |          \----------- Cerritosaurus bi
|                 | | | |        |  | |
|                 | | | |        |  | +---------------------- Euparkeria capen
|                 | \-+ |        |  | |
|                 |   | |        |  | |                   /-- Yarasuchus decca
|                 |   | |        |  | \-------------------+
|                 |   | |        |  |                     \-- Dongusuchus efre
|                 |   | |      /-+  |
|                 |   | |      | |  \------------------------ Dorosuchus neoet
|                 |   | |      | |
|                 |   | |      | |                 /--------- Guchengosuchus s
|                 |   | |      | |                 |
|                 |   | |      | |                 |      /-- GHG 7433MI
|                 |   | |      | |                 |      |
|                 |   | |      | \-----------------+ /----+-- Garjainia prima
|                 |   | |      |                   | |    |
|                 |   | |    /-+                   | |    \-- Garjainia madiba
|                 |   | |    | |                   \-+
|                 |   | |    | |                     |  /---- Erythrosuchus af
|                 |   | |    | |                     |  |
|                 |   | |    | |                     \--+ /-- Shansisuchus sha
|                 |   | |  /-+ |                        \-+
|                 |   | |  | | |                          \-- Chalishevia coth
|                 |   | |  | | |
|                 |   | |  | | \----------------------------- Cuyosuchus huene
|                 |   | \--+ |
|                 |   |    | \------------------------------- Sarmatosuchus ot
|                 |   |    |
|                 |   |    \--------------------------------- Fugusuchus hejia
|                 |   |
|                 |   \-------------------------------------- Tasmaniosaurus t
|                 |
|                 \------------------------------------------ Boreopricea fune
|
\------------------------------------------------------------ Youngina capensi
```

50% Majority-rule consensus of 96 trees

```
/------------------------------------------------------- Petrolacosaurus (1)
|
| /----------------------------------------------------- Acerosodontosaur(2)
| |
| |                                                /---- Paliguana whitei(4)
| |                                                |
| | /---------------------100----------------------+ /-- Planocephalosaur(5)
| | |                                              100
100 |                                                \-- Gephyrosaurus br(6)
| | |
| | |                                                /-- Cteniogenys sp.(7)
| | +----------------------100-----------------------+
| | |                                                \-- Simoedosaurus le(8)
| | |
| 100 /------------------------------------------------- Aenigmastropheus(9)
|   | |
|   | | /----------------------------------------------- Protorosaurus sp(10)
|   | | |
|   | | |                                            /-- Amotosaurus rotf(11)
```

+

```
|   | | |                                          100
|   | | |                                          | \-- Tanystropheus lo(13)
|   100 |                                        100
|     | |                                        | \---- Macrocnemus bass(12)
|     | | /-----------------100------------------+
|     | | |                                      \------ Jesairosaurus le(14)
|     | | |
|     | | |                                        /---- Pamelaria dolich(15)
|     100 |                                        |
|       | | /-----------------100------------------+ /-- Azendohsaurus ma(16)
|       | | |                                      100
|       | | |                                        \-- Trilophosaurus b(17)
|       | | |
|       | | |                                /---------- Noteosuchus coll(18)
|       | | |                                |
|       | | | /-------------100--------------+ /-------- Mesosuchus brown(19)
|       | | | |                              | |
|       | | | |                              100 /------ Howesia browni(20)
|       | | | |                                | |
|       100 | |                                100 /---- Eohyosaurus wolv(21)
|         | | |                                  | |
|         | | |                                  100 /-- Rhynchosaurus ar(22)
|         | | |                                    100
|         | | |                                      \-- Bentonyx sidensi(23)
|         | | |
|         | | |                                      /-- Prolacerta broom(26)
|         | | |   /---------------100----------------+
|         | | |   |                                  \-- K australiensis (28)
|         | | |   |
|         | | |   |                              /------ Proterosuchus fe(32)
|         | | |   |                              |
|         | | |   |                              |   /-- Proterosuchus go(33)
|         | | |   |   /-----------100------------+ 100
|         | | |   |   |                          | | \-- 'Chasmatosaurus'(35)
|         100 |   |   |                          100
|           | |   |   |                            \---- Proterosuchus al(34)
|           | |   |   |
|           | |   |   |                        /-------- Koilamasuchus go(48)
|           | |   |   |                        |
|           | |   |   |                        |     /-- Youngosuchus sin(62)
|           | |   |   |                        |   100
|           | |   |   |                        |   | \-- Batrachotomus ku(94)
|           | |   |   |                        | 100
|           | |   |   |                      100 | \---- Prestosuchus chi(95)
|           | |   |   |                      | 100
|           | |   |   |                      | | |   /-- Turfanosuchus da(91)
|           | |   |   |                      | | \100+
|           | |   |   |                      | |     \-- Gracilisuchus st(92)
|           | |   |   |                    100 |
|           | |   |   |                    | | \-------- Aetosauroides sc(93)
|           | |   |   |                    | |
|           | |   |   |                    | |       /-- Ornithosuchus lo(88)
|           | |   |   |                  100 \--100--+
|           | |   |   |                  | |         \-- Riojasuchus tenu(89)
|           | |   |   |                  | |
|           | |   |   |                  | \------------ Nundasuchus song(90)
|           | |   |   |                100
|           | |   |   |                | |           /-- Parasuchus angus(84)
|           | |   |   |                | |         100
|           100   |   |                | |         | \-- Parasuchus hislo(85)
|             |   |   |                | \---100---+
|             |   |   |                |           | /-- Nicrosaurus kapf(86)
|             |   |   |              100           100
|             |   |   |              | |             \-- Smilosuchus spp.(87)
```

```
+

|             |   |   |              | |
|             |   |   |              | |     /---------- Dimorphodon macr(96)
|             |   |   |              | |     |
|             | 100   |              | |     | /-------- Lagerpeton chana(97)
|             | | |   |              | \-100-+ |
|             | | |   |              |       | | /------ Marasuchus lillo(98)
|             | | |   |              |       100 |
|             | | |   |              |         | |   /-- Lewisuchus admix(99)
|             | | |   |              |         100 100
|             | | |   |              |           | | \-- Silesaurus opole(100)
|             | | |   |              |           100
|             | | |   |              |             | /-- Heterodontosauru(101)
|             | | |   |            100             100
|             | | |   |            | |               \-- Herrerasaurus is(102)
|             | | |   |            | |
|             | | |   |            | |           /------ Vancleavea campi(66)
|             | | |   |            | |           |
|             | | | 100            | |           |   /-- Archeopelta arbo(80)
|             | | | | |            | |           | 75+
|             | | | | |            | |   /--100--+ | \-- Tarjadia ruthae(81)
|             | | | | |            | |   |       | |
|             | | | | |            | |   |       | | /-- Jaxtasuchus salo(82)
|             | | | | |            | |   |       75100
|             | | | | |            | |   |         | \-- Doswellia kalten(83)
|             | | | | |            | |   |         |
|             | | | | |            | |   |         \---- Scleromochlus ta(103)
|             | | | | |            | \100+
|             | | | | |            |     |           /-- Proterochampsa b(72)
|             | | | | |          75+     | /---100---+
|             | | | | |          | |     | |         \-- Proterochampsa n(73)
|             | | | | |          | |     | |
|             | | | | |          | |     | |   /-------- Tropidosuchus ro(74)
|             | | | | |          | |     | |   |
|             | | | | |          | |     100   |   /---- Gualosuchus reig(76)
|             | | | | |          | |       | 100   |
|             | | | | |          | |       | | | 100 /-- Chanaresuchus bo(77)
|             | | | | |          | |       | | | | 100
|             | | | | |          | |       | | 100   \-- Rhadinosuchus gr(79)
|            100 | | |        100 |       100   |
|               | | | |        | | |         |   \------ Pseudochampsa is(78)
|               | | | |        | | |         |
|               | | | |        | | |         \---------- Cerritosaurus bi(75)
|               | | | |        | | |
|               | | | |        | | |                 /-- Yarasuchus decca(70)
|               | 100 |      100 | \-------100-------+
|               |   | |      | | |                   \-- Dongusuchus efre(71)
|               |   | |      | | |
|               |   | |      | | \---------------------- Euparkeria capen(68)
|               |   | |      | |
|               |   | |      | \------------------------ Dorosuchus neoet(69)
|               |   | |    100
|               |   | |    | |                 /-------- Guchengosuchus s(52)
|               |   | |    | |                 |
|               |   | |    | |                 |     /-- GHG 7433MI(54)
|               |   | |    | |                 |     |
|               |   | |    | \-------100-------+ /100+-- Garjainia prima(55)
|               |   | |    |                   | |   |
|               |   | |    |                   | |   \-- Garjainia madiba(57)
|               |   | |  100                 100
|               |   | |   ||                   | /---- Erythrosuchus af(58)
|               |   | |   ||                   | |
|               |   | |   ||                   100 /-- Shansisuchus sha(59)
|               |   | |   ||                     100
|               |   | | 100|                         \-- Chalishevia coth(61)
```

```
+

|               |   | | | ||
|               |   | | | |\---------------------------- Cuyosuchus huene(53)
|               |   | 100 |
|               |   |   | \----------------------------- Sarmatosuchus ot(51)
|               |   |   |
|               |   |   \------------------------------- Fugusuchus hejia(50)
|               |   |
|               |   \----------------------------------- Tasmaniosaurus t(38)
|               |
|               \--------------------------------------- Boreopricea fune(29)
|
\------------------------------------------------------- Youngina capensi(3)
```

Analysis #2 with Chars 558 & 559 Irreversible

```
Strict consensus of 1000 trees:

/---------------------------------------------------------- Petrolacosaurus
|
|   /------------------------------------------------------ Acerosodontosaur
|   |
|   |                                                 /---- Paliguana whitei
|   |                                                 |
|   | /-----------------------------------------------+ /-- Planocephalosaur
|   | |                                               \-+
| /-+ |                                                 \-- Gephyrosaurus br
| | | |
| | | |                                                 /-- Cteniogenys sp.
| | | +-------------------------------------------------+
| | | |                                                 \-- Simoedosaurus le
| | | |
| | \-+ /-------------------------------------------------- Aenigmastropheus
| |   | |
| |   | | /------------------------------------------------ Protorosaurus sp
| |   | | |
| |   | | |                                             /-- Amotosaurus rotf
| |   | | |                                           /-+
| |   | | |                                           | \-- Tanystropheus lo
| |   \-+ |                                         /-+
| |     | |                                         | \---- Macrocnemus bass
| |     | | /---------------------------------------+
| |     | | |                                       \------ Jesairosaurus le
| |     | | |
| |     | | |                                         /---- Pamelaria dolich
| |     \-+ |                                         |
| |       | |  /--------------------------------------+ /-- Azendohsaurus ma
| |       | |  |                                      \-+
| |       | |  |                                        \-- Trilophosaurus b
| |       | |  |
| |       | |  |                                /---------- Noteosuchus coll
| |       | |  |                                |
| |       | |  | /------------------------------+ /-------- Mesosuchus brown
| |       | |  | |                              | |
| |       | |  | |                              \-+ /------ Howesia browni
| |       | |  | |                                | |
| |       \-+  | |                                \-+ /---- Eohyosaurus wolv
| |         |  | |                                  | |
| |         |  | |                                  \-+ /-- Rhynchosaurus ar
```

+

```
  | |         |  | |                                     \-+
--+ |         |  | |                                       \-- Bentonyx sidensi
  | |         |  | |
  | |         |  | |                                       /-- Prolacerta broom
  | |         |  | |   /-----------------------------------+
  | |         |  | |   |                                   \-- K australiensis
  | |         |  | |   |
  | |         |  | |   |                               /------ Proterosuchus fe
  | |         |  | |   |                               |
  | |         |  | |   |                               |   /-- Proterosuchus go
  | |         |  | |   |   /---------------------------+ /-+
  | |         |  | |   |   |                           | | \-- 'Chasmatosaurus'
  | |         \--+ |   |   |                           \-+
  | |            | |   |   |                             \---- Proterosuchus al
  | |            | |   |   |
  | |            | |   |   |                         /-------- Koilamasuchus go
  | |            | |   |   |                         |
  | |            | |   |   |                         |     /-- Youngosuchus sin
  | |            | |   |   |                         |   /-+
  | |            | |   |   |                         |   | \-- Batrachotomus ku
  | |            | |   |   |                         | /-+
  | |            | |   |   |                       /-+ | \---- Prestosuchus chi
  | |            | |   |   |                       | +-+
  | |            | |   |   |                       | | |   /-- Turfanosuchus da
  | |            | |   |   |                       | | \---+
  | |            | |   |   |                       | |     \-- Gracilisuchus st
  | |            | |   |   |                     /-+ |
  | |            | |   |   |                     | | \-------- Aetosauroides sc
  | |            | |   |   |                     | |
  | |            | |   |   |                     | |       /-- Ornithosuchus lo
  | |            | |   |   |                   /-+ \-------+
  | |            | |   |   |                   | |         \-- Riojasuchus tenu
  | |            | |   |   |                   | |
  | |            | |   |   |                   | \------------ Nundasuchus song
  | |            | |   |   |                /--+
  | |            | |   |   |                |  |           /-- Parasuchus angus
  | |            | |   |   |                |  |         /-+
  | |            \-+   |   |                |  |         | \-- Parasuchus hislo
  | |              |   |   |                |  \---------+
  | |              |   |   |                |            | /-- Nicrosaurus kapf
  | |              |   |   |              /-+            \-+
  | |              |   |   |              | |              \-- Smilosuchus spp.
  \-+              |   |   |              | |
    |              |   |   |              | |      /---------- Dimorphodon macr
    |              |   |   |              | |      |
    |              | /-+   |              | |      | /-------- Lagerpeton chana
    |              | | |   |              | \------+ |
    |              | | |   |              |        | | /------ Marasuchus lillo
    |              | | |   |              |        \-+ |
    |              | | |   |              |          | |   /-- Lewisuchus admix
    |              | | |   |              |          \-+ /-+
    |              | | |   |              |            | | \-- Silesaurus opole
    |              | | |   |              |            \-+
    |              | | |   |              |              | /-- Heterodontosauru
    |              | | |   |              |              \-+
    |              | | |   |            /-+                \-- Herrerasaurus is
    |              | | |   |            | |
    |              | | |   |            | |              /---- Vancleavea campi
    |              | | |   |            | |              |
    |              | | | /-+            | |              +---- Archeopelta arbo
    |              | | | | |            | |              |
    |              | | | | |            | |              +---- Tarjadia ruthae
    |              | | | | |            | |    /---------+
    |              | | | | |            | |    |         | /-- Jaxtasuchus salo
```

+

```
    |              | | | | |           | |    |         +-+
    |              | | | | |           | |    |         | \-- Doswellia kalten
    |              | | | | |           | |    |         |
    |              | | | | |           | |    |         \---- Scleromochlus ta
    |              | | | | |           | |    |
    |              | | | | |           | \----+           /-- Proterochampsa b
    |              | | | | |           |      | /---------+
    |              | | | | |           |      | |         \-- Proterochampsa n
    |              | | | | |         /-+      | |
    |              | | | | |         | |      | |   /-------- Tropidosuchus ro
    |              | | | | |         | |      | |   |
    |              | | | | |         | |      \-+   |   /---- Gualosuchus reig
    |              | | | | |         | |        | /-+   |
    |              | | | | |         | |        | | | /-+ /-- Chanaresuchus bo
    |              | | | | |         | |        | | | | \-+
    |              | | | | |         | |        | | \-+   \-- Rhadinosuchus gr
    |              \-+ | | |         | |        \-+   |
    |                | | | |         | |          |   \------ Pseudochampsa is
    |                | | | |       /-+ |          |
    |                | | | |       | | |          \---------- Cerritosaurus bi
    |                | | | |       | | |
    |                | | | |       | | +--------------------- Euparkeria capen
    |                | \-+ |       | | |
    |                |   | |       | | |                  /-- Yarasuchus decca
    |                |   | |       | | \------------------+
    |                |   | |       | |                    \-- Dongusuchus efre
    |                |   | |     /-+ |
    |                |   | |     | | \----------------------- Dorosuchus neoet
    |                |   | |     | |
    |                |   | |     | |                /-------- Guchengosuchus s
    |                |   | |     | |                |
    |                |   | |     | |                |     /-- GHG 7433MI
    |                |   | |     | |                |     |
    |                |   | |     | \----------------+ /---+-- Garjainia prima
    |                |   | |     |                  | |   |
    |                |   | |   /-+                  | |   \-- Garjainia madiba
    |                |   | |   | |                  \-+
    |                |   | |   | |                    | /---- Erythrosuchus af
    |                |   | |   | |                    | |
    |                |   | |   | |                    \-+ /-- Shansisuchus sha
    |                |   | | /-+ |                      \-+
    |                |   | | | | |                        \-- Chalishevia coth
    |                |   | | | | |
    |                |   | | | | \--------------------------- Cuyosuchus huene
    |                |   | \-+ |
    |                |   |   | \----------------------------- Sarmatosuchus ot
    |                |   |   |
    |                |   |   \------------------------------- Fugusuchus hejia
    |                |   |
    |                |   \----------------------------------- Tasmaniosaurus t
    |                |
    |                \--------------------------------------- Boreopricea fune
    |
    \-------------------------------------------------------- Youngina capensi
```

50% Majority-rule consensus of 1000 trees

```
  /----------------------------------------------------- Petrolacosaurus (1)
  |
  |  /-------------------------------------------------- Acerosodontosaur(2)
  |  |
  |  |                                              /--- Paliguana whitei(4)
  |  |                                              |
  |  | /------------------------------------------100/-- Planocephalosaur(5)
```

+

```
  |100 |                                            100
  || | |                                              \-- Gephyrosaurus br(6)
  || | |
  || | |                                              /-- Cteniogenys sp.(7)
  || 100 /------------------------------------------100
  ||   | |                                            \-- Simoedosaurus le(8)
  ||   | |
  ||   | |/---------------------------------------------- Aenigmastropheus(9)
  ||   | ||
  ||   \89| /-------------------------------------------- Protorosaurus sp(10)
  ||     || |
  ||     || |                                         /-- Amotosaurus rotf(11)
  ||     || |                                       100
  ||     || |                                        |\-- Tanystropheus lo(13)
  ||    100 |                                      100
  ||      | |                                      | \--- Macrocnemus bass(12)
  ||      | | /----------------------------------100
  ||      | | |                                    \----- Jesairosaurus le(14)
  ||      | | |
  ||      | | |                                      /--- Pamelaria dolich(15)
  ||      100 |                                      |
  ||        | |/-----------------------------------100/-- Azendohsaurus ma(16)
  ||        | ||                                    100
  ||        | ||                                      \-- Trilophosaurus b(17)
  ||        | ||
  ||        | ||                               /--------- Noteosuchus coll(18)
  ||        | ||                               |
  ||        | || /---------------------------100 /------- Mesosuchus brown(19)
  ||        | || |                             | |
  ||        | || |                             100 /----- Howesia browni(20)
  ||        | || |                               | |
  ||        100| |                               100 /--- Eohyosaurus wolv(21)
  ||          || |                                 | |
  ||          || |                                 100/-- Rhynchosaurus ar(22)
  ||          || |                                  100
--+|          || |                                    \-- Bentonyx sidensi(23)
  ||          || |
  ||          || |                                    /-- Prolacerta broom(26)
  ||          || |   /------------------------------100
  ||          || |   |                                \-- K australiensis (28)
  ||          || |   |
  ||          || |   |                             /----- Proterosuchus fe(32)
  ||          || |   |                             |
  ||          || |   |                             |  /-- Proterosuchus go(33)
  ||          || |   |  /------------------------100100
  ||          || |   |  |                          | |\-- 'Chasmatosaurus'(35)
  ||           100 |   |  |                          100
  ||             | |   |  |                            \--- Proterosuchus al(34)
  ||             | |   |  |
  ||             | |   |  |                      /--------- Koilamasuchus go(48)
  ||             | |   |  |                      |
  ||             | |   |  |                      |      /-- Youngosuchus sin(62)
  ||             | |   |  |                      |    100
  ||             | |   |  |                      |     |\-- Batrachotomus ku(94)
  ||             | |   |  |                    100   100
  ||             | |   |  |                     ||   | \--- Prestosuchus chi(95)
  ||             | |   |  |                     || 100
  ||             | |   |  |                     || | |  /-- Turfanosuchus da(91)
  ||             | |   |  |                     || | \100
  ||             | |   |  |                   100\78    \-- Gracilisuchus st(92)
  ||             | |   |  |                   | |  |
  ||             | |   |  |                   | |  \------- Aetosauroides sc(93)
  ||             | |   |  |                   | |
  ||             | |   |  |                 100 |       /-- Ornithosuchus lo(88)
```

+

```
 ||           | |   |  |                 | | \-----100
 ||           | |   |  |                 | |         \-- Riojasuchus tenu(89)
 ||           | |   |  |                 | |
 ||           | |   |  |                 | \------------ Nundasuchus song(90)
 ||           | |   |  |              100
 ||           | |   |  |                ||           /-- Parasuchus angus(84)
 ||           | |   |  |                ||         100
 ||           100   |  |                ||          |\-- Parasuchus hislo(85)
 ||             |   |  |                |\--------100
 ||             |   |  |                |           |/-- Nicrosaurus kapf(86)
 ||             |   |  |             100          100
 ||             |   |  |             | |             \-- Smilosuchus spp.(87)
100             |   |  |             | |
  |             |   |  |             | |     /--------- Dimorphodon macr(96)
  |             |   |  |             | |     |
  |             | 100  |             | |     | /------- Lagerpeton chana(97)
  |             | | |  |             | \---100 |
  |             | | |  |             |       | | /----- Marasuchus lillo(98)
  |             | | |  |             |      100 |
  |             | | |  |             |         | |  /-- Lewisuchus admix(99)
  |             | | |  |             |        100100
  |             | | |  |             |           | |\-- Silesaurus opole(100)
  |             | | |  |             |          100
  |             | | |  |             |            |/-- Heterodontosauru(101)
  |             | | |  |             |           100
  |             | | |  |           100             \-- Herrerasaurus is(102)
  |             | | |  |           | |
  |             | | |  |           | |          /----- Vancleavea campi(66)
  |             | | |  |           | |          |
  |             | | |100           | |          |  /-- Archeopelta arbo(80)
  |             | | || |           | |        /66 89
  |             | | || |           | |        | | |\-- Tarjadia ruthae(81)
  |             | | || |           | |        | \78
  |             | | || |           | |        |   |/-- Jaxtasuchus salo(82)
  |             | | || |           | |    /--100  100
  |             | | || |           | |    |     |    \-- Doswellia kalten(83)
  |             | | || |           | |    |     |
  |             | | || |           | |    |     \------- Scleromochlus ta(103)
  |             | | || |           | |    |
  |             | | || |           | |    |         /-- Proterochampsa b(72)
  |             | | || |           | \--100 /-----100
  |             | | || |         100      | |       \-- Proterochampsa n(73)
  |             | | || |         | |      | |
  |             | | || |         | |      | |  /------- Tropidosuchus ro(74)
  |             | | || |         | |      | |  |
  |             | | || |         | |     100   |   /--- Gualosuchus reig(76)
  |             | | || |         | |       |100    |
  |             | | || |         | |       || | 100/-- Chanaresuchus bo(77)
  |             | | || |         | |       || | |100
  |             | | || |         | |       || 100  \-- Rhadinosuchus gr(79)
  |            100 || |       100 |       100   |
  |               | || |       || |        |   \----- Pseudochampsa is(78)
  |               | || |       || |        |
  |               | || |       || |        \--------- Cerritosaurus bi(75)
  |               | || |       || |
  |               | || |       || |               /-- Yarasuchus decca(70)
  |               |100 |      100| \--------------100
  |               |  | |      |  ||               \-- Dongusuchus efre(71)
  |               |  | |      |  ||
  |               |  | |      |  |\-------------------- Euparkeria capen(68)
  |               |  | |      |  |
  |               |  | |      |  \--------------------- Dorosuchus neoet(69)
  |               |  | |    100
  |               |  | |    | |                /------- Guchengosuchus s(52)
```

+

```
   |               |  | |    | |                |
   |               |  | |    | |                |   /--- GHG 7433MI(54)
   |               |  | |    | |                |   |
   |               |  | |    | \--------------100 100/-- Garjainia prima(55)
   |               |  | |    |                  | | 77
   |               |  | |    |                  | |  \-- Garjainia madiba(57)
   |               |  | |  100                  100
   |               |  | |  | |                    | /--- Erythrosuchus af(58)
   |               |  | |  | |                    | |
   |               |  | |  | |                    100/-- Shansisuchus sha(59)
   |               |  | |  | |                     100
   |               |  | |100 |                       \-- Chalishevia coth(61)
   |               |  | | || |
   |               |  | | || \-------------------------- Cuyosuchus huene(53)
   |               |  | 100|
   |               |  |   |\---------------------------- Sarmatosuchus ot(51)
   |               |  |   |
   |               |  |   \----------------------------- Fugusuchus hejia(50)
   |               |  |
   |               |  \--------------------------------- Tasmaniosaurus t(38)
   |               |
   |               \------------------------------------ Boreopricea fune(29)
   |
   \---------------------------------------------------- Youngina capensi(3)
```

Analysis #3 with Chars 558 & 559 Irreversible & Weighted 2

Strict consensus of 1000 trees:

```
   /--------------------------------------------------------- Petrolacosaurus
   |
   |     /--------------------------------------------------- Acerosodontosaur
   |     |
   |     |                                            /------ Paliguana whitei
   |     |                                            |
   |     |   /----------------------------------------+  /--- Planocephalosaur
   |     |   |                                        \--+
   |  /--+   |                                           \--- Gephyrosaurus br
   |  |  |   |
   |  |  |   |                                           /--- Cteniogenys sp.
   |  |  |   |  /----------------------------------------+
   |  |  |   |  |                                        \--- Simoedosaurus le
   |  |  |   |  |
   |  |  \---+  |  /----------------------------------------- Aenigmastropheus
   |  |      |  |  |
   |  |      |  |  +----------------------------------------- Protorosaurus sp
   |  |      |  |  |
   |  |      |  |  |                                     /--- Amotosaurus rotf
   |  |      |  |  |                                  /--+
   |  |      |  |  |                                  |  \--- Tanystropheus lo
   |  |      \--+  |                               /--+
   |  |         |  |                               |  \------ Macrocnemus bass
   |  |         |  |  /----------------------------+
   |  |         |  |  |                            \--------- Jesairosaurus le
   |  |         |  |  |
   |  |         |  |  |                               /------ Pamelaria dolich
   |  |         |  |  |                               |
```

```
+

   |  |         |  |  |            /------------------+  /--- Azendohsaurus ma
   |  |         |  |  |            |                  \--+
   |  |         \--+  |            |                     \--- Trilophosaurus b
   |  |            |  |            |
   |  |            |  |            |                     /--- Prolacerta broom
   |  |            |  |            |     /---------------+
   |  |            |  |            |     |               \--- K australiensis
   |  |            |  |            |     |
   |  |            |  |            |     |         /--------- Proterosuchus fe
   |  |            |  |  /---------+     |         |
   |  |            |  |  |         |     |         |     /--- Proterosuchus go
   |  |            |  |  |         |  /--+     /---+  /--+
   |  |            |  |  |         |  |  |     |   |  |  \--- 'Chasmatosaurus'
   |  |            |  |  |         |  |  |     |   \--+
---+  |            |  |  |         |  |  |  /--+      \------ Proterosuchus al
   |  |            |  |  |         |  |  |  |  |
   |  |            |  |  |         |  |  |  |  |         /--- Fugusuchus hejia
   |  |            \--+  |         \--+  \--+  \---------+
   |  |               |  |            |     |            \--- Sarmatosuchus ot
   |  |               |  |            |     |
   |  |               |  |            |     \---------------- Tasmaniosaurus t
   |  |               |  |            |
   |  |               |  |            \---------------------- Boreopricea fune
   |  |               |  |
   |  |               |  |                  /---------------- Noteosuchus coll
   |  |               |  |                  |
   |  |               |  |  /---------------+  /------------- Mesosuchus brown
   |  |               |  |  |               |  |
   |  |               |  |  |               \--+   /--------- Howesia browni
   |  |               |  |  |                  |   |
   |  |               |  |  |                  \---+  /------ Eohyosaurus wolv
   |  |               |  |  |                      |  |
   |  |               |  |  |                      \--+  /--- Rhynchosaurus ar
   |  |               |  |  |                         \--+
   |  |               |  |  |                            \--- Bentonyx sidensi
   |  |               |  |  |
   |  |               |  |  |            /------------------- Koilamasuchus go
   |  |               |  |  |            |
   |  |               \--+  |            +------------------- Guchengosuchus s
   |  |                  |  |            |
   |  |                  |  |            +------------------- Cuyosuchus huene
   |  |                  |  |            |
   |  |                  |  |            |               /--- GHG 7433MI
   |  |                  |  |            |               |
   |  |                  |  |            |         /-----+--- Garjainia prima
   |  |                  |  |            |         |     |
   |  |                  |  |            |         |     \--- Garjainia madiba
   |  |                  |  |            +---------+
   |  |                  |  |            |         |  /------ Erythrosuchus af
   |  |                  |  |            |         |  |
   |  |                  |  |            |         \--+  /--- Shansisuchus sha
   |  |                  |  |            |            \--+
   |  |                  |  |            |               \--- Chalishevia coth
   |  |                  |  |            |
   |  |                  |  |            +------------------- Youngosuchus sin
   \--+                  |  |            |
      |                  |  |            +------------------- Euparkeria capen
      |                  |  |            |
      |                  |  |            +------------------- Dorosuchus neoet
      |                  |  |            |
      |                  |  |            |               /--- Yarasuchus decca
      |                  |  |            +---------------+
      |                  |  |            |               \--- Dongusuchus efre
      |                  |  |            |
```

```
+

      |                  |  |      /-----+               /--- Parasuchus angus
      |                  |  |      |     |            /--+
      |                  |  |      |     |            |  \--- Parasuchus hislo
      |                  \--+      |     +------------+
      |                     |      |     |            |  /--- Nicrosaurus kapf
      |                     |      |     |            \--+
      |                     |      |     |               \--- Smilosuchus spp.
      |                     |      |     |
      |                     |      |     |               /--- Ornithosuchus lo
      |                     |      |     +---------------+
      |                     |      |     |               \--- Riojasuchus tenu
      |                     |      |     |
      |                     |      |     +------------------- Nundasuchus song
      |                     |      |     |
      |                     |      |     |               /--- Turfanosuchus da
      |                     |      |     +---------------+
      |                     |      |     |               \--- Gracilisuchus st
      |                     |      |     |
      |                     |      |     +------------------- Aetosauroides sc
      |                     |      |     |
      |                     |      |     +------------------- Batrachotomus ku
      |                     |      |     |
      |                     |      |     +------------------- Prestosuchus chi
      |                     |      |     |
      |                     |   /--+     |  /---------------- Dimorphodon macr
      |                     |   |  |     |  |
      |                     |   |  |     |  |  /------------- Lagerpeton chana
      |                     |   |  |     \--+  |
      |                     |   |  |        |  |   /--------- Marasuchus lillo
      |                     |   |  |        \--+   |
      |                     |   |  |           |   |     /--- Lewisuchus admix
      |                     |   |  |           \---+  /--+
      |                     |   |  |               |  |  \--- Silesaurus opole
      |                     |   |  |               \--+
      |                     |   |  |                  |  /--- Heterodontosauru
      |                     |   |  |                  \--+
      |                     |   |  |                     \--- Herrerasaurus is
      |                     |   |  |
      |                     |   |  |               /--------- Vancleavea campi
      |                     |   |  |               |
      |                     |   |  |               |     /--- Archeopelta arbo
      |                     |   |  |  /------------+  /--+
      |                     |   |  |  |            |  |  \--- Tarjadia ruthae
      |                     |   |  |  |            \--+
      |                     \---+  |  |               |  /--- Jaxtasuchus salo
      |                         |  |  |               \--+
      |                         |  |  |                  \--- Doswellia kalten
      |                         |  |  |
      |                         |  \--+                  /--- Proterochampsa b
      |                         |     |  /---------------+
      |                         |     |  |               \--- Proterochampsa n
      |                         |     |  |
      |                         |     |  |     /------------- Tropidosuchus ro
      |                         |     |  |     |
      |                         |     \--+     |      /------ Gualosuchus reig
      |                         |        |  /--+      |
      |                         |        |  |  |   /--+  /--- Chanaresuchus bo
      |                         |        |  |  |   |  \--+
      |                         |        |  |  \---+     \--- Rhadinosuchus gr
      |                         |        \--+      |
      |                         |           |      \--------- Pseudochampsa is
      |                         |           |
      |                         |           \---------------- Cerritosaurus bi
      |                         |
```

```
+


      |                         \---------------------------- Scleromochlus ta
      |
      \------------------------------------------------------ Youngina capensi

50% Majority-rule consensus of 1000 trees

  /----------------------------------------------------- Petrolacosaurus (1)
  |
  |    /------------------------------------------------ Acerosodontosaur(2)
  |    |
  |    |                                          /----- Paliguana whitei(4)
  |    |                                          |
  |    |  /-------------------------------------100 /--- Planocephalosaur(5)
  |    |  |                                       100
  |  100  |                                         \--- Gephyrosaurus br(6)
  |  | |  |
  |  | |  |                                         /--- Cteniogenys sp.(7)
  |  | |  | /-------------------------------------100
  |  | |  | |                                       \--- Simoedosaurus le(8)
  |  | |  | |
  |  | \100 |                                       /--- Aenigmastropheus(9)
  |  |    | |  /----------------------------------79
  |  |    | |  |                                    \--- Protorosaurus sp(10)
  |  |    | |  |
  |  |    | |  |                                    /--- Amotosaurus rotf(11)
  |  |    | |  |                                  100
  |  |    | |  |                                  | \--- Tanystropheus lo(13)
  |  |    100  |                               /100
  |  |      |  |                               |  \----- Macrocnemus bass(12)
  |  |      |  | /---------------------------100
  |  |      |  | |                             \-------- Jesairosaurus le(14)
  |  |      |  | |
  |  |      |  | |                                /----- Pamelaria dolich(15)
  |  |      |  | |                                |
  |  |      |  | |                 /------------100 /--- Azendohsaurus ma(16)
  |  |      |  | |                 |              100
  |  |      |  | |                 |                \--- Trilophosaurus b(17)
  |  |      \100 |                 |
  |  |         | |                 |                /--- Prolacerta broom(26)
  |  |         | |                 |    /---------100
  |  |         | |                 |    |           \--- K australiensis (28)
  |  |         | |                 |    |
  |  |         | |                 |    |      /-------- Proterosuchus fe(32)
  |  |         | |  /------------100    |      |
  |  |         | |  |              |    |      |    /--- Proterosuchus go(33)
  |  |         | |  |              | /100    100  100
  |  |         | |  |              | |  |    | |  | \--- 'Chasmatosaurus'(35)
  |  |         | |  |              | |  |    | \100
--+  |         | |  |              | |  | /100    \----- Proterosuchus al(34)
  |  |         | |  |              | |  | |  |
  |  |         | |  |              | |  | |  |      /--- Fugusuchus hejia(50)
  |  |         | |  |              100  100  \----100
  |  |         100  |                |    |         \--- Sarmatosuchus ot(51)
  |  |           |  |                |    |
  |  |           |  |                |    \------------- Tasmaniosaurus t(38)
  |  |           |  |                |
  |  |           |  |                \------------------ Boreopricea fune(29)
  |  |           |  |
  |  |           |  |                     /------------- Noteosuchus coll(18)
  |  |           |  |                     |
  |  |           |  | /-----------------100  /---------- Mesosuchus brown(19)
  |  |           |  | |                   |  |
  |  |           |  | |                   \100 /-------- Howesia browni(20)
  |  |           |  | |                      | |
```

+

```
|  |           |  | |                      100  /----- Eohyosaurus wolv(21)
|  |           |  | |                        |  |
|  |           |  | |                        \100 /--- Rhynchosaurus ar(22)
|  |           |  | |                           100
|  |           |  | |                             \--- Bentonyx sidensi(23)
|  |           |  | |
|  |           |  | |                        /-------- Koilamasuchus go(48)
|  |           |  | |                        |
|  |           |  | |                        |    /--- Turfanosuchus da(91)
|  |           |  | |                      /76  100
|  |           \100 |                      | |  | \--- Gracilisuchus st(92)
|  |              | |                      | \-92
|  |              | |                      |    | /--- Batrachotomus ku(94)
|  |              | |                   /-92    \92
|  |              | |                   |  |      \--- Prestosuchus chi(95)
|  |              | |                   |  |
|  |              | |                 /92  \---------- Aetosauroides sc(93)
|  |              | |                 | |
|  |              | |                 | |         /--- Ornithosuchus lo(88)
|  |              | |              /-92 \-------100
|  |              | |              |  |           \--- Riojasuchus tenu(89)
|  |              | |              |  |
|  |              | |              |  \--------------- Nundasuchus song(90)
|  |              | |            /92
|  |              | |            | |              /--- Parasuchus angus(84)
\100              | |            | |            100
   |              | |            | |            | \--- Parasuchus hislo(85)
   |              | |            | \----------100
   |              | |            |              | /--- Nicrosaurus kapf(86)
   |              | |         /-92              100
   |              | |         |  |                \--- Smilosuchus spp.(87)
   |              | |         |  |
   |              | |         |  |      /------------- Dimorphodon macr(96)
   |              | |         |  |      |
   |              | |         |  |      |  /---------- Lagerpeton chana(97)
   |              | |         |  \----100  |
   |              | |         |         |  | /-------- Marasuchus lillo(98)
   |              | |         |         \100 |
   |              | |         |            | |    /--- Lewisuchus admix(99)
   |              | |         |            100  100
   |              100         |              |  | \--- Silesaurus opole(100)
   |                |         |              \100
   |                |         |                 | /--- Heterodontosauru(101)
   |                |         |                 100
   |                |       100                 \--- Herrerasaurus is(102)
   |                |       | |
   |                |       | |         /------------- Guchengosuchus s(52)
   |                |       | |         |
   |                |       | |         |  /---------- Cuyosuchus huene(53)
   |                |       | |         |  |
   |                |       | |         |  |    /----- GHG 7433MI(54)
   |                |       | |         |  |    |
   |                |       | |         +-84 /100 /--- Garjainia prima(55)
   |                |       | |         |  | |  \84
   |                |       | |       /84  | |    \--- Garjainia madiba(57)
   |                |       | |       | |  100
   |                |    /100 |       | |    |  /----- Erythrosuchus af(58)
   |                |    |  | |       | |    |  |
   |                |    |  | |       | |    \100 /--- Shansisuchus sha(59)
   |                |    |  | \------92 |       100
   |                |    |  |         | |         \--- Chalishevia coth(61)
   |                |    |  |         | |
   |                |    |  |         | \------------- Dorosuchus neoet(69)
   |                |    |  |         |
```

+

```
|                |    |  |         \--------------- Youngosuchus sin(62)
|                |    |  |
|                |    |  |                   /----- Euparkeria capen(68)
|                |    |  |                   |
|                |    |  \-----------------100 /--- Yarasuchus decca(70)
|                |  100                      100
|                |  | |                        \--- Dongusuchus efre(71)
|                |  | |
|                |  | |                   /-------- Vancleavea campi(66)
|                |  | |                   |
|                |  | |                   |    /--- Archeopelta arbo(80)
|                |  | |         /-------100  100
|                |  | |         |         |  | \--- Tarjadia ruthae(81)
|                |  | |         |         \100
|                |  | |         |            | /--- Jaxtasuchus salo(82)
|                |  | |         |            100
|                |  | |         |              \--- Doswellia kalten(83)
|                |  | |         |
|                |  | \-------100              /--- Proterochampsa b(72)
|                |  |           |  /---------100
|                \100           |  |           \--- Proterochampsa n(73)
|                   |           |  |
|                   |           |  |    /---------- Tropidosuchus ro(74)
|                   |           |  |    |
|                   |           \100    |    /----- Gualosuchus reig(76)
|                   |              | /100    |
|                   |              | |  | /100 /--- Chanaresuchus bo(77)
|                   |              | |  | |  100
|                   |              | |  100    \--- Rhadinosuchus gr(79)
|                   |              100    |
|                   |                |    \-------- Pseudochampsa is(78)
|                   |                |
|                   |                \------------- Cerritosaurus bi(75)
|                   |
|                   \------------------------------ Scleromochlus ta(103)
|
\-------------------------------------------------- Youngina capensi(3)
```
